# Supplementary material for: Antidepressants Target the ST3GAL5–GM3 Lipid Pathway to Suppress Microglial Inflammation
Source: Int J Mol Sci. 2025 Oct 7;26(19):9733. doi: 10.3390/ijms26199733 (PMC12524792; doi:10.3390/ijms26199733)
Supplement: Supplementary file 1 [file ijms-26-09733-s001.zip › ijms-3869284-supplementary.pdf]

Supplementary Materials
Supplementary Figures

Fig. S1

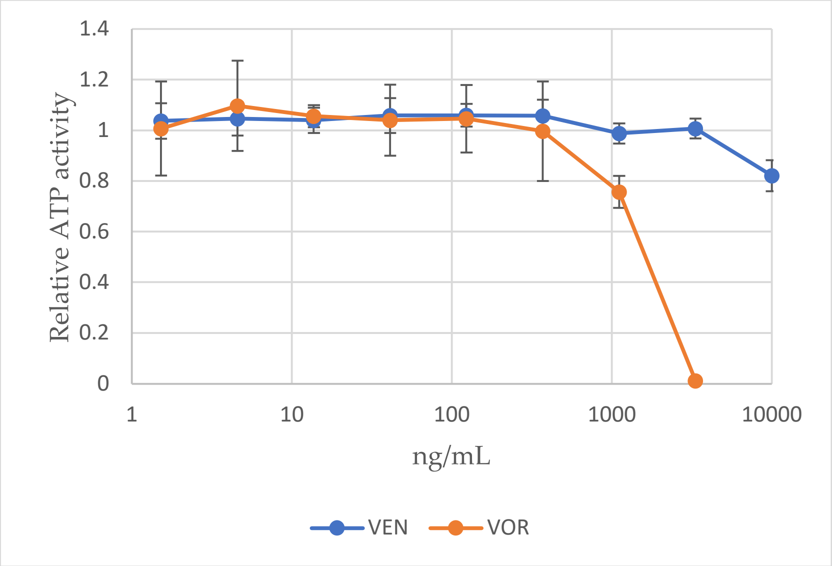

Supplementary Fig. S1. Cytotoxicity of venlafaxine (VEN) and vortioxetine (VOR).
Methods: Microglial cells were seeded into 96-well plates (1×10^3 cells/well) and exposed for 72 h to increasing concentrations of VEN (0–1000 ng/mL) or VOR (0–1000 ng/mL). Cell viability was assessed using the CellTiter-Glo® ATP assay.
Results: Both VEN and VOR showed no significant cytotoxicity up to 200 ng/mL and 40 ng/mL, respectively, supporting the use of these concentrations for transcriptomic analyses.

Fig.S2

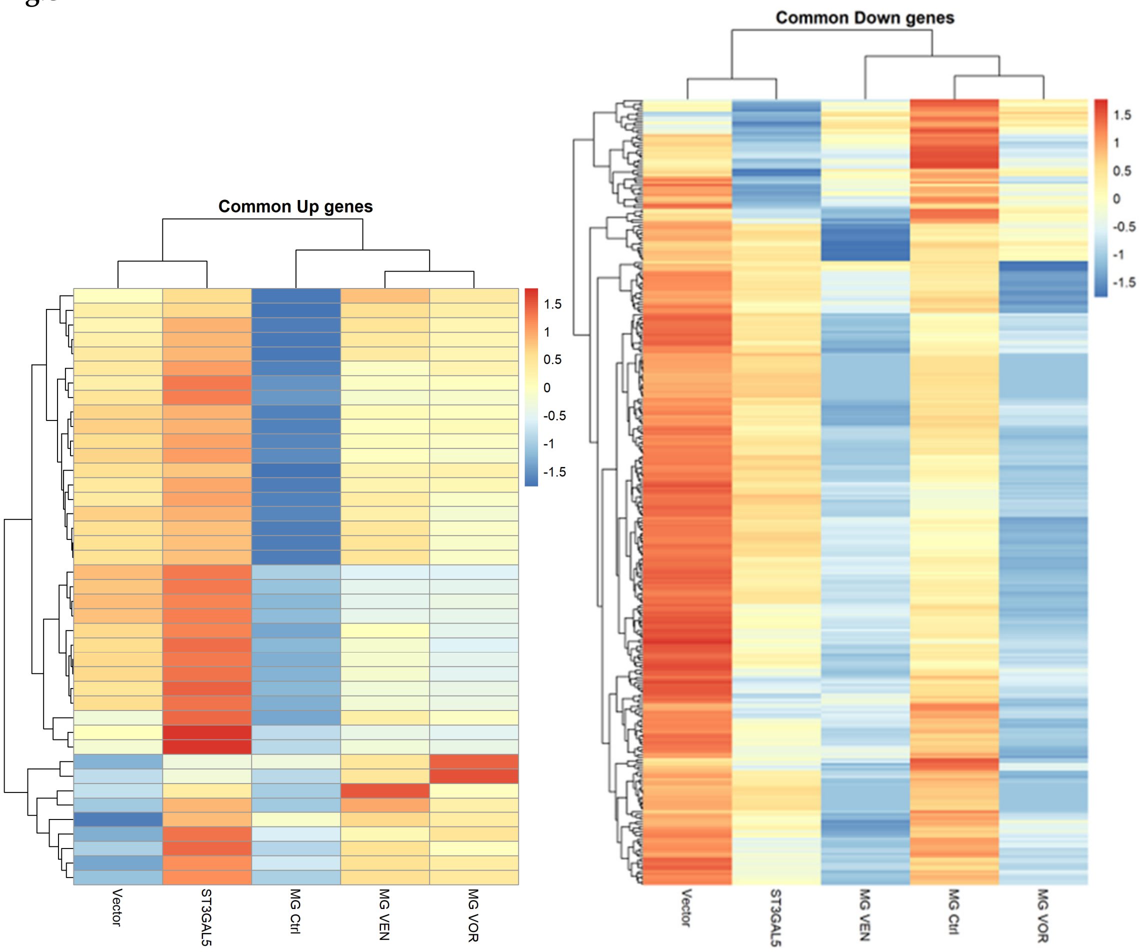

Figure S2. Representative common gene expression profiles.

Heatmap showing representative genes consistently regulated across VEN, VOR, and ST3GAL5OE. Examples include APOE (upregulated), ST3GAL5 probe signal (decreased in array but validated by qRT-PCR), C3, HSPA5, DDIT3, and TREM2. Expression values are scaled by row

Fig. S3. GSEA plots for VEN and VOR transcriptomes.

(A)

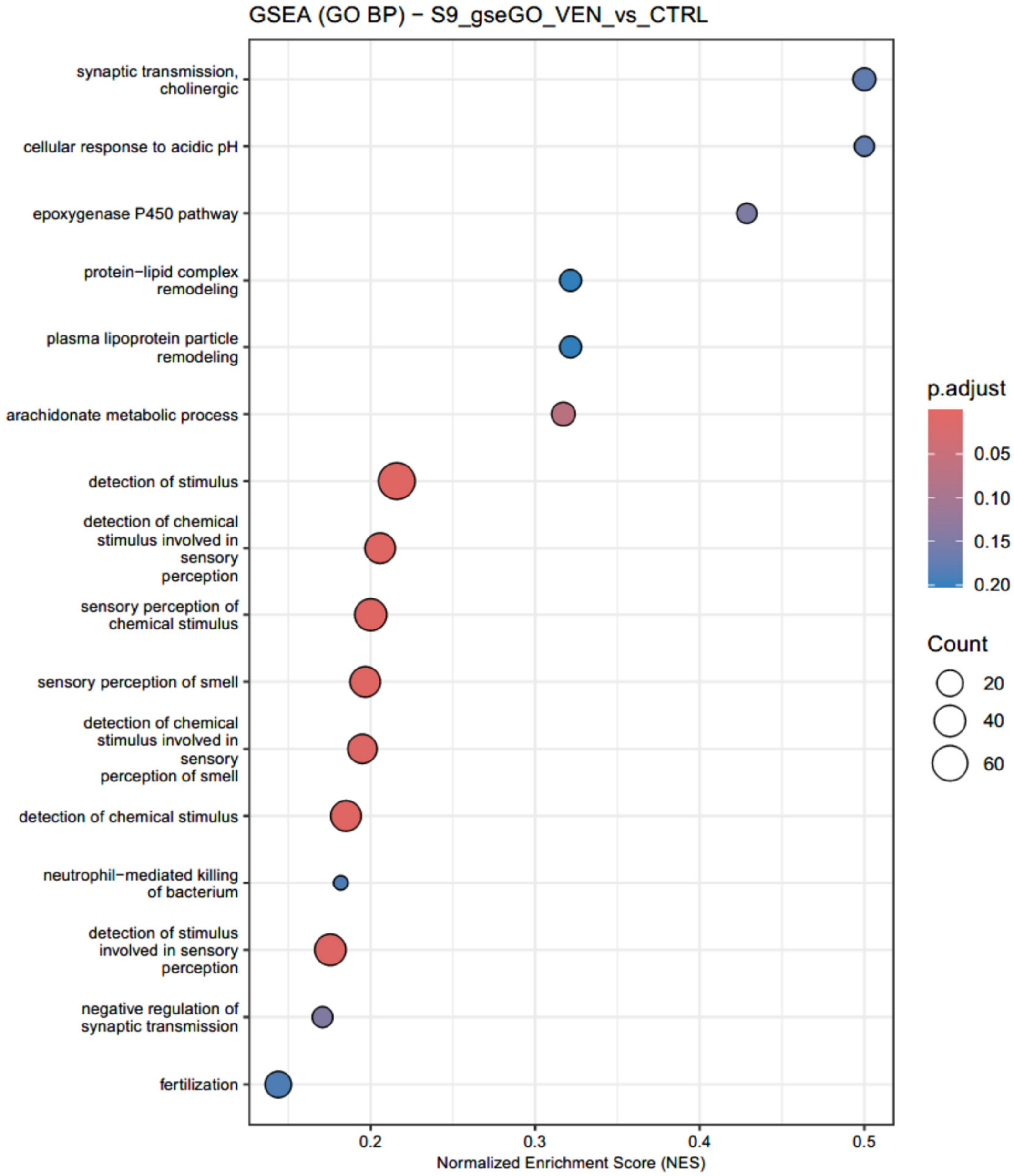

(B)

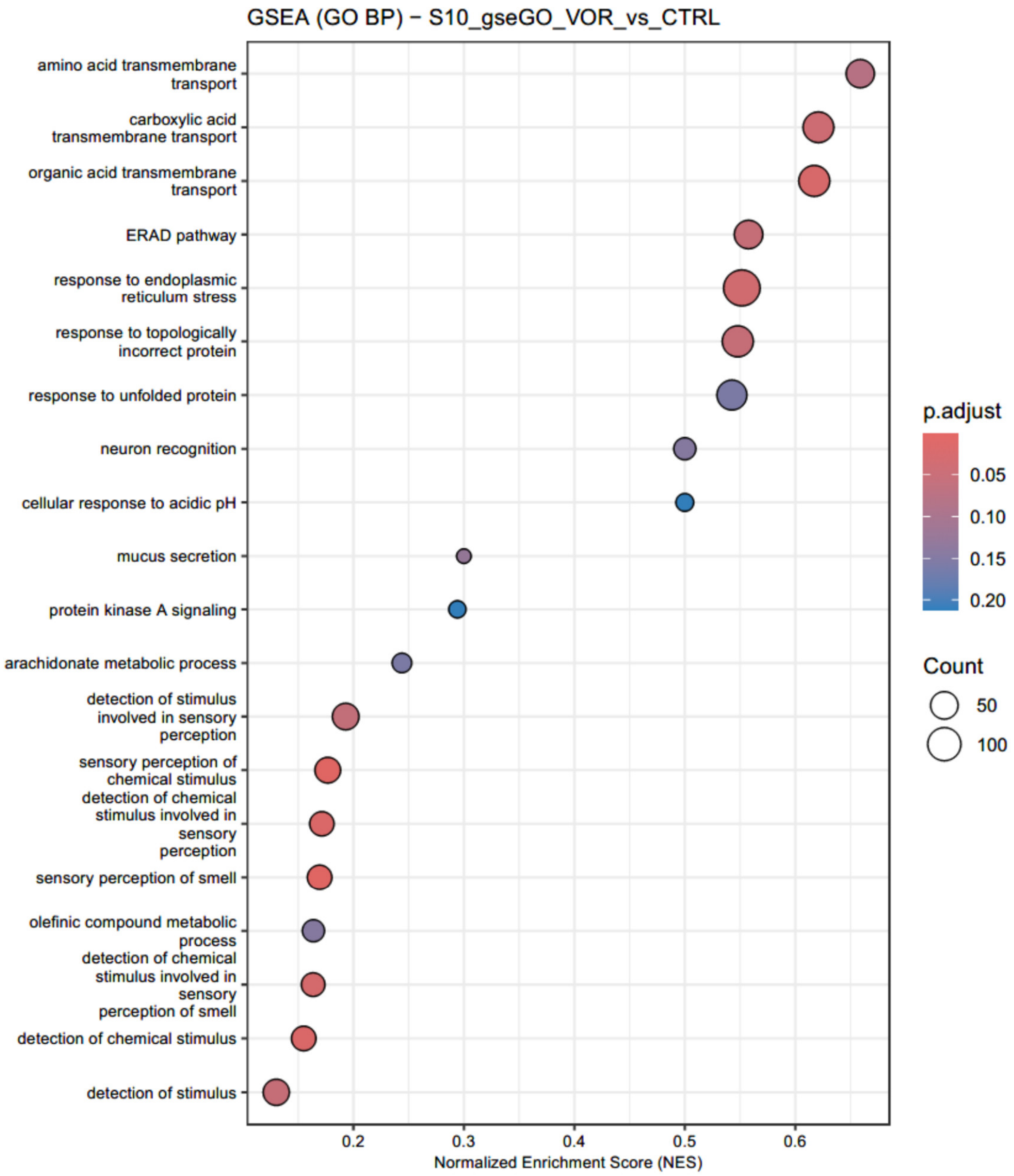

Supplementary Fig. S3. GSEA plots for VEN and VOR transcriptomes.

Methods: Gene set enrichment analysis (GSEA) was conducted on log2FC-ranked gene lists for venlafaxine (VEN) and vortioxetine (VOR) using GO Biological Process and/or KEGG gene sets (clusterProfiler).

Results: Representative enrichment plots illustrate significant regulation of synaptic organization and attenuation of inflammatory signaling under both drug conditions.

Fig. S4. ATP viability assays following TNF-α and IL-6 titrations

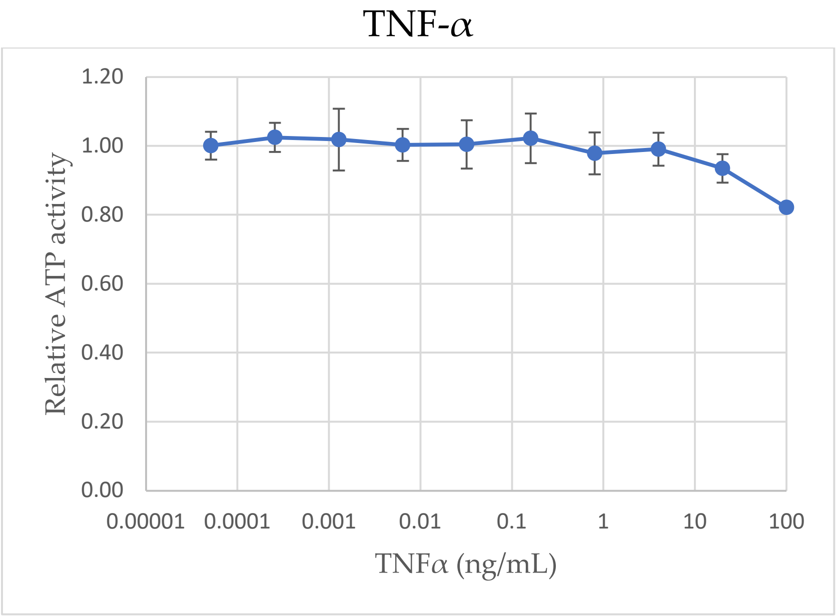

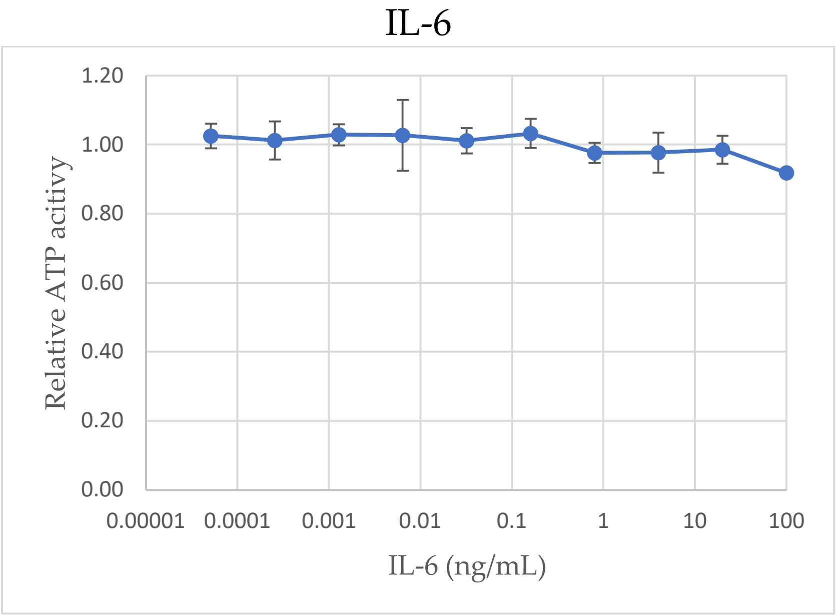

Supplementary Fig. S4. ATP viability assays following TNF-α and IL-6 titrations.

Methods: Microglial cells were exposed to TNF-α (0–10 ng/mL) or IL-6 (0–10 ng/mL) for 72 h, and cell viability was assessed using the ATP-based CellTiter-Glo® assay.

Results: Cytokine treatments were generally well tolerated, with only a modest reduction in cell viability (~85%) observed at the highest TNF-α concentration (10 ng/mL), whereas IL-6 showed little to no cytotoxicity across the tested range. Thus, reporter activation was largely independent of viability effects.

Fig. S5. Real-time monitoring of NF-κB and STAT3 reporter activity after cytokine stimulation (0–24 h).

TNF-α

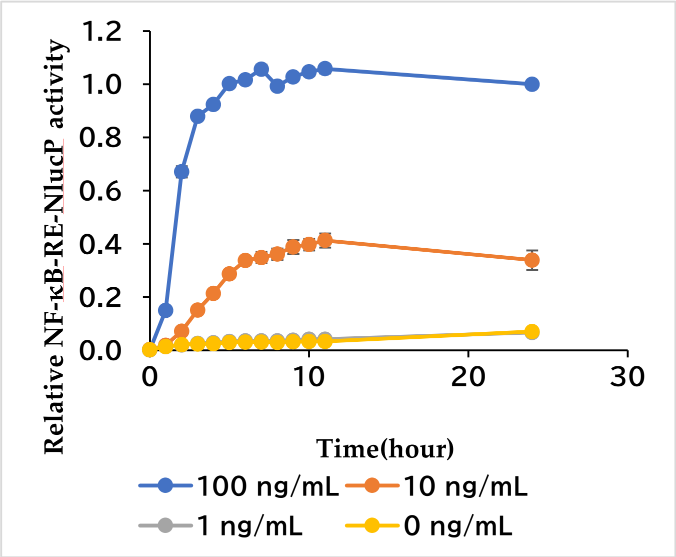

IL-6

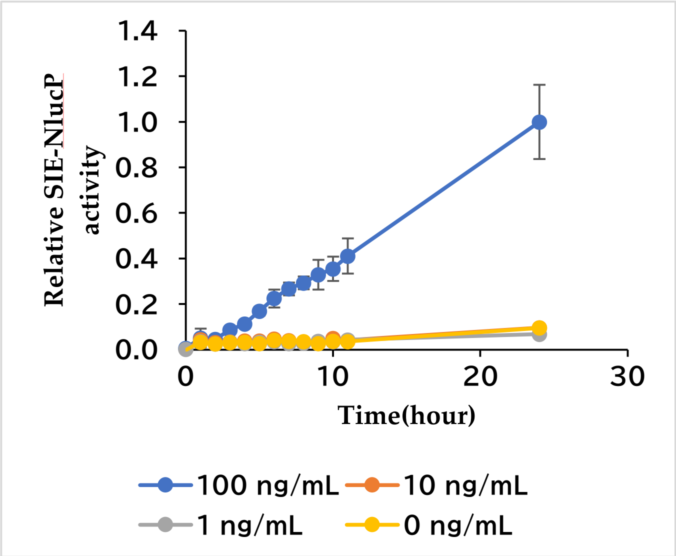

Supplementary Fig. S3. Real-time monitoring of NF-κB and STAT3 reporter activity after cytokine stimulation.

Methods: Microglial cells stably expressing NF-κB-RE-NlucP or SIE-NlucP were treated with TNF-α (0–10 ng/mL) or IL-6 (0–50 ng/mL). Luminescence was monitored every hour for 12 h and at 24 h using the Nano-Glo® Endurazine™ substrate.

Results: TNF-α induced rapid reporter activation within 2–3 h, peaking at ~6–8 h and sustaining activity up to 24 h. In contrast, IL-6 elicited a gradual increase in reporter activity that continued to rise throughout the 24-h period, indicating a slower but sustained activation profile.

Fig. S6. GM3 cytotoxicity assay (0–50 μM).

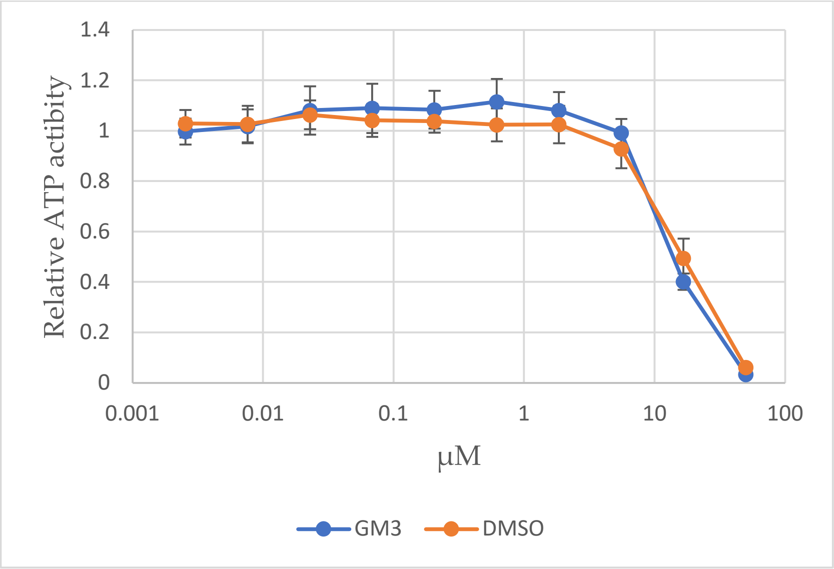

Fig. S6. GM3 cytotoxicity and vehicle control.

Methods: MG cells were seeded into 96-well plates (1×10^3 cells/well) and treated with GM3 (0–50 μM, 3-fold serial dilutions) or DMSO vehicle control for 72 h. ATP levels were measured using the CellTiter-Glo® assay.

Results: At higher doses, reductions in viability mirrored those produced by the corresponding DMSO vehicle, indicating no additional cytotoxicity attributable to GM3 within the tested range. Thus, GM3 showed no extra toxicity beyond DMSO under these conditions.

Supplementary Tables

- **Table S1.** Commonly upregulated GO Biological Processes (full list).

ID	Description	p.adjust	Count
GO:0007158	neuron cell-cell adhesion	0.00171	3
GO:0099175	regulation of postsynapse organization	0.020244	4
GO:0050807	regulation of synapse organization	0.020244	5
GO:0097090	presynaptic membrane organization	0.020244	2
GO:0050803	regulation of synapse structure or activity	0.020244	5
GO:0051963	regulation of synapse assembly	0.020244	4
GO:0098693	regulation of synaptic vesicle cycle	0.030785	2
GO:0099068	postsynapse assembly	0.030952	3
GO:0060644	mammary gland epithelial cell differentiation	0.030952	2
GO:0099504	synaptic vesicle cycle	0.034402	4
GO:0060996	dendritic spine development	0.039812	3
GO:0099173	postsynapse organization	0.040304	4
GO:0051960	regulation of nervous system development	0.04324	5
GO:0099003	vesicle-mediated transport in synapse	0.04324	4
GO:1901888	regulation of cell junction assembly	0.046711	4
GO:0007416	synapse assembly	0.046711	4
GO:0051962	positive regulation of nervous system development	0.051578	4
GO:0051491	positive regulation of filopodium assembly	0.052184	2
GO:0048511	rhythmic process	0.056184	4
GO:0030879	mammary gland development	0.056184	3
GO:1904861	excitatory synapse assembly	0.077786	2
GO:0060999	positive regulation of dendritic spine development	0.077786	2
GO:0001825	blastocyst formation	0.077786	2
GO:0061001	regulation of dendritic spine morphogenesis	0.077786	2
GO:0048512	circadian behavior	0.077786	2
GO:0097106	postsynaptic density organization	0.077786	2
GO:0007622	rhythmic behavior	0.077786	2
GO:0099054	presynapse assembly	0.077786	2
GO:0050767	regulation of neurogenesis	0.077786	4
GO:0051489	regulation of filopodium assembly	0.077786	2

GO:0098815	modulation of excitatory postsynaptic potential	0.078336	2
GO:0099084	postsynaptic specialization organization	0.091541	2
GO:0045664	regulation of neuron differentiation	0.092612	3
GO:0099172	presynapse organization	0.092612	2
GO:0000079	regulation of cyclin-dependent protein serine/threonine kinase activity	0.092612	2
GO:0060997	dendritic spine morphogenesis	0.092612	2
GO:0060998	regulation of dendritic spine development	0.092612	2
GO:0120032	regulation of plasma membrane bounded cell projection assembly	0.092612	3
GO:0060491	regulation of cell projection assembly	0.092612	3
GO:0071709	membrane assembly	0.092612	2
GO:0001756	somitogenesis	0.09909	2
GO:0044091	membrane biogenesis	0.103731	2
GO:0046847	filopodium assembly	0.103731	2
GO:0061180	mammary gland epithelium development	0.103731	2
GO:0050769	positive regulation of neurogenesis	0.113179	3
GO:0000082	G1/S transition of mitotic cell cycle	0.11873	3
GO:0016358	dendrite development	0.118891	3
GO:0097120	receptor localization to synapse	0.120639	2
GO:0010469	regulation of signaling receptor activity	0.12411	2
GO:0061053	somite development	0.130594	2
GO:0097061	dendritic spine organization	0.137108	2
GO:0035418	protein localization to synapse	0.140559	2
GO:0044843	cell cycle G1/S phase transition	0.143732	3
GO:0140029	exocytic process	0.144366	2
GO:0007589	body fluid secretion	0.153941	2
GO:0106027	neuron projection organization	0.154256	2
GO:0048661	positive regulation of smooth muscle cell proliferation	0.159864	2
GO:0016079	synaptic vesicle exocytosis	0.159864	2
GO:0035282	segmentation	0.159864	2
GO:0021549	cerebellum development	0.159864	2
GO:0060079	excitatory postsynaptic potential	0.159864	2
GO:0006029	proteoglycan metabolic process	0.159864	2

GO:0042752	regulation of circadian rhythm	0.159864	2
GO:0120034	positive regulation of plasma membrane bounded cell projection assembly	0.159864	2
GO:0044248	cellular catabolic process	0.159864	3
GO:0001824	blastocyst development	0.159864	2
GO:0022037	metencephalon development	0.159864	2
GO:0099565	chemical synaptic transmission, postsynaptic	0.159864	2
GO:0007250	activation of NF-kappaB-inducing kinase activity	0.159864	1
GO:0019614	catechol-containing compound catabolic process	0.159864	1
GO:0042420	dopamine catabolic process	0.159864	1
GO:0042424	catecholamine catabolic process	0.159864	1
GO:0097113	AMPA glutamate receptor clustering	0.159864	1
GO:0097119	postsynaptic density protein 95 clustering	0.159864	1
GO:0097688	glutamate receptor clustering	0.159864	1
GO:1902414	protein localization to cell junction	0.159864	2
GO:0031644	regulation of nervous system process	0.159864	2
GO:0009068	aspartate family amino acid catabolic process	0.159864	1
GO:0021548	pons development	0.159864	1
GO:0090394	negative regulation of excitatory postsynaptic potential	0.159864	1
GO:0099624	atrial cardiac muscle cell membrane repolarization	0.159864	1
GO:0019336	phenol-containing compound catabolic process	0.159864	1
GO:2000310	regulation of NMDA receptor activity	0.159864	1
GO:0031346	positive regulation of cell projection organization	0.159864	3
GO:0048565	digestive tract development	0.159864	2
GO:0006012	galactose metabolic process	0.159864	1
GO:0006544	glycine metabolic process	0.159864	1
GO:0008063	Toll signaling pathway	0.159864	1
GO:0009415	response to water	0.159864	1
GO:0032530	regulation of microvillus organization	0.159864	1

GO:0035589	G protein-coupled purinergic nucleotide receptor signaling pathway	0.159864	1
GO:0048711	positive regulation of astrocyte differentiation	0.159864	1
GO:0045859	regulation of protein kinase activity	0.159864	3
GO:0043433	negative regulation of DNA-binding transcription factor activity	0.159864	2
GO:0018105	peptidyl-serine phosphorylation	0.159864	2
GO:0048813	dendrite morphogenesis	0.159864	2
GO:0043353	enucleate erythrocyte differentiation	0.159864	1
GO:0061000	negative regulation of dendritic spine development	0.159864	1
GO:1902259	regulation of delayed rectifier potassium channel activity	0.159864	1
GO:2000311	regulation of AMPA receptor activity	0.159864	1
GO:0001101	response to acid chemical	0.159864	2
GO:1900180	regulation of protein localization to nucleus	0.159864	2
GO:0007269	neurotransmitter secretion	0.159864	2
GO:0099643	signal release from synapse	0.159864	2
GO:0045348	positive regulation of MHC class II biosynthetic process	0.159864	1
GO:0098969	neurotransmitter receptor transport to postsynaptic membrane	0.159864	1
GO:1900029	positive regulation of ruffle assembly	0.159864	1
GO:0060078	regulation of postsynaptic membrane potential	0.159864	2
GO:0055123	digestive system development	0.159864	2
GO:0043549	regulation of kinase activity	0.159864	3
GO:0018209	peptidyl-serine modification	0.159864	2
GO:0018146	keratan sulfate proteoglycan biosynthetic process	0.159864	1
GO:0021819	layer formation in cerebral cortex	0.159864	1
GO:0030033	microvillus assembly	0.159864	1
GO:0030167	proteoglycan catabolic process	0.159864	1
GO:0030214	hyaluronan catabolic process	0.159864	1
GO:0032230	positive regulation of synaptic transmission, GABAergic	0.159864	1

GO:0098877	neurotransmitter receptor transport to plasma membrane	0.159864	1
GO:1903540	establishment of protein localization to postsynaptic membrane	0.159864	1
GO:0045651	positive regulation of macrophage differentiation	0.159864	1
GO:0048557	embryonic digestive tract morphogenesis	0.159864	1
GO:0070254	mucus secretion	0.159864	1
GO:0071468	cellular response to acidic pH	0.159864	1
GO:0097091	synaptic vesicle clustering	0.159864	1
GO:0098915	membrane repolarization during ventricular cardiac muscle cell action potential	0.159864	1
GO:0099151	regulation of postsynaptic density assembly	0.159864	1
GO:1903817	negative regulation of voltage-gated potassium channel activity	0.159864	1
GO:0030902	hindbrain development	0.159864	2
GO:0001956	positive regulation of neurotransmitter secretion	0.159864	1
GO:0006044	N-acetylglucosamine metabolic process	0.159864	1
GO:0042339	keratan sulfate proteoglycan metabolic process	0.159864	1
GO:0042753	positive regulation of circadian rhythm	0.159864	1
GO:0045475	locomotor rhythm	0.159864	1
GO:0072578	neurotransmitter-gated ion channel clustering	0.159864	1
GO:0032412	regulation of monoatomic ion transmembrane transporter activity	0.159864	2
GO:0007584	response to nutrient	0.159864	2
GO:1990845	adaptive thermogenesis	0.159864	2
GO:0001829	trophectodermal cell differentiation	0.159864	1
GO:0036342	post-anal tail morphogenesis	0.159864	1
GO:0042402	biogenic amine catabolic process	0.159864	1
GO:0042749	regulation of circadian sleep/wake cycle	0.159864	1
GO:0045346	regulation of MHC class II biosynthetic process	0.159864	1
GO:0071318	cellular response to ATP	0.159864	1
GO:0086014	atrial cardiac muscle cell action potential	0.159864	1
GO:0086026	atrial cardiac muscle cell to AV node cell signaling	0.159864	1

GO:0086066	atrial cardiac muscle cell to AV node cell communication	0.159864	1
GO:0048660	regulation of smooth muscle cell proliferation	0.159864	2
GO:0002755	MyD88-dependent toll-like receptor signaling pathway	0.159864	1
GO:0022410	circadian sleep/wake cycle process	0.159864	1
GO:0034162	toll-like receptor 9 signaling pathway	0.159864	1
GO:0043171	peptide catabolic process	0.159864	1
GO:0045342	MHC class II biosynthetic process	0.159864	1
GO:0045472	response to ether	0.159864	1
GO:0045736	negative regulation of cyclin-dependent protein serine/threonine kinase activity	0.159864	1
GO:0060749	mammary gland alveolus development	0.159864	1
GO:0061377	mammary gland lobule development	0.159864	1
GO:0070314	G1 to G0 transition	0.159864	1
GO:0071625	vocalization behavior	0.159864	1
GO:0009266	response to temperature stimulus	0.159864	2
GO:0022898	regulation of transmembrane transporter activity	0.159864	2
GO:2000045	regulation of G1/S transition of mitotic cell cycle	0.159864	2
GO:0030216	keratinocyte differentiation	0.159864	2
GO:0048659	smooth muscle cell proliferation	0.159864	2
GO:0007213	G protein-coupled acetylcholine receptor signaling pathway	0.159864	1
GO:0007413	axonal fasciculation	0.159864	1
GO:0009310	amine catabolic process	0.159864	1
GO:0097623	potassium ion export across plasma membrane	0.159864	1
GO:0099150	regulation of postsynaptic specialization assembly	0.159864	1
GO:0106030	neuron projection fasciculation	0.159864	1
GO:1905874	regulation of postsynaptic density organization	0.159864	1
GO:2000273	positive regulation of signaling receptor activity	0.159864	1
GO:0035265	organ growth	0.159864	2
GO:0010720	positive regulation of cell development	0.159864	3

GO:0010975	regulation of neuron projection development	0.159864	3
GO:0006417	regulation of translation	0.159864	3
GO:0048732	gland development	0.159864	3
GO:0031630	regulation of synaptic vesicle fusion to presynaptic active zone membrane	0.159864	1
GO:1900242	regulation of synaptic vesicle endocytosis	0.159864	1
GO:1901017	negative regulation of potassium ion transmembrane transporter activity	0.159864	1
GO:1901632	regulation of synaptic vesicle membrane organization	0.159864	1
GO:1904030	negative regulation of cyclin-dependent protein kinase activity	0.159864	1
GO:1904889	regulation of excitatory synapse assembly	0.159864	1
GO:0017157	regulation of exocytosis	0.159864	2
GO:0002753	cytoplasmic pattern recognition receptor signaling pathway	0.161995	2
GO:0044772	mitotic cell cycle phase transition	0.161995	3
GO:0007219	Notch signaling pathway	0.161995	2
GO:0042745	circadian sleep/wake cycle	0.163019	1
GO:0042391	regulation of membrane potential	0.164624	3
GO:0051338	regulation of transferase activity	0.164624	3
GO:0032528	microvillus organization	0.164692	1
GO:0043153	entrainment of circadian clock by photoperiod	0.164692	1
GO:0051590	positive regulation of neurotransmitter transport	0.164692	1
GO:0060307	regulation of ventricular cardiac muscle cell membrane repolarization	0.164692	1
GO:0048169	regulation of long-term neuronal synaptic plasticity	0.165937	1
GO:0090280	positive regulation of calcium ion import	0.165937	1
GO:1901071	glucosamine-containing compound metabolic process	0.165937	1
GO:1905475	regulation of protein localization to membrane	0.165937	2
GO:0030317	flagellated sperm motility	0.165937	2
GO:0097722	sperm motility	0.165937	2
GO:0032271	regulation of protein polymerization	0.165937	2

GO:0006027	glycosaminoglycan catabolic process	0.165937	1
GO:0033598	mammary gland epithelial cell proliferation	0.165937	1
GO:0048520	positive regulation of behavior	0.165937	1
GO:0090128	regulation of synapse maturation	0.165937	1
GO:0090314	positive regulation of protein targeting to membrane	0.165937	1
GO:0099637	neurotransmitter receptor transport	0.165937	1
GO:1900227	positive regulation of NLRP3 inflammasome complex assembly	0.165937	1
GO:0032409	regulation of transporter activity	0.165937	2
GO:0031334	positive regulation of protein-containing complex assembly	0.165937	2
GO:1902806	regulation of cell cycle G1/S phase transition	0.165937	2
GO:0009648	photoperiodism	0.165937	1
GO:0035235	ionotropic glutamate receptor signaling pathway	0.165937	1
GO:0045649	regulation of macrophage differentiation	0.165937	1
GO:0071467	cellular response to pH	0.165937	1
GO:0141087	positive regulation of inflammasome-mediated signaling pathway	0.165937	1
GO:0007623	circadian rhythm	0.16635	2
GO:0000303	response to superoxide	0.16635	1
GO:0021801	cerebral cortex radial glia-guided migration	0.16635	1
GO:0022030	telencephalon glial cell migration	0.16635	1
GO:0060575	intestinal epithelial cell differentiation	0.16635	1
GO:0086013	membrane repolarization during cardiac muscle cell action potential	0.16635	1
GO:0043270	positive regulation of monoatomic ion transport	0.16635	2
GO:0006836	neurotransmitter transport	0.16635	2
GO:0000305	response to oxygen radical	0.16635	1
GO:0009649	entrainment of circadian clock	0.16635	1
GO:0010447	response to acidic pH	0.16635	1
GO:0097107	postsynaptic density assembly	0.16635	1
GO:1901380	negative regulation of potassium ion transmembrane transport	0.16635	1
GO:1902683	regulation of receptor localization to synapse	0.16635	1

GO:1903421	regulation of synaptic vesicle recycling	0.16635	1
GO:0043123	positive regulation of canonical NF-kappaB signal transduction	0.166495	2
GO:0031503	protein-containing complex localization	0.166679	2
GO:0071805	potassium ion transmembrane transport	0.166679	2
GO:0060294	cilium movement involved in cell motility	0.166679	2
GO:0071900	regulation of protein serine/threonine kinase activity	0.166679	2
GO:0099623	regulation of cardiac muscle cell membrane repolarization	0.166679	1
GO:0099625	ventricular cardiac muscle cell membrane repolarization	0.166679	1
GO:0009952	anterior/posterior pattern specification	0.166679	2
GO:0001539	cilium or flagellum-dependent cell motility	0.166679	2
GO:0060285	cilium-dependent cell motility	0.166679	2
GO:0007274	neuromuscular synaptic transmission	0.166679	1
GO:0031629	synaptic vesicle fusion to presynaptic active zone membrane	0.166679	1
GO:0050654	chondroitin sulfate proteoglycan metabolic process	0.166679	1
GO:0086019	cell-cell signaling involved in cardiac conduction	0.166679	1
GO:1900027	regulation of ruffle assembly	0.166679	1
GO:0006026	aminoglycan catabolic process	0.167815	1
GO:0009069	serine family amino acid metabolic process	0.167815	1
GO:0035590	purinergic nucleotide receptor signaling pathway	0.167815	1
GO:0090313	regulation of protein targeting to membrane	0.167815	1
GO:0099500	vesicle fusion to plasma membrane	0.167815	1
GO:0099601	regulation of neurotransmitter receptor activity	0.167815	1
GO:0007163	establishment or maintenance of cell polarity	0.168235	2
GO:0045055	regulated exocytosis	0.168235	2
GO:0007288	sperm axoneme assembly	0.168235	1
GO:0031116	positive regulation of microtubule polymerization	0.168235	1
GO:0033198	response to ATP	0.168235	1

GO:0048566	embryonic digestive tract development	0.168235	1
GO:0086011	membrane repolarization during action potential	0.168235	1
GO:0030212	hyaluronan metabolic process	0.169907	1
GO:0033146	regulation of intracellular estrogen receptor signaling pathway	0.169907	1
GO:0048710	regulation of astrocyte differentiation	0.169907	1
GO:0050869	negative regulation of B cell activation	0.169907	1
GO:0070498	interleukin-1-mediated signaling pathway	0.169907	1
GO:0006654	phosphatidic acid biosynthetic process	0.170239	1
GO:0021772	olfactory bulb development	0.170239	1
GO:0031338	regulation of vesicle fusion	0.170239	1
GO:0043267	negative regulation of potassium ion transport	0.170239	1
GO:0045777	positive regulation of blood pressure	0.170239	1
GO:0086005	ventricular cardiac muscle cell action potential	0.170239	1
GO:2000463	positive regulation of excitatory postsynaptic potential	0.170239	1
GO:0045577	regulation of B cell differentiation	0.171166	1
GO:0048499	synaptic vesicle membrane organization	0.171166	1
GO:0051968	positive regulation of synaptic transmission, glutamatergic	0.171166	1
GO:0098698	postsynaptic specialization assembly	0.171166	1
GO:0140058	neuron projection arborization	0.171166	1
GO:1901381	positive regulation of potassium ion transmembrane transport	0.171166	1
GO:0003161	cardiac conduction system development	0.172658	1
GO:0021799	cerebral cortex radially oriented cell migration	0.172658	1
GO:0021988	olfactory lobe development	0.172658	1
GO:0032228	regulation of synaptic transmission, GABAergic	0.172658	1
GO:0045773	positive regulation of axon extension	0.172658	1
GO:0006813	potassium ion transport	0.17355	2
GO:0031112	positive regulation of microtubule polymerization or depolymerization	0.175317	1
GO:1900181	negative regulation of protein localization to nucleus	0.175317	1
GO:0002446	neutrophil mediated immunity	0.176727	1

GO:0006516	glycoprotein catabolic process	0.176727	1
GO:0046473	phosphatidic acid metabolic process	0.176727	1
GO:0009913	epidermal cell differentiation	0.176727	2
GO:0033002	muscle cell proliferation	0.176727	2
GO:0001941	postsynaptic membrane organization	0.179214	1
GO:0060074	synapse maturation	0.179214	1
GO:0086091	regulation of heart rate by cardiac conduction	0.179214	1
GO:0009066	aspartate family amino acid metabolic process	0.182305	1
GO:1990806	ligand-gated ion channel signaling pathway	0.182305	1
GO:0003341	cilium movement	0.183419	2
GO:0006040	amino sugar metabolic process	0.183419	1
GO:0042417	dopamine metabolic process	0.183419	1
GO:0060306	regulation of membrane repolarization	0.183419	1
GO:0099622	cardiac muscle cell membrane repolarization	0.183419	1
GO:0007595	lactation	0.183419	1
GO:0009268	response to pH	0.183419	1
GO:0043268	positive regulation of potassium ion transport	0.183419	1
GO:0045746	negative regulation of Notch signaling pathway	0.183419	1
GO:0150052	regulation of postsynapse assembly	0.183419	1
GO:1900225	regulation of NLRP3 inflammasome complex assembly	0.183419	1
GO:2001258	negative regulation of cation channel activity	0.183419	1
GO:0009409	response to cold	0.184526	1
GO:0050912	detection of chemical stimulus involved in sensory perception of taste	0.184526	1
GO:0097178	ruffle assembly	0.184526	1
GO:0098703	calcium ion import across plasma membrane	0.184526	1
GO:1902656	calcium ion import into cytosol	0.184526	1
GO:0045687	positive regulation of glial cell differentiation	0.186774	1
GO:0090051	negative regulation of cell migration involved in sprouting angiogenesis	0.186774	1
GO:1901016	regulation of potassium ion transmembrane transporter activity	0.186774	1
GO:0044546	NLRP3 inflammasome complex assembly	0.188974	1
GO:0060711	labyrinthine layer development	0.188974	1

GO:0095500	acetylcholine receptor signaling pathway	0.188974	1
GO:0007157	heterophilic cell-cell adhesion via plasma membrane cell adhesion molecules	0.189087	1
GO:0032941	secretion by tissue	0.189087	1
GO:0044273	sulfur compound catabolic process	0.189087	1
GO:1903307	positive regulation of regulated secretory pathway	0.189087	1
GO:0090150	establishment of protein localization to membrane	0.189087	2
GO:0021537	telencephalon development	0.189087	2
GO:0008038	neuron recognition	0.189087	1
GO:0016339	calcium-dependent cell-cell adhesion via plasma membrane cell adhesion molecules	0.189087	1
GO:0033138	positive regulation of peptidyl-serine phosphorylation	0.189087	1
GO:0140632	canonical inflammasome complex assembly	0.189087	1
GO:0141085	regulation of inflammasome-mediated signaling pathway	0.189087	1
GO:1905145	cellular response to acetylcholine	0.189087	1
GO:2000300	regulation of synaptic vesicle exocytosis	0.189087	1
GO:0051258	protein polymerization	0.189087	2
GO:0021795	cerebral cortex cell migration	0.190046	1
GO:0045197	establishment or maintenance of epithelial cell apical/basal polarity	0.190046	1
GO:0048546	digestive tract morphogenesis	0.190046	1
GO:0090279	regulation of calcium ion import	0.190046	1
GO:0002221	pattern recognition receptor signaling pathway	0.192057	2
GO:0031670	cellular response to nutrient	0.192057	1
GO:0140894	endolysosomal toll-like receptor signaling pathway	0.192057	1
GO:0006513	protein monoubiquitination	0.194597	1
GO:1905144	response to acetylcholine	0.194597	1
GO:0045165	cell fate commitment	0.197847	2
GO:0030520	estrogen receptor signaling pathway	0.197847	1

GO:0035088	establishment or maintenance of apical/basal cell polarity	0.197847	1
GO:0061245	establishment or maintenance of bipolar cell polarity	0.197847	1
GO:0098926	postsynaptic signal transduction	0.197847	1
GO:0141084	inflammasome-mediated signaling pathway	0.197847	1
GO:1903539	protein localization to postsynaptic membrane	0.197847	1
GO:0043122	regulation of canonical NF-kappaB signal transduction	0.199131	2
GO:0009395	phospholipid catabolic process	0.199131	1
GO:0062237	protein localization to postsynapse	0.199131	1
GO:0120316	sperm flagellum assembly	0.199131	1
GO:0007215	glutamate receptor signaling pathway	0.201515	1
GO:0035176	social behavior	0.201515	1
GO:0002758	innate immune response-activating signaling pathway	0.202323	2
GO:0086065	cell communication involved in cardiac conduction	0.203868	1
GO:0030865	cortical cytoskeleton organization	0.204471	1
GO:0031529	ruffle organization	0.204471	1
GO:0032147	activation of protein kinase activity	0.204471	1
GO:0050982	detection of mechanical stimulus	0.204471	1
GO:0051703	biological process involved in intraspecies interaction between organisms	0.204471	1
GO:0006584	catecholamine metabolic process	0.206181	1
GO:0009712	catechol-containing compound metabolic process	0.206181	1
GO:0035329	hippo signaling	0.206181	1
GO:0009948	anterior/posterior axis specification	0.207858	1
GO:0032413	negative regulation of ion transmembrane transporter activity	0.207858	1
GO:0086009	membrane repolarization	0.207858	1
GO:0007249	canonical NF-kappaB signal transduction	0.20953	2
GO:0048168	regulation of neuronal synaptic plasticity	0.210073	1
GO:0051047	positive regulation of secretion	0.211139	2

GO:0043113	receptor clustering	0.211139	1
GO:0086002	cardiac muscle cell action potential involved in contraction	0.211139	1
GO:0034504	protein localization to nucleus	0.211139	2
GO:0043588	skin development	0.211632	2
GO:0022029	telencephalon cell migration	0.211632	1
GO:0031663	lipopolysaccharide-mediated signaling pathway	0.211632	1
GO:0051932	synaptic transmission, GABAergic	0.211632	1
GO:0002218	activation of innate immune response	0.211632	2
GO:0048638	regulation of developmental growth	0.213124	2
GO:0003229	ventricular cardiac muscle tissue development	0.213124	1
GO:0008347	glial cell migration	0.213124	1
GO:0006790	sulfur compound metabolic process	0.213532	2
GO:0002763	positive regulation of myeloid leukocyte differentiation	0.213532	1
GO:0034142	toll-like receptor 4 signaling pathway	0.213532	1
GO:0045620	negative regulation of lymphocyte differentiation	0.213532	1
GO:0097479	synaptic vesicle localization	0.213532	1
GO:0051090	regulation of DNA-binding transcription factor activity	0.213925	2
GO:0006518	peptide metabolic process	0.213925	1
GO:0006903	vesicle targeting	0.213925	1
GO:0021885	forebrain cell migration	0.213925	1
GO:0031113	regulation of microtubule polymerization	0.213925	1
GO:0033135	regulation of peptidyl-serine phosphorylation	0.215395	1
GO:0051289	protein homotetramerization	0.215395	1
GO:0051965	positive regulation of synapse assembly	0.215395	1
GO:0048545	response to steroid hormone	0.216291	2
GO:0006605	protein targeting	0.216291	2
GO:0032922	circadian regulation of gene expression	0.216291	1
GO:0050909	sensory perception of taste	0.216291	1
GO:0035019	somatic stem cell population maintenance	0.218801	1
GO:0043525	positive regulation of neuron apoptotic process	0.220741	1
GO:0048857	neural nucleus development	0.220741	1

GO:0032956	regulation of actin cytoskeleton organization	0.222042	2
GO:0042063	gliogenesis	0.222042	2
GO:0070588	calcium ion transmembrane transport	0.222042	2
GO:0008333	endosome to lysosome transport	0.222042	1
GO:0018107	peptidyl-threonine phosphorylation	0.222042	1
GO:0030225	macrophage differentiation	0.222042	1
GO:0045600	positive regulation of fat cell differentiation	0.222042	1
GO:0021700	developmental maturation	0.222042	2
GO:0032535	regulation of cellular component size	0.222042	2
GO:0030032	lamellipodium assembly	0.222042	1
GO:0048663	neuron fate commitment	0.222042	1
GO:0051148	negative regulation of muscle cell differentiation	0.222042	1
GO:0140895	cell surface toll-like receptor signaling pathway	0.222042	1
GO:2000134	negative regulation of G1/S transition of mitotic cell cycle	0.222042	1
GO:0014015	positive regulation of gliogenesis	0.223337	1
GO:0033143	regulation of intracellular steroid hormone receptor signaling pathway	0.223337	1
GO:0071901	negative regulation of protein serine/threonine kinase activity	0.223337	1
GO:0006887	exocytosis	0.223893	2
GO:1901990	regulation of mitotic cell cycle phase transition	0.223893	2
GO:0050772	positive regulation of axonogenesis	0.224609	1
GO:0045109	intermediate filament organization	0.226931	1
GO:0050795	regulation of behavior	0.228696	1
GO:0062208	positive regulation of pattern recognition receptor signaling pathway	0.228696	1
GO:0009880	embryonic pattern specification	0.228825	1
GO:0048488	synaptic vesicle endocytosis	0.228825	1
GO:0051966	regulation of synaptic transmission, glutamatergic	0.228825	1
GO:0170035	L-amino acid catabolic process	0.228825	1
GO:0170040	proteinogenic amino acid catabolic process	0.228825	1
GO:0007585	respiratory gaseous exchange by respiratory system	0.230009	1

GO:0071277	cellular response to calcium ion	0.230009	1
GO:1903533	regulation of protein targeting	0.230009	1
GO:0044458	motile cilium assembly	0.232242	1
GO:0045685	regulation of glial cell differentiation	0.233438	1
GO:0002752	cell surface pattern recognition receptor signaling pathway	0.233438	1
GO:0018210	peptidyl-threonine modification	0.233438	1
GO:0032410	negative regulation of transporter activity	0.233438	1
GO:0045921	positive regulation of exocytosis	0.233438	1
GO:0090049	regulation of cell migration involved in sprouting angiogenesis	0.233438	1
GO:0140238	presynaptic endocytosis	0.233438	1
GO:1902807	negative regulation of cell cycle G1/S phase transition	0.233438	1
GO:0002224	toll-like receptor signaling pathway	0.234013	1
GO:0030166	proteoglycan biosynthetic process	0.234013	1
GO:0030516	regulation of axon extension	0.234013	1
GO:0043537	negative regulation of blood vessel endothelial cell migration	0.234013	1
GO:0014823	response to activity	0.235099	1
GO:0030203	glycosaminoglycan metabolic process	0.235099	1
GO:0031424	keratinization	0.235099	1
GO:0050878	regulation of body fluid levels	0.237216	2
GO:0046928	regulation of neurotransmitter secretion	0.237216	1
GO:0048013	ephrin receptor signaling pathway	0.237216	1
GO:0070509	calcium ion import	0.237216	1
GO:0086001	cardiac muscle cell action potential	0.237216	1
GO:0086003	cardiac muscle cell contraction	0.237216	1
GO:0032970	regulation of actin filament-based process	0.239856	2
GO:0030522	intracellular receptor signaling pathway	0.240203	2
GO:0001892	embryonic placenta development	0.240203	1
GO:1901379	regulation of potassium ion transmembrane transport	0.240203	1
GO:0008544	epidermis development	0.240203	2
GO:0045089	positive regulation of innate immune response	0.240203	2

GO:1902903	regulation of supramolecular fiber organization	0.240667	2
GO:0071347	cellular response to interleukin-1	0.240779	1
GO:0036465	synaptic vesicle recycling	0.242805	1
GO:0009100	glycoprotein metabolic process	0.243037	2
GO:0001701	in utero embryonic development	0.244936	2
GO:0010959	regulation of metal ion transport	0.244936	2
GO:0048708	astrocyte differentiation	0.245115	1
GO:0097581	lamellipodium organization	0.245115	1
GO:0045786	negative regulation of cell cycle	0.245115	2
GO:0032273	positive regulation of protein polymerization	0.245115	1
GO:0071230	cellular response to amino acid stimulus	0.245115	1
GO:1904063	negative regulation of cation transmembrane transport	0.245115	1
GO:2000177	regulation of neural precursor cell proliferation	0.245115	1
GO:0021766	hippocampus development	0.248486	1
GO:0140115	export across plasma membrane	0.248486	1
GO:1900182	positive regulation of protein localization to nucleus	0.248486	1
GO:0030900	forebrain development	0.248922	2
GO:0001934	positive regulation of protein phosphorylation	0.249368	2
GO:0045104	intermediate filament cytoskeleton organization	0.251799	1
GO:0002042	cell migration involved in sprouting angiogenesis	0.252121	1
GO:0045103	intermediate filament-based process	0.252121	1
GO:0061337	cardiac conduction	0.252121	1
GO:2001257	regulation of cation channel activity	0.252121	1
GO:0006022	aminoglycan metabolic process	0.252775	1
GO:1901606	alpha-amino acid catabolic process	0.252775	1
GO:1990542	mitochondrial transmembrane transport	0.252775	1
GO:0014706	striated muscle tissue development	0.252775	2
GO:0009798	axis specification	0.252775	1
GO:0034766	negative regulation of monoatomic ion transmembrane transport	0.252775	1
GO:0061387	regulation of extent of cell growth	0.252775	1

GO:0002833	positive regulation of response to biotic stimulus	0.252775	2
GO:0002220	innate immune response activating cell surface receptor signaling pathway	0.252775	1
GO:0006576	biogenic amine metabolic process	0.252775	1
GO:0031110	regulation of microtubule polymerization or depolymerization	0.252775	1
GO:0051262	protein tetramerization	0.252775	1
GO:0010596	negative regulation of endothelial cell migration	0.252775	1
GO:0034446	substrate adhesion-dependent cell spreading	0.252775	1
GO:0046785	microtubule polymerization	0.252775	1
GO:0071229	cellular response to acid chemical	0.252775	1
GO:0120162	positive regulation of cold-induced thermogenesis	0.252775	1
GO:0003002	regionalization	0.252775	2
GO:0043254	regulation of protein-containing complex assembly	0.253205	2
GO:0035082	axoneme assembly	0.254873	1
GO:0046620	regulation of organ growth	0.254873	1
GO:0051588	regulation of neurotransmitter transport	0.254873	1
GO:0043266	regulation of potassium ion transport	0.256271	1
GO:0042327	positive regulation of phosphorylation	0.256271	2
GO:0008593	regulation of Notch signaling pathway	0.257896	1
GO:0002027	regulation of heart rate	0.259134	1
GO:1905477	positive regulation of protein localization to membrane	0.259134	1
GO:0045639	positive regulation of myeloid cell differentiation	0.260358	1
GO:1990266	neutrophil migration	0.260358	1
GO:0060537	muscle tissue development	0.261568	2
GO:0006816	calcium ion transport	0.261982	2
GO:0048675	axon extension	0.26498	1
GO:0090398	cellular senescence	0.26498	1
GO:0009063	amino acid catabolic process	0.26564	1
GO:0009308	amine metabolic process	0.26564	1
GO:0035249	synaptic transmission, glutamatergic	0.26564	1

GO:0014013	regulation of gliogenesis	0.266802	1
GO:1901890	positive regulation of cell junction assembly	0.266802	1
GO:0048568	embryonic organ development	0.267091	2
GO:0007409	axonogenesis	0.26841	2
GO:0005976	polysaccharide metabolic process	0.271326	1
GO:0002065	columnar/cuboidal epithelial cell differentiation	0.271326	1
GO:0002444	myeloid leukocyte mediated immunity	0.271326	1
GO:0018958	phenol-containing compound metabolic process	0.271326	1
GO:0070252	actin-mediated cell contraction	0.271326	1
GO:0070555	response to interleukin-1	0.271326	1
GO:0051928	positive regulation of calcium ion transport	0.272499	1
GO:1901987	regulation of cell cycle phase transition	0.272499	2
GO:0007265	Ras protein signal transduction	0.274033	1
GO:0043269	regulation of monoatomic ion transport	0.274596	2
GO:0030518	nuclear receptor-mediated steroid hormone signaling pathway	0.274596	1
GO:0099072	regulation of postsynaptic membrane neurotransmitter receptor levels	0.274596	1
GO:1902106	negative regulation of leukocyte differentiation	0.276179	1
GO:0007389	pattern specification process	0.276641	2
GO:0042303	molting cycle	0.276719	1
GO:0042633	hair cycle	0.276719	1
GO:0034762	regulation of transmembrane transport	0.276895	2
GO:0050907	detection of chemical stimulus involved in sensory perception	0.278174	2
GO:0007018	microtubule-based movement	0.278555	2
GO:0045088	regulation of innate immune response	0.278935	2
GO:0021761	limbic system development	0.282386	1
GO:1903305	regulation of regulated secretory pathway	0.283909	1
GO:0006612	protein targeting to membrane	0.284379	1
GO:0030833	regulation of actin filament polymerization	0.284379	1
GO:1903707	negative regulation of hemopoiesis	0.284379	1
GO:0010562	positive regulation of phosphorus metabolic process	0.284718	2

GO:0045937	positive regulation of phosphate metabolic process	0.284718	2
GO:0006906	vesicle fusion	0.284718	1
GO:0039531	regulation of cytoplasmic pattern recognition receptor signaling pathway	0.284718	1
GO:0023061	signal release	0.284718	2
GO:0002761	regulation of myeloid leukocyte differentiation	0.284718	1
GO:0021987	cerebral cortex development	0.284718	1
GO:0043271	negative regulation of monoatomic ion transport	0.284718	1
GO:0090174	organelle membrane fusion	0.284718	1
GO:0098659	inorganic cation import across plasma membrane	0.284718	1
GO:0099587	inorganic ion import across plasma membrane	0.284718	1
GO:1903829	positive regulation of protein localization	0.28516	2
GO:0010977	negative regulation of neuron projection development	0.28516	1
GO:0043200	response to amino acid	0.28516	1
GO:0038061	non-canonical NF-kappaB signal transduction	0.288566	1
GO:0001578	microtubule bundle formation	0.289488	1
GO:0050864	regulation of B cell activation	0.289488	1
GO:0007041	lysosomal transport	0.289888	1
GO:0043401	steroid hormone receptor signaling pathway	0.289888	1
GO:0046683	response to organophosphorus	0.289888	1
GO:0006469	negative regulation of protein kinase activity	0.290791	1
GO:0051384	response to glucocorticoid	0.290791	1
GO:0030218	erythrocyte differentiation	0.294109	1
GO:0008360	regulation of cell shape	0.294469	1
GO:0030048	actin filament-based movement	0.294469	1
GO:0051592	response to calcium ion	0.294469	1
GO:0007200	phospholipase C-activating G protein-coupled receptor signaling pathway	0.294823	1
GO:0007498	mesoderm development	0.294823	1
GO:0045727	positive regulation of translation	0.294823	1
GO:0030183	B cell differentiation	0.296192	1
GO:0031109	microtubule polymerization or depolymerization	0.297041	1

GO:1904064	positive regulation of cation transmembrane transport	0.297041	1
GO:0000086	G2/M transition of mitotic cell cycle	0.297368	1
GO:0034763	negative regulation of transmembrane transport	0.297368	1
GO:0050770	regulation of axonogenesis	0.297368	1
GO:0033673	negative regulation of kinase activity	0.298711	1
GO:0009581	detection of external stimulus	0.300046	1
GO:0008064	regulation of actin polymerization or depolymerization	0.301374	1
GO:0009582	detection of abiotic stimulus	0.301665	1
GO:0045598	regulation of fat cell differentiation	0.301665	1
GO:0060048	cardiac muscle contraction	0.301665	1
GO:0007224	smoothened signaling pathway	0.302739	1
GO:0030832	regulation of actin filament length	0.302739	1
GO:0034101	erythrocyte homeostasis	0.302739	1
GO:0090090	negative regulation of canonical Wnt signaling pathway	0.302739	1
GO:0097530	granulocyte migration	0.302739	1
GO:0014074	response to purine-containing compound	0.304031	1
GO:0120161	regulation of cold-induced thermogenesis	0.305058	1
GO:0001890	placenta development	0.305058	1
GO:0030534	adult behavior	0.305058	1
GO:0034767	positive regulation of monoatomic ion transmembrane transport	0.305058	1
GO:0106106	cold-induced thermogenesis	0.305058	1
GO:1904375	regulation of protein localization to cell periphery	0.309867	1
GO:0030041	actin filament polymerization	0.310083	1
GO:0035296	regulation of tube diameter	0.310083	1
GO:0097746	blood vessel diameter maintenance	0.310083	1
GO:0031960	response to corticosteroid	0.310295	1
GO:0035150	regulation of tube size	0.310295	1
GO:0043535	regulation of blood vessel endothelial cell migration	0.310295	1
GO:0008037	cell recognition	0.311013	1

GO:0098876	vesicle-mediated transport to the plasma membrane	0.311013	1
GO:0045807	positive regulation of endocytosis	0.312235	1
GO:0007612	learning	0.312426	1
GO:0044839	cell cycle G2/M phase transition	0.312426	1
GO:0061351	neural precursor cell proliferation	0.312426	1
GO:0030307	positive regulation of cell growth	0.321252	1
GO:0070507	regulation of microtubule cytoskeleton organization	0.321252	1
GO:0051147	regulation of muscle cell differentiation	0.321252	1
GO:0051348	negative regulation of transferase activity	0.321252	1
GO:0170033	L-amino acid metabolic process	0.321252	1
GO:0170039	proteinogenic amino acid metabolic process	0.321252	1
GO:1990138	neuron projection extension	0.324093	1
GO:0048639	positive regulation of developmental growth	0.326914	1
GO:0007034	vacuolar transport	0.327525	1
GO:2000058	regulation of ubiquitin-dependent protein catabolic process	0.327525	1
GO:0051250	negative regulation of lymphocyte activation	0.328657	1
GO:0048284	organelle fusion	0.329782	1
GO:0001508	action potential	0.333121	1
GO:0050806	positive regulation of synaptic transmission	0.333121	1
GO:1902905	positive regulation of supramolecular fiber organization	0.333121	1
GO:0007519	skeletal muscle tissue development	0.334222	1
GO:0008361	regulation of cell size	0.335317	1
GO:0030178	negative regulation of Wnt signaling pathway	0.338024	1
GO:0017015	regulation of transforming growth factor beta receptor signaling pathway	0.338035	1
GO:0071248	cellular response to metal ion	0.338035	1
GO:0002262	myeloid cell homeostasis	0.338035	1
GO:0006839	mitochondrial transport	0.338035	1
GO:0043534	blood vessel endothelial cell migration	0.338035	1
GO:0141193	nuclear receptor-mediated signaling pathway	0.338035	1
GO:0001659	temperature homeostasis	0.338566	1

GO:0007189	adenylate cyclase-activating G protein-coupled receptor signaling pathway	0.338566	1
GO:1903844	regulation of cellular response to transforming growth factor beta stimulus	0.339622	1
GO:0051495	positive regulation of cytoskeleton organization	0.340672	1
GO:0008154	actin polymerization or depolymerization	0.341184	1
GO:0019827	stem cell population maintenance	0.341184	1
GO:0031345	negative regulation of cell projection organization	0.342222	1
GO:0035725	sodium ion transmembrane transport	0.343178	1
GO:1902600	proton transmembrane transport	0.343178	1
GO:0001764	neuron migration	0.343178	1
GO:0016241	regulation of macroautophagy	0.343178	1
GO:0062207	regulation of pattern recognition receptor signaling pathway	0.343178	1
GO:0097553	calcium ion transmembrane import into cytosol	0.343178	1
GO:0098727	maintenance of cell number	0.343178	1
GO:0060538	skeletal muscle organ development	0.343568	1
GO:1901991	negative regulation of mitotic cell cycle phase transition	0.343568	1
GO:0007254	JNK cascade	0.343568	1
GO:0061025	membrane fusion	0.343568	1
GO:0006941	striated muscle contraction	0.343568	1
GO:0008217	regulation of blood pressure	0.343568	1
GO:0021543	pallium development	0.343568	1
GO:0061157	mRNA destabilization	0.343568	1
GO:1902107	positive regulation of leukocyte differentiation	0.344039	1
GO:1903708	positive regulation of hemopoiesis	0.344039	1
GO:0002040	sprouting angiogenesis	0.34653	1
GO:0050779	RNA destabilization	0.347507	1
GO:0061014	positive regulation of mRNA catabolic process	0.348478	1
GO:0000302	response to reactive oxygen species	0.350932	1
GO:0046474	glycerophospholipid biosynthetic process	0.35284	1
GO:1901605	alpha-amino acid metabolic process	0.35284	1
GO:0002695	negative regulation of leukocyte activation	0.353256	1

GO:0051650	establishment of vesicle localization	0.353256	1
GO:0030073	insulin secretion	0.357126	1
GO:0098739	import across plasma membrane	0.359355	1
GO:0001933	negative regulation of protein phosphorylation	0.359355	1
GO:0045860	positive regulation of protein kinase activity	0.359355	1
GO:0051260	protein homooligomerization	0.359355	1
GO:0034764	positive regulation of transmembrane transport	0.359731	1
GO:0071383	cellular response to steroid hormone stimulus	0.359731	1
GO:0017148	negative regulation of translation	0.362072	1
GO:0008016	regulation of heart contraction	0.362967	1
GO:0007626	locomotory behavior	0.363858	1
GO:0045637	regulation of myeloid cell differentiation	0.37041	1
GO:0045216	cell-cell junction organization	0.371898	1
GO:0009612	response to mechanical stimulus	0.371898	1
GO:0009755	hormone-mediated signaling pathway	0.371898	1
GO:0033674	positive regulation of kinase activity	0.371898	1
GO:0048167	regulation of synaptic plasticity	0.371898	1
GO:0051648	vesicle localization	0.372747	1
GO:0045619	regulation of lymphocyte differentiation	0.373048	1
GO:1901654	response to ketone	0.373048	1
GO:0042326	negative regulation of phosphorylation	0.373344	1
GO:1903828	negative regulation of protein localization	0.373344	1
GO:0050866	negative regulation of cell activation	0.374179	1
GO:0007286	spermatid development	0.37911	1
GO:0048588	developmental cell growth	0.379375	1
GO:0071222	cellular response to lipopolysaccharide	0.379375	1
GO:0000819	sister chromatid segregation	0.380986	1
GO:1903313	positive regulation of mRNA metabolic process	0.380986	1
GO:0010594	regulation of endothelial cell migration	0.382581	1
GO:1902115	regulation of organelle assembly	0.382581	1
GO:0019318	hexose metabolic process	0.382752	1
GO:0097529	myeloid leukocyte migration	0.382752	1
GO:0007411	axon guidance	0.382752	1
GO:0060560	developmental growth involved in morphogenesis	0.382752	1

GO:0048515	spermatid differentiation	0.382752	1
GO:0097485	neuron projection guidance	0.382752	1
GO:1903050	regulation of proteolysis involved in protein catabolic process	0.382752	1
GO:0016054	organic acid catabolic process	0.385068	1
GO:0046395	carboxylic acid catabolic process	0.385068	1
GO:0046434	organophosphate catabolic process	0.385068	1
GO:0045017	glycerolipid biosynthetic process	0.388644	1
GO:0045930	negative regulation of mitotic cell cycle	0.388644	1
GO:0071219	cellular response to molecule of bacterial origin	0.388644	1
GO:0022604	regulation of cell morphogenesis	0.389391	1
GO:0002573	myeloid leukocyte differentiation	0.39197	1
GO:0030072	peptide hormone secretion	0.39197	1
GO:0008654	phospholipid biosynthetic process	0.39197	1
GO:0048738	cardiac muscle tissue development	0.39197	1
GO:0006814	sodium ion transport	0.39197	1
GO:0007179	transforming growth factor beta receptor signaling pathway	0.39197	1
GO:0045444	fat cell differentiation	0.39197	1
GO:0051924	regulation of calcium ion transport	0.392692	1
GO:0060047	heart contraction	0.393411	1
GO:0043523	regulation of neuron apoptotic process	0.394125	1
GO:0048863	stem cell differentiation	0.394835	1
GO:0002790	peptide secretion	0.395542	1
GO:0031669	cellular response to nutrient levels	0.395698	1
GO:1901136	carbohydrate derivative catabolic process	0.395698	1
GO:0010001	glial cell differentiation	0.395851	1
GO:0045927	positive regulation of growth	0.395851	1
GO:0005996	monosaccharide metabolic process	0.396545	1
GO:0003015	heart process	0.399152	1
GO:0051347	positive regulation of transferase activity	0.399152	1
GO:0110053	regulation of actin filament organization	0.403495	1
GO:0032886	regulation of microtubule-based process	0.404821	1
GO:1903522	regulation of blood circulation	0.404821	1

GO:0010563	negative regulation of phosphorus metabolic process	0.406133	1
GO:0045936	negative regulation of phosphate metabolic process	0.406133	1
GO:0007188	adenylate cyclase-modulating G protein-coupled receptor signaling pathway	0.406783	1
GO:0071216	cellular response to biotic stimulus	0.406877	1
GO:1901988	negative regulation of cell cycle phase transition	0.406877	1
GO:0043488	regulation of mRNA stability	0.40752	1
GO:0051259	protein complex oligomerization	0.409347	1
GO:0060828	regulation of canonical Wnt signaling pathway	0.409426	1
GO:0071695	anatomical structure maturation	0.409426	1
GO:0003018	vascular process in circulatory system	0.410054	1
GO:0098742	cell-cell adhesion via plasma-membrane adhesion molecules	0.411853	1
GO:0007611	learning or memory	0.41364	1
GO:0006520	amino acid metabolic process	0.413697	1
GO:0015833	peptide transport	0.413697	1
GO:0010721	negative regulation of cell development	0.415463	1
GO:0042113	B cell activation	0.416661	1
GO:0043487	regulation of RNA stability	0.416661	1
GO:0061013	regulation of mRNA catabolic process	0.419547	1
GO:0043542	endothelial cell migration	0.422409	1
GO:1903532	positive regulation of secretion by cell	0.426377	1
GO:0006650	glycerophospholipid metabolic process	0.430854	1
GO:0048562	embryonic organ morphogenesis	0.430854	1
GO:0043086	negative regulation of catalytic activity	0.434732	1
GO:0071560	cellular response to transforming growth factor beta stimulus	0.436318	1
GO:0030100	regulation of endocytosis	0.436318	1
GO:0051402	neuron apoptotic process	0.436318	1
GO:1904062	regulation of monoatomic cation transmembrane transport	0.437936	1
GO:0010948	negative regulation of cell cycle process	0.441137	1
GO:0071559	response to transforming growth factor beta	0.441137	1

GO:0001666	response to hypoxia	0.441641	1
GO:0046879	hormone secretion	0.445369	1
GO:0031400	negative regulation of protein modification process	0.445858	1
GO:0098813	nuclear chromosome segregation	0.446343	1
GO:0032868	response to insulin	0.447889	1
GO:0009101	glycoprotein biosynthetic process	0.448839	1
GO:0009416	response to light stimulus	0.448839	1
GO:0042886	amide transport	0.450244	1
GO:0050890	cognition	0.450244	1
GO:0090092	regulation of transmembrane receptor protein serine/threonine kinase signaling pathway	0.450244	1
GO:0036293	response to decreased oxygen levels	0.450584	1
GO:0009914	hormone transport	0.450584	1
GO:1904951	positive regulation of establishment of protein localization	0.450584	1
GO:0060070	canonical Wnt signaling pathway	0.452076	1
GO:0048872	homeostasis of number of cells	0.457514	1
GO:0002429	immune response-activating cell surface receptor signaling pathway	0.457514	1
GO:1902105	regulation of leukocyte differentiation	0.457514	1
GO:0019216	regulation of lipid metabolic process	0.459809	1
GO:0071214	cellular response to abiotic stimulus	0.459809	1
GO:0104004	cellular response to environmental stimulus	0.459809	1
GO:0034765	regulation of monoatomic ion transmembrane transport	0.46023	1
GO:0010038	response to metal ion	0.46165	1
GO:0016042	lipid catabolic process	0.463467	1
GO:0030111	regulation of Wnt signaling pathway	0.463467	1
GO:0006402	mRNA catabolic process	0.4692	1
GO:0032496	response to lipopolysaccharide	0.4692	1
GO:0070482	response to oxygen levels	0.469373	1
GO:0006936	muscle contraction	0.469373	1
GO:0010506	regulation of autophagy	0.469373	1
GO:0031589	cell-substrate adhesion	0.470725	1

GO:1990778	protein localization to cell periphery	0.4711	1
GO:0007517	muscle organ development	0.477235	1
GO:0044282	small molecule catabolic process	0.477589	1
GO:0002768	immune response-regulating cell surface receptor signaling pathway	0.478289	1
GO:0009306	protein secretion	0.478289	1
GO:0016236	macroautophagy	0.47958	1
GO:0035592	establishment of protein localization to extracellular region	0.479921	1
GO:0002237	response to molecule of bacterial origin	0.483069	1
GO:0006644	phospholipid metabolic process	0.485579	1
GO:0071692	protein localization to extracellular region	0.485579	1
GO:0090287	regulation of cellular response to growth factor stimulus	0.485898	1
GO:0030336	negative regulation of cell migration	0.48897	1
GO:0016050	vesicle organization	0.489277	1
GO:0007281	germ cell development	0.490493	1
GO:0046486	glycerolipid metabolic process	0.492287	1
GO:0050900	leukocyte migration	0.492287	1
GO:0060271	cilium assembly	0.492287	1
GO:0141091	transforming growth factor beta receptor superfamily signaling pathway	0.495266	1
GO:2000146	negative regulation of cell motility	0.498215	1
GO:1903311	regulation of mRNA metabolic process	0.498487	1
GO:0006979	response to oxidative stress	0.501396	1
GO:0001558	regulation of cell growth	0.502531	1
GO:0006631	fatty acid metabolic process	0.504528	1
GO:0006401	RNA catabolic process	0.505642	1
GO:0040013	negative regulation of locomotion	0.510183	1
GO:0044782	cilium organization	0.512968	1
GO:0007178	cell surface receptor protein serine/threonine kinase signaling pathway	0.514036	1
GO:0022412	cellular process involved in reproduction in multicellular organism	0.514469	1
GO:0042692	muscle cell differentiation	0.514469	1

GO:0030162	regulation of proteolysis	0.514682	1
GO:0007059	chromosome segregation	0.514894	1
GO:0030098	lymphocyte differentiation	0.515104	1
GO:0050911	detection of chemical stimulus involved in sensory perception of smell	0.515312	1
GO:1903706	regulation of hemopoiesis	0.517174	1
GO:0009314	response to radiation	0.52148	1
GO:0141188	nucleic acid catabolic process	0.522481	1
GO:0051223	regulation of protein transport	0.525902	1
GO:0030099	myeloid cell differentiation	0.530082	1
GO:0006869	lipid transport	0.533416	1
GO:0007015	actin filament organization	0.53514	1
GO:0007608	sensory perception of smell	0.5357	1
GO:0006935	chemotaxis	0.5357	1
GO:0003012	muscle system process	0.5357	1
GO:0051051	negative regulation of transport	0.5357	1
GO:0042330	taxis	0.535837	1
GO:0016055	Wnt signaling pathway	0.54212	1
GO:0010876	lipid localization	0.545273	1
GO:0002443	leukocyte mediated immunity	0.545379	1
GO:0007264	small GTPase-mediated signal transduction	0.547086	1
GO:0051656	establishment of organelle localization	0.547086	1
GO:0050673	epithelial cell proliferation	0.547184	1
GO:0016049	cell growth	0.549507	1
GO:0043434	response to peptide hormone	0.550334	1

- **Table S2.** Commonly downregulated GO Biological Processes (full list).

ID	Description	p.adjust	Count
GO:0006809	nitric oxide biosynthetic process	0.023026	8
GO:0034219	carbohydrate transmembrane transport	0.023026	10
GO:0045429	positive regulation of nitric oxide biosynthetic process	0.023026	6
GO:0046209	nitric oxide metabolic process	0.023026	8
GO:0008643	carbohydrate transport	0.023026	10
GO:0045428	regulation of nitric oxide biosynthetic process	0.023026	7

GO:1904407	positive regulation of nitric oxide metabolic process	0.023026	6
GO:2001057	reactive nitrogen species metabolic process	0.023026	8
GO:0080164	regulation of nitric oxide metabolic process	0.023026	7
GO:0008645	hexose transmembrane transport	0.028063	9
GO:0015749	monosaccharide transmembrane transport	0.030835	9
GO:0009612	response to mechanical stimulus	0.037504	12
GO:0072001	renal system development	0.041852	15
GO:0051180	vitamin transport	0.043824	6
GO:0043949	regulation of cAMP-mediated signaling	0.044491	4
GO:0043410	positive regulation of MAPK cascade	0.044536	18
GO:0055023	positive regulation of cardiac muscle tissue growth	0.04674	5
GO:0048639	positive regulation of developmental growth	0.04674	10
GO:1904659	D-glucose transmembrane transport	0.048637	8
GO:0008286	insulin receptor signaling pathway	0.053029	10
GO:0001822	kidney development	0.059796	14
GO:0060421	positive regulation of heart growth	0.059796	5
GO:0032869	cellular response to insulin stimulus	0.079711	12
GO:0048505	regulation of timing of cell differentiation	0.093253	3
GO:0051091	positive regulation of DNA-binding transcription factor activity	0.093253	10
GO:0033363	secretory granule organization	0.093253	6
GO:0048009	insulin-like growth factor receptor signaling pathway	0.100317	7
GO:0040034	regulation of development, heterochronic	0.100317	3
GO:0070837	dehydroascorbic acid transport	0.100317	3
GO:0003018	vascular process in circulatory system	0.107727	12
GO:0072012	glomerulus vasculature development	0.107727	4
GO:0006636	unsaturated fatty acid biosynthetic process	0.107727	5
GO:0031589	cell-substrate adhesion	0.107727	14
GO:0048010	vascular endothelial growth factor receptor signaling pathway	0.107727	7
GO:0098739	import across plasma membrane	0.107727	10
GO:0046622	positive regulation of organ growth	0.107727	5

GO:0034250	positive regulation of amide metabolic process	0.107727	4
GO:0060045	positive regulation of cardiac muscle cell proliferation	0.107727	4
GO:0061437	renal system vasculature development	0.107727	4
GO:0061440	kidney vasculature development	0.107727	4
GO:0071375	cellular response to peptide hormone stimulus	0.10781	14
GO:0060456	positive regulation of digestive system process	0.10781	3
GO:0030511	positive regulation of transforming growth factor beta receptor signaling pathway	0.110159	4
GO:1903846	positive regulation of cellular response to transforming growth factor beta stimulus	0.110159	4
GO:0046323	D-glucose import	0.113417	6
GO:0038127	ERBB signaling pathway	0.113417	9
GO:0010832	negative regulation of myotube differentiation	0.117283	3
GO:0010656	negative regulation of muscle cell apoptotic process	0.121841	5
GO:0045927	positive regulation of growth	0.123522	11
GO:0015711	organic anion transport	0.123522	16
GO:0034392	negative regulation of smooth muscle cell apoptotic process	0.128005	3
GO:0010810	regulation of cell-substrate adhesion	0.128005	10
GO:0010232	vascular transport	0.128005	6
GO:0150104	transport across blood-brain barrier	0.128005	6
GO:1905039	carboxylic acid transmembrane transport	0.141202	8
GO:0035924	cellular response to vascular endothelial growth factor stimulus	0.143362	7
GO:0007173	epidermal growth factor receptor signaling pathway	0.151326	8
GO:0043500	muscle adaptation	0.151326	7
GO:0070884	regulation of calcineurin-NFAT signaling cascade	0.151326	4
GO:1903825	organic acid transmembrane transport	0.151326	8
GO:0071318	cellular response to ATP	0.151326	3
GO:0072006	nephron development	0.152022	8
GO:0010594	regulation of endothelial cell migration	0.152022	10

GO:0106056	regulation of calcineurin-mediated signaling	0.152022	4
GO:0046942	carboxylic acid transport	0.152022	13
GO:0038084	vascular endothelial growth factor signaling pathway	0.152022	6
GO:0015849	organic acid transport	0.152022	13
GO:0043434	response to peptide hormone	0.152022	16
GO:0007411	axon guidance	0.152022	10
GO:0097485	neuron projection guidance	0.154042	10
GO:0097242	amyloid-beta clearance	0.154042	4
GO:0055021	regulation of cardiac muscle tissue growth	0.15681	5
GO:0032868	response to insulin	0.15681	12
GO:0019933	cAMP-mediated signaling	0.15681	4
GO:0051147	regulation of muscle cell differentiation	0.15681	8
GO:0044321	response to leptin	0.15681	3
GO:0030900	forebrain development	0.15681	14
GO:0042982	amyloid precursor protein metabolic process	0.15681	6
GO:0050848	regulation of calcium-mediated signaling	0.15681	6
GO:0048645	animal organ formation	0.15681	5
GO:0048638	regulation of developmental growth	0.15681	12
GO:0001654	eye development	0.15681	14
GO:0090596	sensory organ morphogenesis	0.15681	11
GO:0022604	regulation of cell morphogenesis	0.159885	10
GO:0042391	regulation of membrane potential	0.159885	15
GO:0150063	visual system development	0.159885	14
GO:1902004	positive regulation of amyloid-beta formation	0.159885	3
GO:0010611	regulation of cardiac muscle hypertrophy	0.159885	5
GO:0050435	amyloid-beta metabolic process	0.159885	5
GO:0046394	carboxylic acid biosynthetic process	0.167265	12
GO:0043542	endothelial cell migration	0.17122	11
GO:0045723	positive regulation of fatty acid biosynthetic process	0.17122	3
GO:0016053	organic acid biosynthetic process	0.17122	12
GO:0051048	negative regulation of secretion	0.17122	8
GO:0048880	sensory system development	0.17122	14
GO:0005978	glycogen biosynthetic process	0.17122	4

GO:0009250	glucan biosynthetic process	0.17122	4
GO:0014743	regulation of muscle hypertrophy	0.17122	5
GO:0060420	regulation of heart growth	0.17122	5
GO:0006835	dicarboxylic acid transport	0.17122	6
GO:0061326	renal tubule development	0.17122	6
GO:0007389	pattern specification process	0.17122	15
GO:0033173	calcineurin-NFAT signaling cascade	0.17122	4
GO:0055010	ventricular cardiac muscle tissue morphogenesis	0.17122	4
GO:0055078	sodium ion homeostasis	0.17122	4
GO:0014821	phasic smooth muscle contraction	0.172259	3
GO:0099003	vesicle-mediated transport in synapse	0.172409	10
GO:0071453	cellular response to oxygen levels	0.172409	8
GO:0071214	cellular response to abiotic stimulus	0.172409	12
GO:0104004	cellular response to environmental stimulus	0.172409	12
GO:0008038	neuron recognition	0.174048	4
GO:0048562	embryonic organ morphogenesis	0.174048	11
GO:0035994	response to muscle stretch	0.181307	3
GO:0036323	vascular endothelial growth factor receptor-1 signaling pathway	0.181307	4
GO:0060043	regulation of cardiac muscle cell proliferation	0.181307	4
GO:0099504	synaptic vesicle cycle	0.181307	9
GO:0019226	transmission of nerve impulse	0.181456	5
GO:0090100	positive regulation of transmembrane receptor protein serine/threonine kinase signaling pathway	0.18491	6
GO:0051145	smooth muscle cell differentiation	0.186892	5
GO:0051150	regulation of smooth muscle cell differentiation	0.186892	4
GO:0002534	cytokine production involved in inflammatory response	0.193514	5
GO:1900015	regulation of cytokine production involved in inflammatory response	0.193514	5
GO:0045933	positive regulation of muscle contraction	0.195595	4
GO:0072330	monocarboxylic acid biosynthetic process	0.199912	9
GO:0070482	response to oxygen levels	0.199912	12

GO:0036215	response to stem cell factor	0.199912	4
GO:0036216	cellular response to stem cell factor stimulus	0.199912	4
GO:0038109	Kit signaling pathway	0.199912	4
GO:0055123	digestive system development	0.199912	7
GO:0003151	outflow tract morphogenesis	0.199912	5
GO:0071559	response to transforming growth factor beta	0.199912	11
GO:0003007	heart morphogenesis	0.199912	10
GO:0043491	phosphatidylinositol 3-kinase/protein kinase B signal transduction	0.199912	11
GO:0001754	eye photoreceptor cell differentiation	0.199912	4
GO:0035094	response to nicotine	0.199912	4
GO:0097720	calcineurin-mediated signaling	0.199912	4
GO:0043583	ear development	0.199912	9
GO:0001837	epithelial to mesenchymal transition	0.199912	8
GO:0010592	positive regulation of lamellipodium assembly	0.199912	3
GO:0050996	positive regulation of lipid catabolic process	0.199912	3
GO:1902993	positive regulation of amyloid precursor protein catabolic process	0.199912	3
GO:0050769	positive regulation of neurogenesis	0.199912	9
GO:0038145	macrophage colony-stimulating factor signaling pathway	0.199912	4
GO:0072073	kidney epithelium development	0.199912	7
GO:0048568	embryonic organ development	0.199912	14
GO:0048013	ephrin receptor signaling pathway	0.199912	5
GO:0001773	myeloid dendritic cell activation	0.199912	3
GO:0031547	brain-derived neurotrophic factor receptor signaling pathway	0.199912	4
GO:0035790	platelet-derived growth factor receptor-alpha signaling pathway	0.199912	4
GO:0051092	positive regulation of NF-kappaB transcription factor activity	0.199912	6
GO:0043535	regulation of blood vessel endothelial cell migration	0.199912	7
GO:0098742	cell-cell adhesion via plasma-membrane adhesion molecules	0.199912	10

GO:0044650	adhesion of symbiont to host cell	0.199912	2
GO:0048840	otolith development	0.199912	2
GO:0060525	prostate glandular acinus development	0.199912	2
GO:0060872	semicircular canal development	0.199912	2
GO:0071372	cellular response to follicle-stimulating hormone stimulus	0.199912	2
GO:0071600	otic vesicle morphogenesis	0.199912	2
GO:0071803	positive regulation of podosome assembly	0.199912	2
GO:0071838	cell proliferation in bone marrow	0.199912	2
GO:1905198	manchette assembly	0.199912	2
GO:0060560	developmental growth involved in morphogenesis	0.200708	9
GO:0052547	regulation of peptidase activity	0.203719	5
GO:0018108	peptidyl-tyrosine phosphorylation	0.203719	8
GO:0007611	learning or memory	0.203719	10
GO:0010952	positive regulation of peptidase activity	0.203719	3
GO:0070168	negative regulation of biomineral tissue development	0.203719	3
GO:1901342	regulation of vasculature development	0.203719	12
GO:0018212	peptidyl-tyrosine modification	0.203719	8
GO:0034205	amyloid-beta formation	0.207626	4
GO:0050982	detection of mechanical stimulus	0.207626	4
GO:0072009	nephron epithelium development	0.213703	6
GO:0038063	collagen-activated tyrosine kinase receptor signaling pathway	0.214333	4
GO:0051090	regulation of DNA-binding transcription factor activity	0.214333	11
GO:0009615	response to virus	0.214333	13
GO:0002732	positive regulation of dendritic cell cytokine production	0.214333	2
GO:0003263	cardioblast proliferation	0.214333	2
GO:0003264	regulation of cardioblast proliferation	0.214333	2
GO:0044062	regulation of excretion	0.214333	2
GO:0060742	epithelial cell differentiation involved in prostate gland development	0.214333	2

GO:0071313	cellular response to caffeine	0.214333	2
GO:0035966	response to topologically incorrect protein	0.214333	7
GO:0048839	inner ear development	0.214333	8
GO:2001236	regulation of extrinsic apoptotic signaling pathway	0.214333	7
GO:0005979	regulation of glycogen biosynthetic process	0.214333	3
GO:0010962	regulation of glucan biosynthetic process	0.214333	3
GO:0035767	endothelial cell chemotaxis	0.214333	3
GO:0045987	positive regulation of smooth muscle contraction	0.214333	3
GO:0061448	connective tissue development	0.214333	10
GO:0055017	cardiac muscle tissue growth	0.214333	5
GO:1905897	regulation of response to endoplasmic reticulum stress	0.214333	5
GO:0030307	positive regulation of cell growth	0.214333	7
GO:0036006	cellular response to macrophage colony-stimulating factor stimulus	0.214333	4
GO:0048012	hepatocyte growth factor receptor signaling pathway	0.214333	4
GO:0048278	vesicle docking	0.214333	4
GO:0050850	positive regulation of calcium-mediated signaling	0.214333	4
GO:0055008	cardiac muscle tissue morphogenesis	0.214333	4
GO:0003002	regionalization	0.214333	13
GO:0002573	myeloid leukocyte differentiation	0.214333	9
GO:0021591	ventricular system development	0.214333	3
GO:0033198	response to ATP	0.214333	3
GO:0048566	embryonic digestive tract development	0.214333	3
GO:0060038	cardiac muscle cell proliferation	0.214333	4
GO:0007156	homophilic cell adhesion via plasma membrane adhesion molecules	0.214333	7
GO:1990845	adaptive thermogenesis	0.214333	7
GO:0002551	mast cell chemotaxis	0.214333	2
GO:0003157	endocardium development	0.214333	2
GO:0010752	regulation of cGMP-mediated signaling	0.214333	2

GO:0014831	gastro-intestinal system smooth muscle contraction	0.214333	2
GO:0021670	lateral ventricle development	0.214333	2
GO:0030157	pancreatic juice secretion	0.214333	2
GO:0035812	renal sodium excretion	0.214333	2
GO:0043950	positive regulation of cAMP-mediated signaling	0.214333	2
GO:0060379	cardiac muscle cell myoblast differentiation	0.214333	2
GO:0072070	loop of Henle development	0.214333	2
GO:1990535	neuron projection maintenance	0.214333	2
GO:0042471	ear morphogenesis	0.217242	6
GO:0038065	collagen-activated signaling pathway	0.217242	4
GO:0007589	body fluid secretion	0.21758	5
GO:0015813	L-glutamate transmembrane transport	0.219209	3
GO:0060914	heart formation	0.219209	3
GO:0010660	regulation of muscle cell apoptotic process	0.222067	5
GO:0003229	ventricular cardiac muscle tissue development	0.222067	4
GO:0035791	platelet-derived growth factor receptor-beta signaling pathway	0.222067	4
GO:0090257	regulation of muscle system process	0.222067	9
GO:0010595	positive regulation of endothelial cell migration	0.222067	6
GO:0043123	positive regulation of canonical NF-kappaB signal transduction	0.222067	8
GO:0046330	positive regulation of JNK cascade	0.222067	5
GO:0006665	sphingolipid metabolic process	0.222067	7
GO:0000038	very long-chain fatty acid metabolic process	0.222067	3
GO:0001675	acrosome assembly	0.222067	3
GO:0048333	mesodermal cell differentiation	0.222067	3
GO:0061081	positive regulation of myeloid leukocyte cytokine production involved in immune response	0.222067	3
GO:0036005	response to macrophage colony-stimulating factor	0.222067	4
GO:0043502	regulation of muscle adaptation	0.222067	5
GO:0051153	regulation of striated muscle cell differentiation	0.222067	5
GO:0008543	fibroblast growth factor receptor signaling pathway	0.222067	6

GO:0003183	mitral valve morphogenesis	0.222067	2
GO:0035810	positive regulation of urine volume	0.222067	2
GO:0042045	epithelial fluid transport	0.222067	2
GO:0044406	adhesion of symbiont to host	0.222067	2
GO:0048711	positive regulation of astrocyte differentiation	0.222067	2
GO:0071415	cellular response to purine-containing compound	0.222067	2
GO:0071801	regulation of podosome assembly	0.222067	2
GO:0006937	regulation of muscle contraction	0.223602	7
GO:0007368	determination of left/right symmetry	0.223602	6
GO:0010613	positive regulation of cardiac muscle hypertrophy	0.223602	3
GO:0019934	cGMP-mediated signaling	0.223602	3
GO:0034405	response to fluid shear stress	0.223602	3
GO:1900101	regulation of endoplasmic reticulum unfolded protein response	0.223602	3
GO:1901099	negative regulation of signal transduction in absence of ligand	0.223602	3
GO:1902745	positive regulation of lamellipodium organization	0.223602	3
GO:2001240	negative regulation of extrinsic apoptotic signaling pathway in absence of ligand	0.223602	3
GO:0032535	regulation of cellular component size	0.225723	11
GO:0001508	action potential	0.225723	7
GO:0060419	heart growth	0.225723	5
GO:0031100	animal organ regeneration	0.225723	4
GO:0052548	regulation of endopeptidase activity	0.225723	4
GO:1901224	positive regulation of non-canonical NF-kappaB signal transduction	0.225723	4
GO:0071560	cellular response to transforming growth factor beta stimulus	0.226409	10
GO:0048565	digestive tract development	0.226409	6
GO:0048661	positive regulation of smooth muscle cell proliferation	0.226409	5
GO:0072080	nephron tubule development	0.226409	5

GO:0120162	positive regulation of cold-induced thermogenesis	0.226409	5
GO:0014742	positive regulation of muscle hypertrophy	0.226409	3
GO:0030947	regulation of vascular endothelial growth factor receptor signaling pathway	0.226409	3
GO:0045773	positive regulation of axon extension	0.226409	3
GO:0045920	negative regulation of exocytosis	0.226409	3
GO:0070633	transepithelial transport	0.226409	3
GO:0032922	circadian regulation of gene expression	0.227653	4
GO:0003174	mitral valve development	0.227653	2
GO:0003222	ventricular trabecula myocardium morphogenesis	0.227653	2
GO:0030238	male sex determination	0.227653	2
GO:0032308	positive regulation of prostaglandin secretion	0.227653	2
GO:1905651	regulation of artery morphogenesis	0.227653	2
GO:0010657	muscle cell apoptotic process	0.228546	5
GO:0045862	positive regulation of proteolysis	0.230018	8
GO:0000271	polysaccharide biosynthetic process	0.230018	4
GO:0030888	regulation of B cell proliferation	0.230018	4
GO:0150116	regulation of cell-substrate junction organization	0.230018	4
GO:0034391	regulation of smooth muscle cell apoptotic process	0.230018	3
GO:0070293	renal absorption	0.230018	3
GO:0046620	regulation of organ growth	0.230018	5
GO:0150115	cell-substrate junction organization	0.230018	5
GO:0003300	cardiac muscle hypertrophy	0.230018	5
GO:0043534	blood vessel endothelial cell migration	0.230018	7
GO:0007409	axonogenesis	0.230018	13
GO:0001666	response to hypoxia	0.230018	10
GO:0045765	regulation of angiogenesis	0.230018	11
GO:0060972	left/right pattern formation	0.230018	6
GO:0003180	aortic valve morphogenesis	0.230018	3
GO:0007031	peroxisome organization	0.230018	3
GO:0019228	neuronal action potential	0.230018	3

GO:0034390	smooth muscle cell apoptotic process	0.230018	3
GO:0050974	detection of mechanical stimulus involved in sensory perception	0.230018	3
GO:0071312	cellular response to alkaloid	0.230018	3
GO:0001659	temperature homeostasis	0.230018	7
GO:0046530	photoreceptor cell differentiation	0.230018	4
GO:0031000	response to caffeine	0.230018	2
GO:0032306	regulation of prostaglandin secretion	0.230018	2
GO:0032688	negative regulation of interferon-beta production	0.230018	2
GO:0046541	saliva secretion	0.230018	2
GO:0060033	anatomical structure regression	0.230018	2
GO:0070885	negative regulation of calcineurin-NFAT signaling cascade	0.230018	2
GO:0071599	otic vesicle development	0.230018	2
GO:0072044	collecting duct development	0.230018	2
GO:0097531	mast cell migration	0.230018	2
GO:0106057	negative regulation of calcineurin-mediated signaling	0.230018	2
GO:2001138	regulation of phospholipid transport	0.230018	2
GO:2001140	positive regulation of phospholipid transport	0.230018	2
GO:0007188	adenylate cyclase-modulating G protein-coupled receptor signaling pathway	0.230018	9
GO:0007249	canonical NF-kappaB signal transduction	0.230018	10
GO:0008630	intrinsic apoptotic signaling pathway in response to DNA damage	0.230018	5
GO:0110020	regulation of actomyosin structure organization	0.230018	5
GO:0043010	camera-type eye development	0.230018	11
GO:0015718	monocarboxylic acid transport	0.230018	7
GO:0009581	detection of external stimulus	0.230018	6
GO:0003208	cardiac ventricle morphogenesis	0.230018	4
GO:0042987	amyloid precursor protein catabolic process	0.230018	4
GO:0010591	regulation of lamellipodium assembly	0.230018	3
GO:0032885	regulation of polysaccharide biosynthetic process	0.230018	3

GO:0035886	vascular associated smooth muscle cell differentiation	0.230018	3
GO:0045923	positive regulation of fatty acid metabolic process	0.230018	3
GO:0052652	cyclic purine nucleotide metabolic process	0.230018	3
GO:0070269	pyroptotic inflammatory response	0.230018	3
GO:0070873	regulation of glycogen metabolic process	0.230018	3
GO:0002532	production of molecular mediator involved in inflammatory response	0.23069	5
GO:0014897	striated muscle hypertrophy	0.23069	5
GO:0006986	response to unfolded protein	0.23069	6
GO:0009799	specification of symmetry	0.23069	6
GO:0009855	determination of bilateral symmetry	0.23069	6
GO:0048762	mesenchymal cell differentiation	0.23069	9
GO:0006935	chemotaxis	0.232257	13
GO:0030032	lamellipodium assembly	0.232705	4
GO:0051148	negative regulation of muscle cell differentiation	0.232705	4
GO:0009582	detection of abiotic stimulus	0.232989	6
GO:0042330	taxis	0.232989	13
GO:0009187	cyclic nucleotide metabolic process	0.232989	3
GO:1900016	negative regulation of cytokine production involved in inflammatory response	0.232989	3
GO:0002371	dendritic cell cytokine production	0.232989	2
GO:0002730	regulation of dendritic cell cytokine production	0.232989	2
GO:0003188	heart valve formation	0.232989	2
GO:0030903	notochord development	0.232989	2
GO:0032354	response to follicle-stimulating hormone	0.232989	2
GO:0070593	dendrite self-avoidance	0.232989	2
GO:0014896	muscle hypertrophy	0.232989	5
GO:0051851	host-mediated perturbation of symbiont process	0.232989	5
GO:0015800	acidic amino acid transport	0.232989	4
GO:0032835	glomerulus development	0.232989	4
GO:0060415	muscle tissue morphogenesis	0.232989	4
GO:0060326	cell chemotaxis	0.235151	10
GO:0006939	smooth muscle contraction	0.237675	5

GO:0042472	inner ear morphogenesis	0.237675	5
GO:0006898	receptor-mediated endocytosis	0.237675	9
GO:0021537	telencephalon development	0.237675	9
GO:0071260	cellular response to mechanical stimulus	0.237675	4
GO:0046427	positive regulation of receptor signaling pathway via JAK-STAT	0.237675	3
GO:0051154	negative regulation of striated muscle cell differentiation	0.237675	3
GO:0014706	striated muscle tissue development	0.237675	12
GO:0014074	response to purine-containing compound	0.238985	6
GO:0120161	regulation of cold-induced thermogenesis	0.240916	6
GO:0050890	cognition	0.240916	10
GO:0097191	extrinsic apoptotic signaling pathway	0.240916	8
GO:0002090	regulation of receptor internalization	0.240916	4
GO:0003281	ventricular septum development	0.240916	4
GO:0071496	cellular response to external stimulus	0.240916	4
GO:0022600	digestive system process	0.240916	5
GO:2001243	negative regulation of intrinsic apoptotic signaling pathway	0.240916	5
GO:0036293	response to decreased oxygen levels	0.240916	10
GO:0044058	regulation of digestive system process	0.240916	3
GO:0016075	rRNA catabolic process	0.240916	2
GO:0045725	positive regulation of glycogen biosynthetic process	0.240916	2
GO:0046068	cGMP metabolic process	0.240916	2
GO:0051382	kinetochore assembly	0.240916	2
GO:0071468	cellular response to acidic pH	0.240916	2
GO:0002687	positive regulation of leukocyte migration	0.240916	6
GO:0008584	male gonad development	0.240916	6
GO:0106106	cold-induced thermogenesis	0.240916	6
GO:0050818	regulation of coagulation	0.244264	4
GO:1904018	positive regulation of vasculature development	0.244264	7
GO:0120034	positive regulation of plasma membrane bounded cell projection assembly	0.244264	5

GO:0046546	development of primary male sexual characteristics	0.244264	6
GO:0051962	positive regulation of nervous system development	0.244264	9
GO:0042692	muscle cell differentiation	0.244264	12
GO:0050954	sensory perception of mechanical stimulus	0.247667	7
GO:1903531	negative regulation of secretion by cell	0.248836	6
GO:0051938	L-glutamate import	0.248836	3
GO:0033559	unsaturated fatty acid metabolic process	0.253007	5
GO:0090287	regulation of cellular response to growth factor stimulus	0.253007	11
GO:0010827	regulation of D-glucose transmembrane transport	0.253007	4
GO:0061045	negative regulation of wound healing	0.253007	4
GO:0010002	cardioblast differentiation	0.253007	2
GO:0032148	activation of protein kinase B activity	0.253007	2
GO:0043217	myelin maintenance	0.253007	2
GO:0055119	relaxation of cardiac muscle	0.253007	2
GO:0061323	cell proliferation involved in heart morphogenesis	0.253007	2
GO:0071371	cellular response to gonadotropin stimulus	0.253007	2
GO:0098712	L-glutamate import across plasma membrane	0.253007	2
GO:2000136	regulation of cell proliferation involved in heart morphogenesis	0.253007	2
GO:0035296	regulation of tube diameter	0.253007	6
GO:0071456	cellular response to hypoxia	0.253007	6
GO:0097746	blood vessel diameter maintenance	0.253007	6
GO:0003176	aortic valve development	0.253302	3
GO:0060562	epithelial tube morphogenesis	0.253676	10
GO:0060840	artery development	0.25452	5
GO:0035150	regulation of tube size	0.25737	6
GO:0140056	organelle localization by membrane tethering	0.258437	4
GO:0006766	vitamin metabolic process	0.260861	5
GO:0050878	regulation of body fluid levels	0.26129	11
GO:0001504	neurotransmitter uptake	0.262237	3

GO:0010463	mesenchymal cell proliferation	0.262237	3
GO:2001239	regulation of extrinsic apoptotic signaling pathway in absence of ligand	0.262237	3
GO:0016050	vesicle organization	0.262938	11
GO:0002028	regulation of sodium ion transport	0.264438	4
GO:0060485	mesenchyme development	0.266282	10
GO:0030949	positive regulation of vascular endothelial growth factor receptor signaling pathway	0.266282	2
GO:0032310	prostaglandin secretion	0.266282	2
GO:0060263	regulation of respiratory burst	0.266282	2
GO:0060977	coronary vasculature morphogenesis	0.266282	2
GO:2000811	negative regulation of anoikis	0.266282	2
GO:0007281	germ cell development	0.266282	11
GO:0007612	learning	0.267406	6
GO:0048644	muscle organ morphogenesis	0.267406	4
GO:0010830	regulation of myotube differentiation	0.267406	3
GO:0030890	positive regulation of B cell proliferation	0.267406	3
GO:0032941	secretion by tissue	0.267406	3
GO:0071548	response to dexamethasone	0.267406	3
GO:0033002	muscle cell proliferation	0.274029	8
GO:0120032	regulation of plasma membrane bounded cell projection assembly	0.274029	7
GO:0032309	icosanoid secretion	0.274029	3
GO:0032881	regulation of polysaccharide metabolic process	0.274029	3
GO:0061383	trabecula morphogenesis	0.274029	3
GO:0015908	fatty acid transport	0.274029	5
GO:0060537	muscle tissue development	0.274029	12
GO:1902903	regulation of supramolecular fiber organization	0.274029	11
GO:0036294	cellular response to decreased oxygen levels	0.274029	6
GO:0006816	calcium ion transport	0.274029	12
GO:0014823	response to activity	0.274029	4
GO:0019233	sensory perception of pain	0.274029	4
GO:0048844	artery morphogenesis	0.274029	4
GO:0010950	positive regulation of endopeptidase activity	0.274029	2
GO:0032305	positive regulation of icosanoid secretion	0.274029	2

GO:0043011	myeloid dendritic cell differentiation	0.274029	2
GO:0045472	response to ether	0.274029	2
GO:0060340	positive regulation of type I interferon-mediated signaling pathway	0.274029	2
GO:0070875	positive regulation of glycogen metabolic process	0.274029	2
GO:0070886	positive regulation of calcineurin-NFAT signaling cascade	0.274029	2
GO:0097254	renal tubular secretion	0.274029	2
GO:0106058	positive regulation of calcineurin-mediated signaling	0.274029	2
GO:1902001	fatty acid transmembrane transport	0.274029	2
GO:0032956	regulation of actin cytoskeleton organization	0.274029	10
GO:0043122	regulation of canonical NF-kappaB signal transduction	0.274196	9
GO:0071482	cellular response to light stimulus	0.274196	5
GO:0060491	regulation of cell projection assembly	0.276851	7
GO:0048546	digestive tract morphogenesis	0.277292	3
GO:1902003	regulation of amyloid-beta formation	0.277292	3
GO:1904894	positive regulation of receptor signaling pathway via STAT	0.277292	3
GO:1905314	semi-lunar valve development	0.277292	3
GO:0070588	calcium ion transmembrane transport	0.278203	10
GO:0035821	modulation of process of another organism	0.278203	5
GO:0042060	wound healing	0.278284	12
GO:0044344	cellular response to fibroblast growth factor stimulus	0.278284	6
GO:0048863	stem cell differentiation	0.279569	8
GO:0051017	actin filament bundle assembly	0.279569	6
GO:0006970	response to osmotic stress	0.279569	4
GO:0007193	adenylate cyclase-inhibiting G protein-coupled receptor signaling pathway	0.279569	4
GO:0014855	striated muscle cell proliferation	0.279569	4
GO:0048041	focal adhesion assembly	0.279569	4
GO:0051149	positive regulation of muscle cell differentiation	0.279569	4

GO:0030195	negative regulation of blood coagulation	0.279569	3
GO:0061082	myeloid leukocyte cytokine production	0.279569	3
GO:1903573	negative regulation of response to endoplasmic reticulum stress	0.279569	3
GO:0009190	cyclic nucleotide biosynthetic process	0.279569	2
GO:0035461	vitamin transmembrane transport	0.279569	2
GO:0036498	IRE1-mediated unfolded protein response	0.279569	2
GO:0042359	vitamin D metabolic process	0.279569	2
GO:0042474	middle ear morphogenesis	0.279569	2
GO:0050849	negative regulation of calcium-mediated signaling	0.279569	2
GO:0051383	kinetochore organization	0.279569	2
GO:0060973	cell migration involved in heart development	0.279569	2
GO:0071800	podosome assembly	0.279569	2
GO:1903975	regulation of glial cell migration	0.279569	2
GO:2000273	positive regulation of signaling receptor activity	0.279569	2
GO:0021761	limbic system development	0.28039	5
GO:0034248	regulation of amide metabolic process	0.283274	4
GO:0070665	positive regulation of leukocyte proliferation	0.284858	6
GO:0014911	positive regulation of smooth muscle cell migration	0.287962	3
GO:1900047	negative regulation of hemostasis	0.287962	3
GO:1990138	neuron projection extension	0.288182	6
GO:0005977	glycogen metabolic process	0.288182	4
GO:0045732	positive regulation of protein catabolic process	0.288182	7
GO:0006633	fatty acid biosynthetic process	0.288182	6
GO:0061572	actin filament bundle organization	0.288182	6
GO:0006883	intracellular sodium ion homeostasis	0.288182	2
GO:0007530	sex determination	0.288182	2
GO:0015919	peroxisomal membrane transport	0.288182	2
GO:0032303	regulation of icosanoid secretion	0.288182	2
GO:0035809	regulation of urine volume	0.288182	2
GO:0036376	sodium ion export across plasma membrane	0.288182	2
GO:0043574	peroxisomal transport	0.288182	2
GO:0044241	lipid digestion	0.288182	2

GO:0060713	labyrinthine layer morphogenesis	0.288182	2
GO:0071498	cellular response to fluid shear stress	0.288182	2
GO:0072234	metanephric nephron tubule development	0.288182	2
GO:0098801	regulation of renal system process	0.288182	2
GO:1902307	positive regulation of sodium ion transmembrane transport	0.288182	2
GO:0030968	endoplasmic reticulum unfolded protein response	0.288182	4
GO:0032370	positive regulation of lipid transport	0.288182	4
GO:0045332	phospholipid translocation	0.288182	3
GO:1902743	regulation of lamellipodium organization	0.288182	3
GO:0006936	muscle contraction	0.288182	10
GO:0048259	regulation of receptor-mediated endocytosis	0.288182	5
GO:0072089	stem cell proliferation	0.288182	5
GO:0071805	potassium ion transmembrane transport	0.290116	7
GO:0001558	regulation of cell growth	0.290446	11
GO:0031346	positive regulation of cell projection organization	0.290464	10
GO:0003231	cardiac ventricle development	0.29315	5
GO:0007613	memory	0.29315	5
GO:0050920	regulation of chemotaxis	0.29315	7
GO:0022406	membrane docking	0.29315	4
GO:0044042	glucan metabolic process	0.29315	4
GO:1904707	positive regulation of vascular associated smooth muscle cell proliferation	0.296459	3
GO:0046777	protein autophosphorylation	0.297604	6
GO:0071774	response to fibroblast growth factor	0.297604	6
GO:0048608	reproductive structure development	0.300479	9
GO:0061515	myeloid cell development	0.300479	4
GO:0009651	response to salt stress	0.300572	2
GO:0016338	calcium-independent cell-cell adhesion via plasma membrane cell-adhesion molecules	0.300572	2
GO:0046058	cAMP metabolic process	0.300572	2
GO:1902074	response to salt	0.300572	2
GO:0048660	regulation of smooth muscle cell proliferation	0.300572	6

GO:0110053	regulation of actin filament organization	0.303184	8
GO:0042304	regulation of fatty acid biosynthetic process	0.303184	3
GO:0050819	negative regulation of coagulation	0.303184	3
GO:0003012	muscle system process	0.303184	12
GO:0034644	cellular response to UV	0.304423	4
GO:0097581	lamellipodium organization	0.304423	4
GO:0006643	membrane lipid metabolic process	0.306902	7
GO:0046661	male sex differentiation	0.309987	6
GO:0061458	reproductive system development	0.311819	9
GO:0038061	non-canonical NF-kappaB signal transduction	0.311819	5
GO:0045834	positive regulation of lipid metabolic process	0.311819	5
GO:0035176	social behavior	0.311819	3
GO:0070371	ERK1 and ERK2 cascade	0.311819	9
GO:0048167	regulation of synaptic plasticity	0.311819	7
GO:0007517	muscle organ development	0.311819	10
GO:0002320	lymphoid progenitor cell differentiation	0.311819	2
GO:0008156	negative regulation of DNA replication	0.311819	2
GO:0014059	regulation of dopamine secretion	0.311819	2
GO:0046597	host-mediated suppression of symbiont invasion	0.311819	2
GO:0051131	chaperone-mediated protein complex assembly	0.311819	2
GO:2000193	positive regulation of fatty acid transport	0.311819	2
GO:2001026	regulation of endothelial cell chemotaxis	0.311819	2
GO:0001656	metanephros development	0.314345	4
GO:0042100	B cell proliferation	0.314345	4
GO:0048659	smooth muscle cell proliferation	0.314345	6
GO:0001894	tissue homeostasis	0.315065	8
GO:0060249	anatomical structure homeostasis	0.315065	8
GO:0051607	defense response to virus	0.315065	9
GO:0046854	phosphatidylinositol phosphate biosynthetic process	0.315065	3
GO:0048260	positive regulation of receptor-mediated endocytosis	0.315065	3
GO:1904738	vascular associated smooth muscle cell migration	0.315065	3

GO:2000648	positive regulation of stem cell proliferation	0.315065	3
GO:0046683	response to organophosphorus	0.315729	5
GO:0048706	embryonic skeletal system development	0.315729	5
GO:0008361	regulation of cell size	0.315729	6
GO:0050767	regulation of neurogenesis	0.316677	10
GO:0070663	regulation of leukocyte proliferation	0.316677	8
GO:0042476	odontogenesis	0.317886	5
GO:0051384	response to glucocorticoid	0.317886	5
GO:0030865	cortical cytoskeleton organization	0.317886	3
GO:0034204	lipid translocation	0.317886	3
GO:0051703	biological process involved in intraspecies interaction between organisms	0.317886	3
GO:0071470	cellular response to osmotic stress	0.317886	3
GO:1902991	regulation of amyloid precursor protein catabolic process	0.317886	3
GO:0032693	negative regulation of interleukin-10 production	0.317886	2
GO:0034453	microtubule anchoring	0.317886	2
GO:0048169	regulation of long-term neuronal synaptic plasticity	0.317886	2
GO:0051152	positive regulation of smooth muscle cell differentiation	0.317886	2
GO:0060907	positive regulation of macrophage cytokine production	0.317886	2
GO:0072170	metanephric tubule development	0.317886	2
GO:0072243	metanephric nephron epithelium development	0.317886	2
GO:1903306	negative regulation of regulated secretory pathway	0.317886	2
GO:1905063	regulation of vascular associated smooth muscle cell differentiation	0.317886	2
GO:0051492	regulation of stress fiber assembly	0.318281	4
GO:0045055	regulated exocytosis	0.323017	7
GO:0017015	regulation of transforming growth factor beta receptor signaling pathway	0.323951	6
GO:0031214	biomineral tissue development	0.323951	6
GO:0071248	cellular response to metal ion	0.323951	6

GO:0007044	cell-substrate junction assembly	0.324544	4
GO:0045104	intermediate filament cytoskeleton organization	0.324544	4
GO:0072091	regulation of stem cell proliferation	0.324544	4
GO:1903052	positive regulation of proteolysis involved in protein catabolic process	0.325251	5
GO:2001238	positive regulation of extrinsic apoptotic signaling pathway	0.325442	3
GO:0022412	cellular process involved in reproduction in multicellular organism	0.327646	11
GO:0030162	regulation of proteolysis	0.328177	11
GO:0008360	regulation of cell shape	0.328177	5
GO:0046328	regulation of JNK cascade	0.328177	5
GO:0015837	amine transport	0.328177	4
GO:0045103	intermediate filament-based process	0.328177	4
GO:0046889	positive regulation of lipid biosynthetic process	0.328177	4
GO:0003148	outflow tract septum morphogenesis	0.328177	2
GO:0039694	viral RNA genome replication	0.328177	2
GO:0060740	prostate gland epithelium morphogenesis	0.328177	2
GO:0090023	positive regulation of neutrophil chemotaxis	0.328177	2
GO:0099149	regulation of postsynaptic neurotransmitter receptor internalization	0.328177	2
GO:2000209	regulation of anoikis	0.328177	2
GO:0007286	spermatid development	0.329431	7
GO:0090092	regulation of transmembrane receptor protein serine/threonine kinase signaling pathway	0.329643	9
GO:0061462	protein localization to lysosome	0.330372	3
GO:0061041	regulation of wound healing	0.331811	5
GO:1903844	regulation of cellular response to transforming growth factor beta stimulus	0.332351	6
GO:0097193	intrinsic apoptotic signaling pathway	0.332536	9
GO:0002275	myeloid cell activation involved in immune response	0.332746	4
GO:0002702	positive regulation of production of molecular mediator of immune response	0.336645	5
GO:0048705	skeletal system morphogenesis	0.336645	7

GO:0010718	positive regulation of epithelial to mesenchymal transition	0.336645	3
GO:0032608	interferon-beta production	0.336645	3
GO:0032648	regulation of interferon-beta production	0.336645	3
GO:0046324	regulation of D-glucose import	0.336645	3
GO:0060998	regulation of dendritic spine development	0.336645	3
GO:0070542	response to fatty acid	0.336645	3
GO:0042542	response to hydrogen peroxide	0.336645	4
GO:0060993	kidney morphogenesis	0.336645	4
GO:0050821	protein stabilization	0.336645	7
GO:0034976	response to endoplasmic reticulum stress	0.336645	8
GO:0002053	positive regulation of mesenchymal cell proliferation	0.336645	2
GO:0003181	atrioventricular valve morphogenesis	0.336645	2
GO:0050926	regulation of positive chemotaxis	0.336645	2
GO:0060669	embryonic placenta morphogenesis	0.336645	2
GO:0071467	cellular response to pH	0.336645	2
GO:1903859	regulation of dendrite extension	0.336645	2
GO:1904754	positive regulation of vascular associated smooth muscle cell migration	0.336645	2
GO:0019722	calcium-mediated signaling	0.340026	7
GO:0042327	positive regulation of phosphorylation	0.341012	11
GO:0021675	nerve development	0.341016	4
GO:0001961	positive regulation of cytokine-mediated signaling pathway	0.341318	3
GO:0045604	regulation of epidermal cell differentiation	0.341318	3
GO:0046456	icosanoid biosynthetic process	0.341318	3
GO:0060612	adipose tissue development	0.341318	3
GO:2001242	regulation of intrinsic apoptotic signaling pathway	0.344691	6
GO:0007586	digestion	0.345943	5
GO:0031623	receptor internalization	0.345943	5
GO:0014909	smooth muscle cell migration	0.345943	4
GO:1903035	negative regulation of response to wounding	0.345943	4
GO:0097529	myeloid leukocyte migration	0.345943	7

GO:0010562	positive regulation of phosphorus metabolic process	0.345984	12
GO:0045937	positive regulation of phosphate metabolic process	0.345984	12
GO:0048736	appendage development	0.345984	6
GO:0060173	limb development	0.345984	6
GO:0003179	heart valve morphogenesis	0.345984	3
GO:0032731	positive regulation of interleukin-1 beta production	0.345984	3
GO:0097035	regulation of membrane lipid distribution	0.345984	3
GO:0010882	regulation of cardiac muscle contraction by calcium ion signaling	0.345984	2
GO:0034698	response to gonadotropin	0.345984	2
GO:0060512	prostate gland morphogenesis	0.345984	2
GO:0071624	positive regulation of granulocyte chemotaxis	0.345984	2
GO:0006112	energy reserve metabolic process	0.348284	4
GO:0034620	cellular response to unfolded protein	0.348284	4
GO:2001237	negative regulation of extrinsic apoptotic signaling pathway	0.348284	4
GO:0048515	spermatid differentiation	0.349783	7
GO:1903050	regulation of proteolysis involved in protein catabolic process	0.349783	7
GO:0050921	positive regulation of chemotaxis	0.352471	5
GO:0045766	positive regulation of angiogenesis	0.352471	6
GO:0007160	cell-matrix adhesion	0.352471	7
GO:0008406	gonad development	0.352471	7
GO:0006940	regulation of smooth muscle contraction	0.352471	3
GO:0051893	regulation of focal adhesion assembly	0.352471	3
GO:0090109	regulation of cell-substrate junction assembly	0.352471	3
GO:0023061	signal release	0.352471	12
GO:0070167	regulation of biomineral tissue development	0.352471	4
GO:0032970	regulation of actin filament-based process	0.354107	10
GO:0007254	JNK cascade	0.354599	6
GO:0003158	endothelium development	0.354599	5
GO:0007596	blood coagulation	0.354599	7

GO:0002092	positive regulation of receptor internalization	0.354599	2
GO:0003094	glomerular filtration	0.354599	2
GO:0010447	response to acidic pH	0.354599	2
GO:0014046	dopamine secretion	0.354599	2
GO:0043392	negative regulation of DNA binding	0.354599	2
GO:0060384	innervation	0.354599	2
GO:0072207	metanephric epithelium development	0.354599	2
GO:0006767	water-soluble vitamin metabolic process	0.358234	3
GO:0035148	tube formation	0.358234	5
GO:0010720	positive regulation of cell development	0.35865	11
GO:0050670	regulation of lymphocyte proliferation	0.361334	7
GO:2001234	negative regulation of apoptotic signaling pathway	0.361334	7
GO:0010975	regulation of neuron projection development	0.361513	11
GO:0016197	endosomal transport	0.362525	8
GO:0070661	leukocyte proliferation	0.362718	9
GO:0016331	morphogenesis of embryonic epithelium	0.362718	5
GO:0002931	response to ischemia	0.364568	3
GO:0071715	icosanoid transport	0.364568	3
GO:0043549	regulation of kinase activity	0.364568	10
GO:0060078	regulation of postsynaptic membrane potential	0.364568	5
GO:0003171	atrioventricular valve development	0.364568	2
GO:0007588	excretion	0.364568	2
GO:0015732	prostaglandin transport	0.364568	2
GO:0034508	centromere complex assembly	0.364568	2
GO:1900017	positive regulation of cytokine production involved in inflammatory response	0.364568	2
GO:1902230	negative regulation of intrinsic apoptotic signaling pathway in response to DNA damage	0.364568	2
GO:0006813	potassium ion transport	0.364568	7
GO:0045137	development of primary sexual characteristics	0.364568	7
GO:0048008	platelet-derived growth factor receptor signaling pathway	0.364568	4
GO:2000060	positive regulation of ubiquitin-dependent protein catabolic process	0.364568	4

GO:0045165	cell fate commitment	0.364831	8
GO:0141091	transforming growth factor beta receptor superfamily signaling pathway	0.368416	10
GO:2001233	regulation of apoptotic signaling pathway	0.368416	10
GO:0050817	coagulation	0.368416	7
GO:0010812	negative regulation of cell-substrate adhesion	0.36879	3
GO:0032613	interleukin-10 production	0.36879	3
GO:0032653	regulation of interleukin-10 production	0.36879	3
GO:0097192	extrinsic apoptotic signaling pathway in absence of ligand	0.36879	3
GO:0070374	positive regulation of ERK1 and ERK2 cascade	0.36879	6
GO:0032231	regulation of actin filament bundle assembly	0.36879	4
GO:1901222	regulation of non-canonical NF-kappaB signal transduction	0.36879	4
GO:0042063	gliogenesis	0.369315	9
GO:0051960	regulation of nervous system development	0.37205	11
GO:0035107	appendage morphogenesis	0.37205	5
GO:0035108	limb morphogenesis	0.37205	5
GO:0007599	hemostasis	0.372403	7
GO:0032944	regulation of mononuclear cell proliferation	0.372403	7
GO:0030099	myeloid cell differentiation	0.372403	11
GO:0003338	metanephros morphogenesis	0.372403	2
GO:0030878	thyroid gland development	0.372403	2
GO:0031629	synaptic vesicle fusion to presynaptic active zone membrane	0.372403	2
GO:0051491	positive regulation of filopodium assembly	0.372403	2
GO:0060037	pharyngeal system development	0.372403	2
GO:0015909	long-chain fatty acid transport	0.372403	3
GO:0035904	aorta development	0.372403	3
GO:0050994	regulation of lipid catabolic process	0.372403	3
GO:0070301	cellular response to hydrogen peroxide	0.372403	3
GO:0071300	cellular response to retinoic acid	0.372403	3
GO:0043405	regulation of MAP kinase activity	0.378789	4
GO:0048709	oligodendrocyte differentiation	0.378789	4
GO:0060759	regulation of response to cytokine stimulus	0.378811	6

GO:0032414	positive regulation of ion transmembrane transporter activity	0.381363	3
GO:0035019	somatic stem cell population maintenance	0.381363	3
GO:0038034	signal transduction in absence of ligand	0.381363	3
GO:0045682	regulation of epidermis development	0.381363	3
GO:0060760	positive regulation of response to cytokine stimulus	0.381363	3
GO:0002274	myeloid leukocyte activation	0.381831	7
GO:0048738	cardiac muscle tissue development	0.381831	7
GO:0032760	positive regulation of tumor necrosis factor production	0.383401	4
GO:0048675	axon extension	0.383401	4
GO:0006622	protein targeting to lysosome	0.383401	2
GO:0097205	renal filtration	0.383401	2
GO:0099500	vesicle fusion to plasma membrane	0.383401	2
GO:0006869	lipid transport	0.383401	11
GO:0006814	sodium ion transport	0.383928	7
GO:0007179	transforming growth factor beta receptor signaling pathway	0.383928	7
GO:0045667	regulation of osteoblast differentiation	0.38705	5
GO:0050730	regulation of peptidyl-tyrosine phosphorylation	0.38705	5
GO:0062013	positive regulation of small molecule metabolic process	0.38705	5
GO:0030193	regulation of blood coagulation	0.387224	3
GO:0003279	cardiac septum development	0.387763	4
GO:0007015	actin filament organization	0.39262	11
GO:0060047	heart contraction	0.39262	7
GO:0031960	response to corticosteroid	0.39262	5
GO:0031334	positive regulation of protein-containing complex assembly	0.394499	6
GO:0006687	glycosphingolipid metabolic process	0.395617	3
GO:0046888	negative regulation of hormone secretion	0.395617	3
GO:0060219	camera-type eye photoreceptor cell differentiation	0.395617	2
GO:0061097	regulation of protein tyrosine kinase activity	0.395617	2

GO:0061384	heart trabecula morphogenesis	0.395617	2
GO:0006887	exocytosis	0.395617	9
GO:0008037	cell recognition	0.395617	5
GO:0042552	myelination	0.395617	5
GO:0098876	vesicle-mediated transport to the plasma membrane	0.395617	5
GO:1905954	positive regulation of lipid localization	0.400343	4
GO:0048754	branching morphogenesis of an epithelial tube	0.401881	5
GO:0007623	circadian rhythm	0.401881	6
GO:0006631	fatty acid metabolic process	0.401881	10
GO:0051051	negative regulation of transport	0.401881	11
GO:0043406	positive regulation of MAP kinase activity	0.401881	3
GO:1900046	regulation of hemostasis	0.401881	3
GO:0001934	positive regulation of protein phosphorylation	0.401881	10
GO:0030038	contractile actin filament bundle assembly	0.401881	4
GO:0030148	sphingolipid biosynthetic process	0.401881	4
GO:0043149	stress fiber assembly	0.401881	4
GO:0045445	myoblast differentiation	0.401881	4
GO:0071902	positive regulation of protein serine/threonine kinase activity	0.401881	4
GO:1903557	positive regulation of tumor necrosis factor superfamily cytokine production	0.401881	4
GO:0007272	ensheathment of neurons	0.401881	5
GO:0008366	axon ensheathment	0.401881	5
GO:0010464	regulation of mesenchymal cell proliferation	0.401881	2
GO:0031069	hair follicle morphogenesis	0.401881	2
GO:0034067	protein localization to Golgi apparatus	0.401881	2
GO:0045671	negative regulation of osteoclast differentiation	0.401881	2
GO:0048710	regulation of astrocyte differentiation	0.401881	2
GO:0098884	postsynaptic neurotransmitter receptor internalization	0.401881	2
GO:0140239	postsynaptic endocytosis	0.401881	2
GO:2000191	regulation of fatty acid transport	0.401881	2
GO:0010639	negative regulation of organelle organization	0.402057	9
GO:0048663	neuron fate commitment	0.405153	3

GO:0050918	positive chemotaxis	0.405153	3
GO:0005976	polysaccharide metabolic process	0.405196	4
GO:0014812	muscle cell migration	0.405196	4
GO:1901800	positive regulation of proteasomal protein catabolic process	0.405196	4
GO:0051146	striated muscle cell differentiation	0.406468	8
GO:0045860	positive regulation of protein kinase activity	0.408089	6
GO:0051216	cartilage development	0.408089	6
GO:0050731	positive regulation of peptidyl-tyrosine phosphorylation	0.411845	4
GO:0014015	positive regulation of gliogenesis	0.411845	3
GO:0006836	neurotransmitter transport	0.411845	6
GO:0034764	positive regulation of transmembrane transport	0.411845	6
GO:0001556	oocyte maturation	0.411845	2
GO:0006884	cell volume homeostasis	0.411845	2
GO:0010667	negative regulation of cardiac muscle cell apoptotic process	0.411845	2
GO:0051353	positive regulation of oxidoreductase activity	0.411845	2
GO:0090022	regulation of neutrophil chemotaxis	0.411845	2
GO:0090075	relaxation of muscle	0.411845	2
GO:2000406	positive regulation of T cell migration	0.411845	2
GO:0008277	regulation of G protein-coupled receptor signaling pathway	0.413272	5
GO:0003015	heart process	0.418137	7
GO:0006635	fatty acid beta-oxidation	0.418137	3
GO:0032732	positive regulation of interleukin-1 production	0.418137	3
GO:0050772	positive regulation of axonogenesis	0.418137	3
GO:0060411	cardiac septum morphogenesis	0.418137	3
GO:1902475	L-alpha-amino acid transmembrane transport	0.418137	3
GO:1903051	negative regulation of proteolysis involved in protein catabolic process	0.418137	3
GO:0035967	cellular response to topologically incorrect protein	0.4204	4
GO:0099565	chemical synaptic transmission, postsynaptic	0.4204	4
GO:0045859	regulation of protein kinase activity	0.4204	9

GO:0045861	negative regulation of proteolysis	0.4204	5
GO:0008016	regulation of heart contraction	0.4204	6
GO:0001975	response to amphetamine	0.4204	2
GO:0002067	glandular epithelial cell differentiation	0.4204	2
GO:0007617	mating behavior	0.4204	2
GO:0010765	positive regulation of sodium ion transport	0.4204	2
GO:0043276	anoikis	0.4204	2
GO:0048499	synaptic vesicle membrane organization	0.4204	2
GO:0060317	cardiac epithelial to mesenchymal transition	0.4204	2
GO:0140058	neuron projection arborization	0.4204	2
GO:1902624	positive regulation of neutrophil migration	0.4204	2
GO:0034762	regulation of transmembrane transport	0.4204	11
GO:0003170	heart valve development	0.421291	3
GO:0032088	negative regulation of NF-kappaB transcription factor activity	0.421291	3
GO:0032411	positive regulation of transporter activity	0.421291	3
GO:0045109	intermediate filament organization	0.421291	3
GO:0002768	immune response-regulating cell surface receptor signaling pathway	0.430307	9
GO:0002366	leukocyte activation involved in immune response	0.43035	8
GO:1902904	negative regulation of supramolecular fiber organization	0.43035	5
GO:0001707	mesoderm formation	0.43035	3
GO:0007178	cell surface receptor protein serine/threonine kinase signaling pathway	0.43035	10
GO:0010876	lipid localization	0.43035	11
GO:0010092	specification of animal organ identity	0.43035	2
GO:0010614	negative regulation of cardiac muscle hypertrophy	0.43035	2
GO:0016242	negative regulation of macroautophagy	0.43035	2
GO:0042462	eye photoreceptor cell development	0.43035	2
GO:0046326	positive regulation of D-glucose import	0.43035	2
GO:0051385	response to mineralocorticoid	0.43035	2
GO:0097484	dendrite extension	0.43035	2

GO:0099171	presynaptic modulation of chemical synaptic transmission	0.43035	2
GO:0071900	regulation of protein serine/threonine kinase activity	0.43035	6
GO:0048592	eye morphogenesis	0.432526	5
GO:0007088	regulation of mitotic nuclear division	0.434517	4
GO:1903522	regulation of blood circulation	0.435502	7
GO:0042176	regulation of protein catabolic process	0.436077	9
GO:0009880	embryonic pattern specification	0.436077	3
GO:0048488	synaptic vesicle endocytosis	0.436077	3
GO:0002699	positive regulation of immune effector process	0.43924	7
GO:1901888	regulation of cell junction assembly	0.43924	7
GO:0002263	cell activation involved in immune response	0.440228	8
GO:0006904	vesicle docking involved in exocytosis	0.440228	2
GO:0010664	negative regulation of striated muscle cell apoptotic process	0.440228	2
GO:0010934	macrophage cytokine production	0.440228	2
GO:0010935	regulation of macrophage cytokine production	0.440228	2
GO:0035909	aorta morphogenesis	0.440228	2
GO:0043243	positive regulation of protein-containing complex disassembly	0.440228	2
GO:0046677	response to antibiotic	0.440228	2
GO:0048730	epidermis morphogenesis	0.440228	2
GO:1902229	regulation of intrinsic apoptotic signaling pathway in response to DNA damage	0.440228	2
GO:0032890	regulation of organic acid transport	0.440228	3
GO:0043536	positive regulation of blood vessel endothelial cell migration	0.440228	3
GO:0046425	regulation of receptor signaling pathway via JAK-STAT	0.440228	3
GO:0048332	mesoderm morphogenesis	0.440228	3
GO:0030217	T cell differentiation	0.440228	8
GO:0043588	skin development	0.440228	8
GO:0050673	epithelial cell proliferation	0.443869	11
GO:0009416	response to light stimulus	0.447292	8

GO:0001937	negative regulation of endothelial cell proliferation	0.447292	3
GO:0044458	motile cilium assembly	0.447292	3
GO:0055117	regulation of cardiac muscle contraction	0.447292	3
GO:0030326	embryonic limb morphogenesis	0.447292	4
GO:0031644	regulation of nervous system process	0.447292	4
GO:0035113	embryonic appendage morphogenesis	0.447292	4
GO:0045446	endothelial cell differentiation	0.447292	4
GO:0003205	cardiac chamber development	0.447292	5
GO:2000058	regulation of ubiquitin-dependent protein catabolic process	0.447292	5
GO:0007601	visual perception	0.447292	6
GO:0014741	negative regulation of muscle hypertrophy	0.449701	2
GO:0042044	fluid transport	0.449701	2
GO:0033674	positive regulation of kinase activity	0.452804	6
GO:0001952	regulation of cell-matrix adhesion	0.453438	4
GO:0046660	female sex differentiation	0.453438	4
GO:0016049	cell growth	0.453438	11
GO:0042886	amide transport	0.453438	8
GO:0010469	regulation of signaling receptor activity	0.454185	3
GO:0045685	regulation of glial cell differentiation	0.454185	3
GO:0010717	regulation of epithelial to mesenchymal transition	0.45918	4
GO:1903305	regulation of regulated secretory pathway	0.45918	4
GO:1901654	response to ketone	0.45918	6
GO:0016266	O-glycan processing	0.45918	2
GO:0045616	regulation of keratinocyte differentiation	0.45918	2
GO:0045730	respiratory burst	0.45918	2
GO:0045745	positive regulation of G protein-coupled receptor signaling pathway	0.45918	2
GO:0048873	homeostasis of number of cells within a tissue	0.45918	2
GO:0070286	axonemal dynein complex assembly	0.45918	2
GO:0072337	modified amino acid transport	0.45918	2
GO:0099590	neurotransmitter receptor internalization	0.45918	2
GO:1902742	apoptotic process involved in development	0.45918	2

GO:0032436	positive regulation of proteasomal ubiquitin-dependent protein catabolic process	0.45937	3
GO:0140238	presynaptic endocytosis	0.45937	3
GO:1903169	regulation of calcium ion transmembrane transport	0.45937	5
GO:0051896	regulation of phosphatidylinositol 3-kinase/protein kinase B signal transduction	0.462281	7
GO:0050953	sensory perception of light stimulus	0.462281	6
GO:0031647	regulation of protein stability	0.464944	9
GO:0030166	proteoglycan biosynthetic process	0.468586	3
GO:0030516	regulation of axon extension	0.468586	3
GO:0043537	negative regulation of blood vessel endothelial cell migration	0.468586	3
GO:0050680	negative regulation of epithelial cell proliferation	0.470338	5
GO:1902905	positive regulation of supramolecular fiber organization	0.470338	5
GO:0030866	cortical actin cytoskeleton organization	0.470338	2
GO:0090199	regulation of release of cytochrome c from mitochondria	0.470338	2
GO:0090329	regulation of DNA-templated DNA replication	0.470338	2
GO:1904994	regulation of leukocyte adhesion to vascular endothelial cell	0.470338	2
GO:0045785	positive regulation of cell adhesion	0.470338	11
GO:0051336	regulation of hydrolase activity	0.472098	8
GO:0003206	cardiac chamber morphogenesis	0.473937	4
GO:0042267	natural killer cell mediated cytotoxicity	0.473937	3
GO:0061333	renal tubule morphogenesis	0.473937	3
GO:0043244	regulation of protein-containing complex disassembly	0.473937	4
GO:0098659	inorganic cation import across plasma membrane	0.473937	4
GO:0099587	inorganic ion import across plasma membrane	0.473937	4
GO:0010951	negative regulation of endopeptidase activity	0.473937	2
GO:0042417	dopamine metabolic process	0.473937	2
GO:0050433	regulation of catecholamine secretion	0.473937	2

GO:0060999	positive regulation of dendritic spine development	0.473937	2
GO:2000403	positive regulation of lymphocyte migration	0.473937	2
GO:0051952	regulation of amine transport	0.473937	3
GO:0048588	developmental cell growth	0.473937	6
GO:0035265	organ growth	0.473937	5
GO:1903034	regulation of response to wounding	0.473937	5
GO:0048872	homeostasis of number of cells	0.473937	8
GO:0002720	positive regulation of cytokine production involved in immune response	0.473937	3
GO:0032418	lysosome localization	0.473937	3
GO:0035050	embryonic heart tube development	0.473937	3
GO:0070509	calcium ion import	0.473937	3
GO:0003014	renal system process	0.473937	4
GO:0014902	myotube differentiation	0.473937	4
GO:0032434	regulation of proteasomal ubiquitin-dependent protein catabolic process	0.473937	4
GO:0002429	immune response-activating cell surface receptor signaling pathway	0.473937	8
GO:0009268	response to pH	0.473937	2
GO:0016202	regulation of striated muscle tissue development	0.473937	2
GO:0046717	acid secretion	0.473937	2
GO:0060412	ventricular septum morphogenesis	0.473937	2
GO:0008544	epidermis development	0.473937	9
GO:0050900	leukocyte migration	0.473937	9
GO:0048732	gland development	0.473937	10
GO:0042113	B cell activation	0.473937	7
GO:0050871	positive regulation of B cell activation	0.473937	3
GO:1990849	vacuolar localization	0.473937	3
GO:0001578	microtubule bundle formation	0.473937	4
GO:0035710	CD4-positive, alpha-beta T cell activation	0.473937	4
GO:0050864	regulation of B cell activation	0.473937	4
GO:0072175	epithelial tube formation	0.473937	4

GO:0007189	adenylate cyclase-activating G protein-coupled receptor signaling pathway	0.473937	5
GO:0017157	regulation of exocytosis	0.473937	5
GO:0042129	regulation of T cell proliferation	0.473937	5
GO:0010828	positive regulation of D-glucose transmembrane transport	0.473937	2
GO:0015872	dopamine transport	0.473937	2
GO:0045494	photoreceptor cell maintenance	0.473937	2
GO:0050892	intestinal absorption	0.473937	2
GO:0098703	calcium ion import across plasma membrane	0.473937	2
GO:1902656	calcium ion import into cytosol	0.473937	2
GO:0008589	regulation of smoothened signaling pathway	0.473937	3
GO:0002685	regulation of leukocyte migration	0.473937	6
GO:0002317	plasma cell differentiation	0.473937	1
GO:0003253	cardiac neural crest cell migration involved in outflow tract morphogenesis	0.473937	1
GO:0006171	cAMP biosynthetic process	0.473937	1
GO:0007198	adenylate cyclase-inhibiting serotonin receptor signaling pathway	0.473937	1
GO:0007440	foregut morphogenesis	0.473937	1
GO:0009235	cobalamin metabolic process	0.473937	1
GO:0014820	tonic smooth muscle contraction	0.473937	1
GO:0019227	neuronal action potential propagation	0.473937	1
GO:0019835	cytolysis	0.473937	1
GO:0021683	cerebellar granular layer morphogenesis	0.473937	1
GO:0030643	intracellular phosphate ion homeostasis	0.473937	1
GO:0034356	NAD biosynthesis via nicotinamide riboside salvage pathway	0.473937	1
GO:0035655	interleukin-18-mediated signaling pathway	0.473937	1
GO:0036109	alpha-linolenic acid metabolic process	0.473937	1
GO:0038129	ERBB3 signaling pathway	0.473937	1
GO:0044351	macropinocytosis	0.473937	1
GO:0045607	regulation of inner ear auditory receptor cell differentiation	0.473937	1
GO:0045631	regulation of mechanoreceptor differentiation	0.473937	1

GO:0046834	lipid phosphorylation	0.473937	1
GO:0048865	stem cell fate commitment	0.473937	1
GO:0050859	negative regulation of B cell receptor signaling pathway	0.473937	1
GO:0051933	amino acid neurotransmitter reuptake	0.473937	1
GO:0060068	vagina development	0.473937	1
GO:0060502	epithelial cell proliferation involved in lung morphogenesis	0.473937	1
GO:0060536	cartilage morphogenesis	0.473937	1
GO:0060768	regulation of epithelial cell proliferation involved in prostate gland development	0.473937	1
GO:0060947	cardiac vascular smooth muscle cell differentiation	0.473937	1
GO:0061626	pharyngeal arch artery morphogenesis	0.473937	1
GO:0070244	negative regulation of thymocyte apoptotic process	0.473937	1
GO:0070344	regulation of fat cell proliferation	0.473937	1
GO:0071287	cellular response to manganese ion	0.473937	1
GO:0071351	cellular response to interleukin-18	0.473937	1
GO:0071374	cellular response to parathyroid hormone stimulus	0.473937	1
GO:0072014	proximal tubule development	0.473937	1
GO:0072205	metanephric collecting duct development	0.473937	1
GO:0090269	fibroblast growth factor production	0.473937	1
GO:0090270	regulation of fibroblast growth factor production	0.473937	1
GO:0097113	AMPA glutamate receptor clustering	0.473937	1
GO:0097503	sialylation	0.473937	1
GO:0097688	glutamate receptor clustering	0.473937	1
GO:0098598	learned vocalization behavior or vocal learning	0.473937	1
GO:1902965	regulation of protein localization to early endosome	0.473937	1
GO:1902966	positive regulation of protein localization to early endosome	0.473937	1
GO:1903596	regulation of gap junction assembly	0.473937	1
GO:1903911	positive regulation of receptor clustering	0.473937	1

GO:1904383	response to sodium phosphate	0.473937	1
GO:2000104	negative regulation of DNA-templated DNA replication	0.473937	1
GO:2000563	positive regulation of CD4-positive, alpha-beta T cell proliferation	0.473937	1
GO:2000766	negative regulation of cytoplasmic translation	0.473937	1
GO:2000980	regulation of inner ear receptor cell differentiation	0.473937	1
GO:0071478	cellular response to radiation	0.473937	5
GO:0002228	natural killer cell mediated immunity	0.473937	3
GO:0003407	neural retina development	0.473937	3
GO:0072665	protein localization to vacuole	0.473937	3
GO:0006284	base-excision repair	0.473937	2
GO:0042554	superoxide anion generation	0.473937	2
GO:0045687	positive regulation of glial cell differentiation	0.473937	2
GO:0048713	regulation of oligodendrocyte differentiation	0.473937	2
GO:0051602	response to electrical stimulus	0.473937	2
GO:0060338	regulation of type I interferon-mediated signaling pathway	0.473937	2
GO:0060428	lung epithelium development	0.473937	2
GO:0090051	negative regulation of cell migration involved in sprouting angiogenesis	0.473937	2
GO:1901861	regulation of muscle tissue development	0.473937	2
GO:0010038	response to metal ion	0.473937	8
GO:0008154	actin polymerization or depolymerization	0.473937	5
GO:0019827	stem cell population maintenance	0.473937	5
GO:0036465	synaptic vesicle recycling	0.473937	3
GO:0042310	vasoconstriction	0.473937	3
GO:0048593	camera-type eye morphogenesis	0.473937	4
GO:0051345	positive regulation of hydrolase activity	0.473937	5
GO:0006739	NADP metabolic process	0.473937	2
GO:0019098	reproductive behavior	0.473937	2
GO:0032892	positive regulation of organic acid transport	0.473937	2
GO:0033574	response to testosterone	0.473937	2
GO:0044546	NLRP3 inflammasome complex assembly	0.473937	2

GO:0045601	regulation of endothelial cell differentiation	0.473937	2
GO:0048512	circadian behavior	0.473937	2
GO:0060711	labyrinthine layer development	0.473937	2
GO:0009953	dorsal/ventral pattern formation	0.473937	3
GO:0032456	endocytic recycling	0.473937	3
GO:0072659	protein localization to plasma membrane	0.473937	7
GO:0016042	lipid catabolic process	0.473937	8
GO:0001959	regulation of cytokine-mediated signaling pathway	0.473937	5
GO:0051338	regulation of transferase activity	0.473937	10
GO:0016358	dendrite development	0.473937	6
GO:0007548	sex differentiation	0.473937	7
GO:0070372	regulation of ERK1 and ERK2 cascade	0.473937	7
GO:0000098	sulfur amino acid catabolic process	0.473937	1
GO:0001821	histamine secretion	0.473937	1
GO:0003139	secondary heart field specification	0.473937	1
GO:0003357	noradrenergic neuron differentiation	0.473937	1
GO:0006182	cGMP biosynthetic process	0.473937	1
GO:0007168	receptor guanylyl cyclase signaling pathway	0.473937	1
GO:0007175	negative regulation of epidermal growth factor-activated receptor activity	0.473937	1
GO:0015791	polyol transmembrane transport	0.473937	1
GO:0019365	pyridine nucleotide salvage	0.473937	1
GO:0019852	L-ascorbic acid metabolic process	0.473937	1
GO:0021860	pyramidal neuron development	0.473937	1
GO:0031125	rRNA 3'-end processing	0.473937	1
GO:0031915	positive regulation of synaptic plasticity	0.473937	1
GO:0032000	positive regulation of fatty acid beta-oxidation	0.473937	1
GO:0032060	bleb assembly	0.473937	1
GO:0032525	somite rostral/caudal axis specification	0.473937	1
GO:0032782	bile acid secretion	0.473937	1
GO:0034115	negative regulation of heterotypic cell-cell adhesion	0.473937	1
GO:0034355	NAD biosynthetic process via the salvage pathway	0.473937	1

GO:0035581	sequestering of extracellular ligand from receptor	0.473937	1
GO:0035666	TRIF-dependent toll-like receptor signaling pathway	0.473937	1
GO:0038130	ERBB4 signaling pathway	0.473937	1
GO:0042118	endothelial cell activation	0.473937	1
GO:0042797	tRNA transcription by RNA polymerase III	0.473937	1
GO:0043652	engulfment of apoptotic cell	0.473937	1
GO:0045719	negative regulation of glycogen biosynthetic process	0.473937	1
GO:0045869	negative regulation of single stranded viral RNA replication via double stranded DNA intermediate	0.473937	1
GO:0046495	nicotinamide riboside metabolic process	0.473937	1
GO:0046689	response to mercury ion	0.473937	1
GO:0046710	GDP metabolic process	0.473937	1
GO:0046794	transport of virus	0.473937	1
GO:0060346	bone trabecula formation	0.473937	1
GO:0060433	bronchus development	0.473937	1
GO:0060439	trachea morphogenesis	0.473937	1
GO:0060601	lateral sprouting from an epithelium	0.473937	1
GO:0060736	prostate gland growth	0.473937	1
GO:0060767	epithelial cell proliferation involved in prostate gland development	0.473937	1
GO:0061470	T follicular helper cell differentiation	0.473937	1
GO:0070587	regulation of cell-cell adhesion involved in gastrulation	0.473937	1
GO:0070637	pyridine nucleoside metabolic process	0.473937	1
GO:0070778	L-aspartate transmembrane transport	0.473937	1
GO:0071492	cellular response to UV-A	0.473937	1
GO:0072488	ammonium transmembrane transport	0.473937	1
GO:0075733	intracellular transport of virus	0.473937	1
GO:0090161	Golgi ribbon formation	0.473937	1
GO:0090331	negative regulation of platelet aggregation	0.473937	1
GO:0098870	action potential propagation	0.473937	1

GO:0099532	synaptic vesicle endosomal processing	0.473937	1
GO:1903276	regulation of sodium ion export across plasma membrane	0.473937	1
GO:1903278	positive regulation of sodium ion export across plasma membrane	0.473937	1
GO:1904238	pericyte cell differentiation	0.473937	1
GO:2000048	negative regulation of cell-cell adhesion mediated by cadherin	0.473937	1
GO:0051897	positive regulation of phosphatidylinositol 3-kinase/protein kinase B signal transduction	0.473937	5
GO:1902600	proton transmembrane transport	0.473937	5
GO:0140029	exocytic process	0.473937	3
GO:0001667	ameboidal-type cell migration	0.473937	6
GO:0003351	epithelial cilium movement involved in extracellular fluid movement	0.473937	2
GO:0006623	protein targeting to vacuole	0.473937	2
GO:0030261	chromosome condensation	0.473937	2
GO:0030279	negative regulation of ossification	0.473937	2
GO:0032480	negative regulation of type I interferon production	0.473937	2
GO:0042771	intrinsic apoptotic signaling pathway in response to DNA damage by p53 class mediator	0.473937	2
GO:0045840	positive regulation of mitotic nuclear division	0.473937	2
GO:0048634	regulation of muscle organ development	0.473937	2
GO:0061138	morphogenesis of a branching epithelium	0.473937	5
GO:0098727	maintenance of cell number	0.473937	5
GO:0007200	phospholipase C-activating G protein-coupled receptor signaling pathway	0.473937	4
GO:0050678	regulation of epithelial cell proliferation	0.473937	9
GO:0010927	cellular component assembly involved in morphogenesis	0.473937	4
GO:0030183	B cell differentiation	0.473937	4
GO:0032989	cellular anatomical entity morphogenesis	0.473937	4
GO:0046651	lymphocyte proliferation	0.473937	7
GO:0007622	rhythmic behavior	0.473937	2

GO:0016339	calcium-dependent cell-cell adhesion via plasma membrane cell adhesion molecules	0.473937	2
GO:0031295	T cell costimulation	0.473937	2
GO:0048806	genitalia development	0.473937	2
GO:0097028	dendritic cell differentiation	0.473937	2
GO:0140632	canonical inflammasome complex assembly	0.473937	2
GO:1902622	regulation of neutrophil migration	0.473937	2
GO:1905145	cellular response to acetylcholine	0.473937	2
GO:0030901	midbrain development	0.473937	3
GO:0050792	regulation of viral process	0.473937	4
GO:1904064	positive regulation of cation transmembrane transport	0.473937	4
GO:0032496	response to lipopolysaccharide	0.473937	8
GO:0006941	striated muscle contraction	0.473937	5
GO:0021543	pallium development	0.473937	5
GO:0043467	regulation of generation of precursor metabolites and energy	0.473937	4
GO:0050770	regulation of axonogenesis	0.473937	4
GO:0014910	regulation of smooth muscle cell migration	0.473937	3
GO:1904705	regulation of vascular associated smooth muscle cell proliferation	0.473937	3
GO:0048511	rhythmic process	0.473937	7
GO:0006775	fat-soluble vitamin metabolic process	0.473937	2
GO:0007094	mitotic spindle assembly checkpoint signaling	0.473937	2
GO:0014003	oligodendrocyte development	0.473937	2
GO:0030850	prostate gland development	0.473937	2
GO:0031294	lymphocyte costimulation	0.473937	2
GO:0050432	catecholamine secretion	0.473937	2
GO:0051489	regulation of filopodium assembly	0.473937	2
GO:0061028	establishment of endothelial barrier	0.473937	2
GO:0071173	spindle assembly checkpoint signaling	0.473937	2
GO:0071174	mitotic spindle checkpoint signaling	0.473937	2
GO:1903170	negative regulation of calcium ion transmembrane transport	0.473937	2
GO:0002328	pro-B cell differentiation	0.473937	1

GO:0003207	cardiac chamber formation	0.473937	1
GO:0009304	tRNA transcription	0.473937	1
GO:0010518	positive regulation of phospholipase activity	0.473937	1
GO:0014854	response to inactivity	0.473937	1
GO:0014889	muscle atrophy	0.473937	1
GO:0015015	heparan sulfate proteoglycan biosynthetic process, enzymatic modification	0.473937	1
GO:0016056	G protein-coupled opsin signaling pathway	0.473937	1
GO:0016081	synaptic vesicle docking	0.473937	1
GO:0016559	peroxisome fission	0.473937	1
GO:0017038	protein import	0.473937	1
GO:0030948	negative regulation of vascular endothelial growth factor receptor signaling pathway	0.473937	1
GO:0031223	auditory behavior	0.473937	1
GO:0031293	membrane protein intracellular domain proteolysis	0.473937	1
GO:0033605	positive regulation of catecholamine secretion	0.473937	1
GO:0034454	microtubule anchoring at centrosome	0.473937	1
GO:0035112	genitalia morphogenesis	0.473937	1
GO:0036295	cellular response to increased oxygen levels	0.473937	1
GO:0038026	reelin-mediated signaling pathway	0.473937	1
GO:0042447	hormone catabolic process	0.473937	1
GO:0042754	negative regulation of circadian rhythm	0.473937	1
GO:0045161	neuronal ion channel clustering	0.473937	1
GO:0045793	positive regulation of cell size	0.473937	1
GO:0048755	branching morphogenesis of a nerve	0.473937	1
GO:0050847	progesterone receptor signaling pathway	0.473937	1
GO:0060148	positive regulation of post-transcriptional gene silencing	0.473937	1
GO:0060442	branching involved in prostate gland morphogenesis	0.473937	1
GO:0061309	cardiac neural crest cell development involved in outflow tract morphogenesis	0.473937	1
GO:0062042	regulation of cardiac epithelial to mesenchymal transition	0.473937	1

GO:0070099	regulation of chemokine-mediated signaling pathway	0.473937	1
GO:0070586	cell-cell adhesion involved in gastrulation	0.473937	1
GO:0070673	response to interleukin-18	0.473937	1
GO:0071236	cellular response to antibiotic	0.473937	1
GO:0071257	cellular response to electrical stimulus	0.473937	1
GO:0071389	cellular response to mineralocorticoid stimulus	0.473937	1
GO:0071394	cellular response to testosterone stimulus	0.473937	1
GO:0071493	cellular response to UV-B	0.473937	1
GO:0071872	cellular response to epinephrine stimulus	0.473937	1
GO:0072017	distal tubule development	0.473937	1
GO:0086100	endothelin receptor signaling pathway	0.473937	1
GO:0090677	reversible differentiation	0.473937	1
GO:0097048	dendritic cell apoptotic process	0.473937	1
GO:0097084	vascular associated smooth muscle cell development	0.473937	1
GO:0097400	interleukin-17-mediated signaling pathway	0.473937	1
GO:0098976	excitatory chemical synaptic transmission	0.473937	1
GO:1900370	positive regulation of post-transcriptional gene silencing by RNA	0.473937	1
GO:1901678	iron coordination entity transport	0.473937	1
GO:1902946	protein localization to early endosome	0.473937	1
GO:1904729	regulation of intestinal lipid absorption	0.473937	1
GO:1905165	regulation of lysosomal protein catabolic process	0.473937	1
GO:1905205	positive regulation of connective tissue replacement	0.473937	1
GO:1905456	regulation of lymphoid progenitor cell differentiation	0.473937	1
GO:1990504	dense core granule exocytosis	0.473937	1
GO:2000637	positive regulation of miRNA-mediated gene silencing	0.473937	1
GO:2000644	regulation of receptor catabolic process	0.473937	1
GO:2000651	positive regulation of sodium ion transmembrane transporter activity	0.473937	1

GO:2000668	regulation of dendritic cell apoptotic process	0.473937	1
GO:0048813	dendrite morphogenesis	0.473937	4
GO:0015807	L-amino acid transport	0.473937	3
GO:0071677	positive regulation of mononuclear cell migration	0.473937	3
GO:0030072	peptide hormone secretion	0.473937	6
GO:0002700	regulation of production of molecular mediator of immune response	0.473937	5
GO:1905475	regulation of protein localization to membrane	0.473937	5
GO:0006858	extracellular transport	0.473937	2
GO:0010907	positive regulation of glucose metabolic process	0.473937	2
GO:0031577	spindle checkpoint signaling	0.473937	2
GO:0035272	exocrine system development	0.473937	2
GO:0035850	epithelial cell differentiation involved in kidney development	0.473937	2
GO:0042491	inner ear auditory receptor cell differentiation	0.473937	2
GO:0044788	modulation by host of viral process	0.473937	2
GO:0045747	positive regulation of Notch signaling pathway	0.473937	2
GO:0060976	coronary vasculature development	0.473937	2
GO:1902116	negative regulation of organelle assembly	0.473937	2
GO:2000404	regulation of T cell migration	0.473937	2
GO:0009913	epidermal cell differentiation	0.473937	6
GO:0043086	negative regulation of catalytic activity	0.473937	7
GO:0002690	positive regulation of leukocyte chemotaxis	0.473937	3
GO:0048704	embryonic skeletal system morphogenesis	0.473937	3
GO:0106027	neuron projection organization	0.473937	3
GO:1990874	vascular associated smooth muscle cell proliferation	0.473937	3
GO:0030317	flagellated sperm motility	0.473937	5
GO:0097722	sperm motility	0.473937	5
GO:0008064	regulation of actin polymerization or depolymerization	0.473937	4
GO:0016055	Wnt signaling pathway	0.473937	10
GO:0030324	lung development	0.473937	5
GO:0032943	mononuclear cell proliferation	0.473937	7

GO:0051924	regulation of calcium ion transport	0.473937	6
GO:1904892	regulation of receptor signaling pathway via STAT	0.473937	3
GO:0050671	positive regulation of lymphocyte proliferation	0.473937	4
GO:0051783	regulation of nuclear division	0.473937	4
GO:0060048	cardiac muscle contraction	0.473937	4
GO:0032365	intracellular lipid transport	0.473937	2
GO:0033046	negative regulation of sister chromatid segregation	0.473937	2
GO:0033048	negative regulation of mitotic sister chromatid segregation	0.473937	2
GO:0045841	negative regulation of mitotic metaphase/anaphase transition	0.473937	2
GO:0051496	positive regulation of stress fiber assembly	0.473937	2
GO:0170036	import into the mitochondrion	0.473937	2
GO:1902305	regulation of sodium ion transmembrane transport	0.473937	2
GO:1905144	response to acetylcholine	0.473937	2
GO:2000816	negative regulation of mitotic sister chromatid separation	0.473937	2
GO:0061136	regulation of proteasomal protein catabolic process	0.473937	5
GO:0043603	amide metabolic process	0.473937	10
GO:0001696	gastric acid secretion	0.473937	1
GO:0002024	diet induced thermogenesis	0.473937	1
GO:0002327	immature B cell differentiation	0.473937	1
GO:0006388	tRNA splicing, via endonucleolytic cleavage and ligation	0.473937	1
GO:0007176	regulation of epidermal growth factor-activated receptor activity	0.473937	1
GO:0007320	insemination	0.473937	1
GO:0007501	mesodermal cell fate specification	0.473937	1
GO:0009415	response to water	0.473937	1
GO:0016185	synaptic vesicle budding from presynaptic endocytic zone membrane	0.473937	1

GO:0019321	pentose metabolic process	0.473937	1
GO:0021794	thalamus development	0.473937	1
GO:0031272	regulation of pseudopodium assembly	0.473937	1
GO:0031274	positive regulation of pseudopodium assembly	0.473937	1
GO:0032966	negative regulation of collagen biosynthetic process	0.473937	1
GO:0033083	regulation of immature T cell proliferation	0.473937	1
GO:0033084	regulation of immature T cell proliferation in thymus	0.473937	1
GO:0034111	negative regulation of homotypic cell-cell adhesion	0.473937	1
GO:0042416	dopamine biosynthetic process	0.473937	1
GO:0042761	very long-chain fatty acid biosynthetic process	0.473937	1
GO:0045039	protein insertion into mitochondrial inner membrane	0.473937	1
GO:0048103	somatic stem cell division	0.473937	1
GO:0050655	dermatan sulfate proteoglycan metabolic process	0.473937	1
GO:0060075	regulation of resting membrane potential	0.473937	1
GO:0060134	prepulse inhibition	0.473937	1
GO:0060287	epithelial cilium movement involved in determination of left/right asymmetry	0.473937	1
GO:0060670	branching involved in labyrinthine layer morphogenesis	0.473937	1
GO:0061517	macrophage proliferation	0.473937	1
GO:0061684	chaperone-mediated autophagy	0.473937	1
GO:0061795	Golgi lumen acidification	0.473937	1
GO:0070170	regulation of tooth mineralization	0.473937	1
GO:0070278	extracellular matrix constituent secretion	0.473937	1
GO:0070294	renal sodium ion absorption	0.473937	1
GO:0070417	cellular response to cold	0.473937	1
GO:0070486	leukocyte aggregation	0.473937	1
GO:0070874	negative regulation of glycogen metabolic process	0.473937	1
GO:0071472	cellular response to salt stress	0.473937	1

GO:1900103	positive regulation of endoplasmic reticulum unfolded protein response	0.473937	1
GO:1900115	extracellular regulation of signal transduction	0.473937	1
GO:1900116	extracellular negative regulation of signal transduction	0.473937	1
GO:1901386	negative regulation of voltage-gated calcium channel activity	0.473937	1
GO:1902337	regulation of apoptotic process involved in morphogenesis	0.473937	1
GO:1902902	negative regulation of autophagosome assembly	0.473937	1
GO:1903977	positive regulation of glial cell migration	0.473937	1
GO:1904995	negative regulation of leukocyte adhesion to vascular endothelial cell	0.473937	1
GO:1905064	negative regulation of vascular associated smooth muscle cell differentiation	0.473937	1
GO:1905065	positive regulation of vascular associated smooth muscle cell differentiation	0.473937	1
GO:1905668	positive regulation of protein localization to endosome	0.473937	1
GO:1905939	regulation of gonad development	0.473937	1
GO:0007224	smoothened signaling pathway	0.473937	4
GO:0010631	epithelial cell migration	0.473937	3
GO:0051899	membrane depolarization	0.473937	3
GO:0071346	cellular response to type II interferon	0.473937	3
GO:0046631	alpha-beta T cell activation	0.473937	5
GO:1904062	regulation of monoatomic cation transmembrane transport	0.473937	7
GO:0010466	negative regulation of peptidase activity	0.473937	2
GO:0042461	photoreceptor cell development	0.473937	2
GO:0050922	negative regulation of chemotaxis	0.473937	2
GO:0051155	positive regulation of striated muscle cell differentiation	0.473937	2
GO:0089718	amino acid import across plasma membrane	0.473937	2
GO:0030832	regulation of actin filament length	0.473937	4

GO:2001235	positive regulation of apoptotic signaling pathway	0.473937	4
GO:0002833	positive regulation of response to biotic stimulus	0.473937	9
GO:0007162	negative regulation of cell adhesion	0.473937	7
GO:0060996	dendritic spine development	0.473937	3
GO:0061387	regulation of extent of cell growth	0.473937	3
GO:0002790	peptide secretion	0.473937	6
GO:0032946	positive regulation of mononuclear cell proliferation	0.473937	4
GO:0030323	respiratory tube development	0.473937	5
GO:0031099	regeneration	0.473937	5
GO:0001657	ureteric bud development	0.473937	3
GO:0051346	negative regulation of hydrolase activity	0.473937	3
GO:0060349	bone morphogenesis	0.473937	3
GO:0010043	response to zinc ion	0.473937	2
GO:0014075	response to amine	0.473937	2
GO:0019083	viral transcription	0.473937	2
GO:0032964	collagen biosynthetic process	0.473937	2
GO:0043303	mast cell degranulation	0.473937	2
GO:0044331	cell-cell adhesion mediated by cadherin	0.473937	2
GO:0090102	cochlea development	0.473937	2
GO:0140353	lipid export from cell	0.473937	2
GO:0141084	inflammasome-mediated signaling pathway	0.473937	2
GO:1902100	negative regulation of metaphase/anaphase transition of cell cycle	0.473937	2
GO:1905819	negative regulation of chromosome separation	0.473937	2
GO:2000179	positive regulation of neural precursor cell proliferation	0.473937	2
GO:0050852	T cell receptor signaling pathway	0.473937	4
GO:0010001	glial cell differentiation	0.473937	6
GO:0006942	regulation of striated muscle contraction	0.473937	3
GO:0010596	negative regulation of endothelial cell migration	0.473937	3
GO:0072163	mesonephric epithelium development	0.473937	3
GO:0072164	mesonephric tubule development	0.473937	3

GO:0090132	epithelium migration	0.473937	3
GO:0001890	placenta development	0.473937	4
GO:0034767	positive regulation of monoatomic ion transmembrane transport	0.473937	4
GO:0000394	RNA splicing, via endonucleolytic cleavage and ligation	0.473937	1
GO:0002486	antigen processing and presentation of endogenous peptide antigen via MHC class I via ER pathway, TAP-independent	0.473937	1
GO:0003413	chondrocyte differentiation involved in endochondral bone morphogenesis	0.473937	1
GO:0007638	mechanosensory behavior	0.473937	1
GO:0009214	cyclic nucleotide catabolic process	0.473937	1
GO:0010524	positive regulation of calcium ion transport into cytosol	0.473937	1
GO:0010713	negative regulation of collagen metabolic process	0.473937	1
GO:0021681	cerebellar granular layer development	0.473937	1
GO:0030011	maintenance of cell polarity	0.473937	1
GO:0030497	fatty acid elongation	0.473937	1
GO:0032536	regulation of cell projection size	0.473937	1
GO:0033079	immature T cell proliferation	0.473937	1
GO:0033080	immature T cell proliferation in thymus	0.473937	1
GO:0033262	regulation of nuclear cell cycle DNA replication	0.473937	1
GO:0042363	fat-soluble vitamin catabolic process	0.473937	1
GO:0042670	retinal cone cell differentiation	0.473937	1
GO:0043353	enucleate erythrocyte differentiation	0.473937	1
GO:0043922	negative regulation by host of viral transcription	0.473937	1
GO:0045091	regulation of single stranded viral RNA replication via double stranded DNA intermediate	0.473937	1
GO:0045605	negative regulation of epidermal cell differentiation	0.473937	1
GO:0045683	negative regulation of epidermis development	0.473937	1
GO:0046655	folic acid metabolic process	0.473937	1

GO:0048388	endosomal lumen acidification	0.473937	1
GO:0048715	negative regulation of oligodendrocyte differentiation	0.473937	1
GO:0051255	spindle midzone assembly	0.473937	1
GO:0051608	histamine transport	0.473937	1
GO:0051764	actin crosslink formation	0.473937	1
GO:0060354	negative regulation of cell adhesion molecule production	0.473937	1
GO:0060837	blood vessel endothelial cell differentiation	0.473937	1
GO:0061000	negative regulation of dendritic spine development	0.473937	1
GO:0061430	bone trabecula morphogenesis	0.473937	1
GO:0070141	response to UV-A	0.473937	1
GO:0070307	lens fiber cell development	0.473937	1
GO:0070986	left/right axis specification	0.473937	1
GO:0071107	response to parathyroid hormone	0.473937	1
GO:0071285	cellular response to lithium ion	0.473937	1
GO:0072393	microtubule anchoring at microtubule organizing center	0.473937	1
GO:0140052	cellular response to oxidised low-density lipoprotein particle stimulus	0.473937	1
GO:1900426	positive regulation of defense response to bacterium	0.473937	1
GO:1901334	lactone metabolic process	0.473937	1
GO:1904350	regulation of protein catabolic process in the vacuole	0.473937	1
GO:1904380	endoplasmic reticulum mannose trimming	0.473937	1
GO:1904478	regulation of intestinal absorption	0.473937	1
GO:1904748	regulation of apoptotic process involved in development	0.473937	1
GO:1905666	regulation of protein localization to endosome	0.473937	1
GO:1905907	negative regulation of amyloid fibril formation	0.473937	1
GO:2000105	positive regulation of DNA-templated DNA replication	0.473937	1
GO:2000303	regulation of ceramide biosynthetic process	0.473937	1

GO:2001279	regulation of unsaturated fatty acid biosynthetic process	0.473937	1
GO:0005996	monosaccharide metabolic process	0.473961	6
GO:0001836	release of cytochrome c from mitochondria	0.474582	2
GO:0002548	monocyte chemotaxis	0.474582	2
GO:0010559	regulation of glycoprotein biosynthetic process	0.474582	2
GO:0051985	negative regulation of chromosome segregation	0.474582	2
GO:0071622	regulation of granulocyte chemotaxis	0.474582	2
GO:0120316	sperm flagellum assembly	0.474582	2
GO:1904752	regulation of vascular associated smooth muscle cell migration	0.474582	2
GO:0090101	negative regulation of transmembrane receptor protein serine/threonine kinase signaling pathway	0.474582	5
GO:0046467	membrane lipid biosynthetic process	0.474582	4
GO:0016079	synaptic vesicle exocytosis	0.474582	3
GO:0042147	retrograde transport, endosome to Golgi	0.474582	3
GO:0043279	response to alkaloid	0.474582	3
GO:0048534	hematopoietic or lymphoid organ development	0.474582	3
GO:0016051	carbohydrate biosynthetic process	0.474582	5
GO:0001649	osteoblast differentiation	0.474582	6
GO:0046883	regulation of hormone secretion	0.474582	6
GO:0051347	positive regulation of transferase activity	0.474582	6
GO:0007215	glutamate receptor signaling pathway	0.474582	2
GO:0010665	regulation of cardiac muscle cell apoptotic process	0.474582	2
GO:0034381	plasma lipoprotein particle clearance	0.474582	2
GO:0050885	neuromuscular process controlling balance	0.474582	2
GO:0060425	lung morphogenesis	0.474582	2
GO:0001763	morphogenesis of a branching structure	0.474582	5
GO:0015914	phospholipid transport	0.474582	3
GO:0030101	natural killer cell activation	0.474582	3
GO:0035082	axoneme assembly	0.474582	3
GO:0046879	hormone secretion	0.474582	7

GO:0002705	positive regulation of leukocyte mediated immunity	0.474582	4
GO:0022408	negative regulation of cell-cell adhesion	0.474582	5
GO:0051047	positive regulation of secretion	0.474582	7
GO:0002237	response to molecule of bacterial origin	0.474582	8
GO:0010508	positive regulation of autophagy	0.474582	4
GO:0001833	inner cell mass cell proliferation	0.474582	1
GO:0002031	G protein-coupled receptor internalization	0.474582	1
GO:0002476	antigen processing and presentation of endogenous peptide antigen via MHC class Ib	0.474582	1
GO:0002484	antigen processing and presentation of endogenous peptide antigen via MHC class I via ER pathway	0.474582	1
GO:0003198	epithelial to mesenchymal transition involved in endocardial cushion formation	0.474582	1
GO:0007567	parturition	0.474582	1
GO:0010763	positive regulation of fibroblast migration	0.474582	1
GO:0010989	negative regulation of low-density lipoprotein particle clearance	0.474582	1
GO:0014004	microglia differentiation	0.474582	1
GO:0014029	neural crest formation	0.474582	1
GO:0015670	carbon dioxide transport	0.474582	1
GO:0018230	peptidyl-L-cysteine S-palmitoylation	0.474582	1
GO:0018231	peptidyl-S-diacylglycerol-L-cysteine biosynthetic process from peptidyl-cysteine	0.474582	1
GO:0021859	pyramidal neuron differentiation	0.474582	1
GO:0030007	intracellular potassium ion homeostasis	0.474582	1
GO:0032621	interleukin-18 production	0.474582	1
GO:0032661	regulation of interleukin-18 production	0.474582	1
GO:0034501	protein localization to kinetochore	0.474582	1
GO:0035860	glial cell-derived neurotrophic factor receptor signaling pathway	0.474582	1
GO:0038083	peptidyl-tyrosine autophosphorylation	0.474582	1
GO:0039692	single stranded viral RNA replication via double stranded DNA intermediate	0.474582	1

GO:0043201	response to L-leucine	0.474582	1
GO:0045019	negative regulation of nitric oxide biosynthetic process	0.474582	1
GO:0048791	calcium ion-regulated exocytosis of neurotransmitter	0.474582	1
GO:0050930	induction of positive chemotaxis	0.474582	1
GO:0050966	detection of mechanical stimulus involved in sensory perception of pain	0.474582	1
GO:0051412	response to corticosterone	0.474582	1
GO:0055012	ventricular cardiac muscle cell differentiation	0.474582	1
GO:0055057	neuroblast division	0.474582	1
GO:0060330	regulation of response to type II interferon	0.474582	1
GO:0060334	regulation of type II interferon-mediated signaling pathway	0.474582	1
GO:0060457	negative regulation of digestive system process	0.474582	1
GO:0060572	morphogenesis of an epithelial bud	0.474582	1
GO:0061307	cardiac neural crest cell differentiation involved in heart development	0.474582	1
GO:0061308	cardiac neural crest cell development involved in heart development	0.474582	1
GO:0070142	synaptic vesicle budding	0.474582	1
GO:0070243	regulation of thymocyte apoptotic process	0.474582	1
GO:0070341	fat cell proliferation	0.474582	1
GO:0071361	cellular response to ethanol	0.474582	1
GO:0071871	response to epinephrine	0.474582	1
GO:0072148	epithelial cell fate commitment	0.474582	1
GO:0090153	regulation of sphingolipid biosynthetic process	0.474582	1
GO:0098693	regulation of synaptic vesicle cycle	0.474582	1
GO:0150011	regulation of neuron projection arborization	0.474582	1
GO:1900452	regulation of long-term synaptic depression	0.474582	1
GO:1901533	negative regulation of hematopoietic progenitor cell differentiation	0.474582	1
GO:1903083	protein localization to condensed chromosome	0.474582	1
GO:1903894	regulation of IRE1-mediated unfolded protein response	0.474582	1

GO:1904251	regulation of bile acid metabolic process	0.474582	1
GO:1904406	negative regulation of nitric oxide metabolic process	0.474582	1
GO:1905203	regulation of connective tissue replacement	0.474582	1
GO:2001028	positive regulation of endothelial cell chemotaxis	0.474582	1
GO:0043270	positive regulation of monoatomic ion transport	0.474582	5
GO:0002279	mast cell activation involved in immune response	0.474582	2
GO:0002448	mast cell mediated immunity	0.474582	2
GO:0002639	positive regulation of immunoglobulin production	0.474582	2
GO:0010761	fibroblast migration	0.474582	2
GO:2000059	negative regulation of ubiquitin-dependent protein catabolic process	0.474582	2
GO:0062197	cellular response to chemical stress	0.478834	7
GO:0001823	mesonephros development	0.478834	3
GO:0009062	fatty acid catabolic process	0.478834	3
GO:0043255	regulation of carbohydrate biosynthetic process	0.478834	3
GO:0009411	response to UV	0.478834	4
GO:0032368	regulation of lipid transport	0.478834	4
GO:0000381	regulation of alternative mRNA splicing, via spliceosome	0.483264	2
GO:0001895	retina homeostasis	0.483264	2
GO:0002762	negative regulation of myeloid leukocyte differentiation	0.483264	2
GO:0002886	regulation of myeloid leukocyte mediated immunity	0.483264	2
GO:0032147	activation of protein kinase activity	0.483264	2
GO:0071385	cellular response to glucocorticoid stimulus	0.483264	2
GO:0006885	regulation of pH	0.483264	3
GO:0008585	female gonad development	0.483264	3
GO:0032526	response to retinoic acid	0.483264	3
GO:0090130	tissue migration	0.483264	3

GO:1905477	positive regulation of protein localization to membrane	0.483264	3
GO:0050851	antigen receptor-mediated signaling pathway	0.483264	5
GO:0009314	response to radiation	0.483264	9
GO:0045807	positive regulation of endocytosis	0.483264	4
GO:0019395	fatty acid oxidation	0.483264	3
GO:0043367	CD4-positive, alpha-beta T cell differentiation	0.483264	3
GO:0006584	catecholamine metabolic process	0.483264	2
GO:0009712	catechol-containing compound metabolic process	0.483264	2
GO:0010659	cardiac muscle cell apoptotic process	0.483264	2
GO:0010662	regulation of striated muscle cell apoptotic process	0.483264	2
GO:0030042	actin filament depolymerization	0.483264	2
GO:0033047	regulation of mitotic sister chromatid segregation	0.483264	2
GO:0035315	hair cell differentiation	0.483264	2
GO:0061756	leukocyte adhesion to vascular endothelial cell	0.483264	2
GO:0002428	antigen processing and presentation of peptide antigen via MHC class Ib	0.483264	1
GO:0002756	MyD88-independent toll-like receptor signaling pathway	0.483264	1
GO:0003096	renal sodium ion transport	0.483264	1
GO:0003214	cardiac left ventricle morphogenesis	0.483264	1
GO:0006361	transcription initiation at RNA polymerase I promoter	0.483264	1
GO:0009111	vitamin catabolic process	0.483264	1
GO:0010226	response to lithium ion	0.483264	1
GO:0016045	detection of bacterium	0.483264	1
GO:0031269	pseudopodium assembly	0.483264	1
GO:0032897	negative regulation of viral transcription	0.483264	1
GO:0034349	glial cell apoptotic process	0.483264	1
GO:0034616	response to laminar fluid shear stress	0.483264	1
GO:0042415	norepinephrine metabolic process	0.483264	1
GO:0042795	snRNA transcription by RNA polymerase II	0.483264	1

GO:0046321	positive regulation of fatty acid oxidation	0.483264	1
GO:0046325	negative regulation of D-glucose import	0.483264	1
GO:0048308	organelle inheritance	0.483264	1
GO:0048313	Golgi inheritance	0.483264	1
GO:0051969	regulation of transmission of nerve impulse	0.483264	1
GO:0060841	venous blood vessel development	0.483264	1
GO:0061098	positive regulation of protein tyrosine kinase activity	0.483264	1
GO:0061099	negative regulation of protein tyrosine kinase activity	0.483264	1
GO:0072498	embryonic skeletal joint development	0.483264	1
GO:0072531	pyrimidine-containing compound transmembrane transport	0.483264	1
GO:1903727	positive regulation of phospholipid metabolic process	0.483264	1
GO:1903909	regulation of receptor clustering	0.483264	1
GO:1904177	regulation of adipose tissue development	0.483264	1
GO:0016525	negative regulation of angiogenesis	0.483835	4
GO:0007626	locomotory behavior	0.485733	5
GO:0031032	actomyosin structure organization	0.485733	5
GO:0050829	defense response to Gram-negative bacterium	0.487836	3
GO:0007043	cell-cell junction assembly	0.489626	4
GO:0045839	negative regulation of mitotic nuclear division	0.491098	2
GO:0060135	maternal process involved in female pregnancy	0.491098	2
GO:0061005	cell differentiation involved in kidney development	0.491098	2
GO:1901019	regulation of calcium ion transmembrane transporter activity	0.491098	2
GO:2000107	negative regulation of leukocyte apoptotic process	0.491098	2
GO:0003333	amino acid transmembrane transport	0.491537	3
GO:0019217	regulation of fatty acid metabolic process	0.491537	3
GO:0060079	excitatory postsynaptic potential	0.491537	3
GO:0060761	negative regulation of response to cytokine stimulus	0.491537	3

GO:0051494	negative regulation of cytoskeleton organization	0.491537	4
GO:2000181	negative regulation of blood vessel morphogenesis	0.491537	4
GO:0042098	T cell proliferation	0.491537	5
GO:0060294	cilium movement involved in cell motility	0.491537	5
GO:0009914	hormone transport	0.491537	7
GO:0055074	calcium ion homeostasis	0.491537	7
GO:0032102	negative regulation of response to external stimulus	0.491537	9
GO:0050679	positive regulation of epithelial cell proliferation	0.491537	5
GO:0050729	positive regulation of inflammatory response	0.491537	4
GO:0048168	regulation of neuronal synaptic plasticity	0.491537	2
GO:1903018	regulation of glycoprotein metabolic process	0.491537	2
GO:0001708	cell fate specification	0.491537	3
GO:0006029	proteoglycan metabolic process	0.491537	3
GO:0046545	development of primary female sexual characteristics	0.491537	3
GO:0006570	tyrosine metabolic process	0.491537	1
GO:0009070	serine family amino acid biosynthetic process	0.491537	1
GO:0010042	response to manganese ion	0.491537	1
GO:0010766	negative regulation of sodium ion transport	0.491537	1
GO:0010958	regulation of amino acid import across plasma membrane	0.491537	1
GO:0021535	cell migration in hindbrain	0.491537	1
GO:0030852	regulation of granulocyte differentiation	0.491537	1
GO:0031054	pre-miRNA processing	0.491537	1
GO:0031268	pseudopodium organization	0.491537	1
GO:0031645	negative regulation of nervous system process	0.491537	1
GO:0036159	inner dynein arm assembly	0.491537	1
GO:0036166	phenotypic switching	0.491537	1
GO:0044320	cellular response to leptin stimulus	0.491537	1
GO:0044790	suppression of viral release by host	0.491537	1
GO:0048557	embryonic digestive tract morphogenesis	0.491537	1
GO:0048841	regulation of axon extension involved in axon guidance	0.491537	1

GO:0050872	white fat cell differentiation	0.491537	1
GO:0050910	detection of mechanical stimulus involved in sensory perception of sound	0.491537	1
GO:0055089	fatty acid homeostasis	0.491537	1
GO:0060253	negative regulation of glial cell proliferation	0.491537	1
GO:0071732	cellular response to nitric oxide	0.491537	1
GO:0072109	glomerular mesangium development	0.491537	1
GO:0072224	metanephric glomerulus development	0.491537	1
GO:0090136	epithelial cell-cell adhesion	0.491537	1
GO:0090399	replicative senescence	0.491537	1
GO:0110156	mRNA methylguanosine-cap decapping	0.491537	1
GO:1901096	regulation of autophagosome maturation	0.491537	1
GO:1901550	regulation of endothelial cell development	0.491537	1
GO:1903140	regulation of establishment of endothelial barrier	0.491537	1
GO:1903789	regulation of amino acid transmembrane transport	0.491537	1
GO:0060070	canonical Wnt signaling pathway	0.491689	7
GO:0060348	bone development	0.491689	5
GO:0071356	cellular response to tumor necrosis factor	0.491689	5
GO:1901343	negative regulation of vasculature development	0.492913	4
GO:0032611	interleukin-1 beta production	0.494913	3
GO:0032651	regulation of interleukin-1 beta production	0.494913	3
GO:0060291	long-term synaptic potentiation	0.494913	3
GO:0001539	cilium or flagellum-dependent cell motility	0.495745	5
GO:0060285	cilium-dependent cell motility	0.495745	5
GO:0001658	branching involved in ureteric bud morphogenesis	0.496464	2
GO:0010658	striated muscle cell apoptotic process	0.496464	2
GO:0014009	glial cell proliferation	0.496464	2
GO:0032233	positive regulation of actin filament bundle assembly	0.496464	2
GO:0006865	amino acid transport	0.496735	4
GO:0014013	regulation of gliogenesis	0.498576	3
GO:0032963	collagen metabolic process	0.498576	3

GO:0035051	cardiocyte differentiation	0.498576	4
GO:0045089	positive regulation of innate immune response	0.498576	8
GO:0050863	regulation of T cell activation	0.498576	8
GO:0060271	cilium assembly	0.498576	8
GO:0010965	regulation of mitotic sister chromatid separation	0.498576	2
GO:0045599	negative regulation of fat cell differentiation	0.498576	2
GO:0051785	positive regulation of nuclear division	0.498576	2
GO:0006664	glycolipid metabolic process	0.498576	3
GO:0006672	ceramide metabolic process	0.498576	3
GO:0030316	osteoclast differentiation	0.498576	3
GO:0043473	pigmentation	0.498576	3
GO:1902476	chloride transmembrane transport	0.498576	3
GO:0006606	protein import into nucleus	0.498576	4
GO:0022411	cellular component disassembly	0.498576	9
GO:0048545	response to steroid hormone	0.498576	7
GO:0001710	mesodermal cell fate commitment	0.498576	1
GO:0003128	heart field specification	0.498576	1
GO:0006089	lactate metabolic process	0.498576	1
GO:0009404	toxin metabolic process	0.498576	1
GO:0010310	regulation of hydrogen peroxide metabolic process	0.498576	1
GO:0010517	regulation of phospholipase activity	0.498576	1
GO:0010544	negative regulation of platelet activation	0.498576	1
GO:0010755	regulation of plasminogen activation	0.498576	1
GO:0016264	gap junction assembly	0.498576	1
GO:0030299	intestinal cholesterol absorption	0.498576	1
GO:0030889	negative regulation of B cell proliferation	0.498576	1
GO:0033631	cell-cell adhesion mediated by integrin	0.498576	1
GO:0035234	ectopic germ cell programmed cell death	0.498576	1
GO:0035728	response to hepatocyte growth factor	0.498576	1
GO:0036507	protein demannosylation	0.498576	1
GO:0036508	protein alpha-1,2-demannosylation	0.498576	1
GO:0045475	locomotor rhythm	0.498576	1
GO:0046579	positive regulation of Ras protein signal transduction	0.498576	1

GO:0061101	neuroendocrine cell differentiation	0.498576	1
GO:0072578	neurotransmitter-gated ion channel clustering	0.498576	1
GO:0097396	response to interleukin-17	0.498576	1
GO:0097398	cellular response to interleukin-17	0.498576	1
GO:0097401	synaptic vesicle lumen acidification	0.498576	1
GO:0098664	G protein-coupled serotonin receptor signaling pathway	0.498576	1
GO:0099638	endosome to plasma membrane protein transport	0.498576	1
GO:1900006	positive regulation of dendrite development	0.498576	1
GO:1901018	positive regulation of potassium ion transmembrane transporter activity	0.498576	1
GO:1903236	regulation of leukocyte tethering or rolling	0.498576	1
GO:0030198	extracellular matrix organization	0.502124	7
GO:0032412	regulation of monoatomic ion transmembrane transporter activity	0.502916	4
GO:0034440	lipid oxidation	0.503219	3
GO:1903509	liposaccharide metabolic process	0.503219	3
GO:0008347	glial cell migration	0.504203	2
GO:0051341	regulation of oxidoreductase activity	0.504203	2
GO:0043062	extracellular structure organization	0.504966	7
GO:0007605	sensory perception of sound	0.506248	4
GO:0009100	glycoprotein metabolic process	0.506248	8
GO:0045229	external encapsulating structure organization	0.506248	7
GO:1903900	regulation of viral life cycle	0.506248	3
GO:0015833	peptide transport	0.506248	6
GO:0060541	respiratory system development	0.506248	5
GO:0033013	tetrapyrrole metabolic process	0.506248	2
GO:0034142	toll-like receptor 4 signaling pathway	0.506248	2
GO:0048524	positive regulation of viral process	0.506248	2
GO:0048599	oocyte development	0.506248	2
GO:0098900	regulation of action potential	0.506248	2
GO:0042742	defense response to bacterium	0.506248	7
GO:0002062	chondrocyte differentiation	0.506248	3
GO:0002444	myeloid leukocyte mediated immunity	0.506248	3

GO:0002029	desensitization of G protein-coupled receptor signaling pathway	0.506248	1
GO:0002281	macrophage activation involved in immune response	0.506248	1
GO:0006684	sphingomyelin metabolic process	0.506248	1
GO:0007603	phototransduction, visible light	0.506248	1
GO:0007620	copulation	0.506248	1
GO:0016137	glycoside metabolic process	0.506248	1
GO:0030206	chondroitin sulfate biosynthetic process	0.506248	1
GO:0030540	female genitalia development	0.506248	1
GO:0030575	nuclear body organization	0.506248	1
GO:0031053	primary miRNA processing	0.506248	1
GO:0032026	response to magnesium ion	0.506248	1
GO:0035338	long-chain fatty-acyl-CoA biosynthetic process	0.506248	1
GO:0036035	osteoclast development	0.506248	1
GO:0036342	post-anal tail morphogenesis	0.506248	1
GO:0042481	regulation of odontogenesis	0.506248	1
GO:0045603	positive regulation of endothelial cell differentiation	0.506248	1
GO:0048490	anterograde synaptic vesicle transport	0.506248	1
GO:0050862	positive regulation of T cell receptor signaling pathway	0.506248	1
GO:0050884	neuromuscular process controlling posture	0.506248	1
GO:0051132	NK T cell activation	0.506248	1
GO:0060039	pericardium development	0.506248	1
GO:0060088	auditory receptor cell stereocilium organization	0.506248	1
GO:0060438	trachea development	0.506248	1
GO:0060965	negative regulation of miRNA-mediated gene silencing	0.506248	1
GO:0090083	regulation of inclusion body assembly	0.506248	1
GO:0098543	detection of other organism	0.506248	1
GO:0098751	bone cell development	0.506248	1
GO:0099514	synaptic vesicle cytoskeletal transport	0.506248	1
GO:0099517	synaptic vesicle transport along microtubule	0.506248	1
GO:1902170	cellular response to reactive nitrogen species	0.506248	1

GO:1902514	regulation of calcium ion transmembrane transport via high voltage-gated calcium channel	0.506248	1
GO:1903232	melanosome assembly	0.506248	1
GO:1905906	regulation of amyloid fibril formation	0.506248	1
GO:0043524	negative regulation of neuron apoptotic process	0.507186	4
GO:0010959	regulation of metal ion transport	0.507698	8
GO:0007163	establishment or maintenance of cell polarity	0.509367	5
GO:0001756	somitogenesis	0.509367	2
GO:0001947	heart looping	0.509367	2
GO:0021545	cranial nerve development	0.509367	2
GO:0051306	mitotic sister chromatid separation	0.509367	2
GO:0051784	negative regulation of nuclear division	0.509367	2
GO:0051937	catecholamine transport	0.509367	2
GO:0072666	establishment of protein localization to vacuole	0.509367	2
GO:0019882	antigen processing and presentation	0.509367	3
GO:0051928	positive regulation of calcium ion transport	0.509367	3
GO:0034765	regulation of monoatomic ion transmembrane transport	0.509367	7
GO:0001678	intracellular glucose homeostasis	0.509367	4
GO:0015748	organophosphate ester transport	0.509367	4
GO:0051170	import into nucleus	0.509367	4
GO:0007009	plasma membrane organization	0.509979	4
GO:0015698	inorganic anion transport	0.509979	4
GO:1903076	regulation of protein localization to plasma membrane	0.509979	3
GO:0001655	urogenital system development	0.509979	2
GO:0001885	endothelial cell development	0.509979	2
GO:0006383	transcription by RNA polymerase III	0.509979	2
GO:0017156	calcium-ion regulated exocytosis	0.509979	2
GO:0050688	regulation of defense response to virus	0.509979	2
GO:0070059	intrinsic apoptotic signaling pathway in response to endoplasmic reticulum stress	0.509979	2
GO:0071384	cellular response to corticosteroid stimulus	0.509979	2

GO:0043161	proteasome-mediated ubiquitin-dependent protein catabolic process	0.509979	9
GO:0002440	production of molecular mediator of immune response	0.509979	5
GO:0002577	regulation of antigen processing and presentation	0.509979	1
GO:0003184	pulmonary valve morphogenesis	0.509979	1
GO:0003215	cardiac right ventricle morphogenesis	0.509979	1
GO:0006098	pentose-phosphate shunt	0.509979	1
GO:0007096	regulation of exit from mitosis	0.509979	1
GO:0009301	snRNA transcription	0.509979	1
GO:0010522	regulation of calcium ion transport into cytosol	0.509979	1
GO:0015810	aspartate transmembrane transport	0.509979	1
GO:0022401	negative adaptation of signaling pathway	0.509979	1
GO:0023058	adaptation of signaling pathway	0.509979	1
GO:0031954	positive regulation of protein autophosphorylation	0.509979	1
GO:0031998	regulation of fatty acid beta-oxidation	0.509979	1
GO:0032634	interleukin-5 production	0.509979	1
GO:0032674	regulation of interleukin-5 production	0.509979	1
GO:0034260	negative regulation of GTPase activity	0.509979	1
GO:0043171	peptide catabolic process	0.509979	1
GO:0043651	linoleic acid metabolic process	0.509979	1
GO:0043691	reverse cholesterol transport	0.509979	1
GO:0045618	positive regulation of keratinocyte differentiation	0.509979	1
GO:0046782	regulation of viral transcription	0.509979	1
GO:0048266	behavioral response to pain	0.509979	1
GO:0051580	regulation of neurotransmitter uptake	0.509979	1
GO:0055062	phosphate ion homeostasis	0.509979	1
GO:0060149	negative regulation of post-transcriptional gene silencing	0.509979	1
GO:0060353	regulation of cell adhesion molecule production	0.509979	1
GO:0060602	branch elongation of an epithelium	0.509979	1

GO:0060967	negative regulation of gene silencing by regulatory ncRNA	0.509979	1
GO:0071474	cellular hyperosmotic response	0.509979	1
GO:0071625	vocalization behavior	0.509979	1
GO:0090201	negative regulation of release of cytochrome c from mitochondria	0.509979	1
GO:0110154	RNA decapping	0.509979	1
GO:1900037	regulation of cellular response to hypoxia	0.509979	1
GO:1900221	regulation of amyloid-beta clearance	0.509979	1
GO:1900369	negative regulation of post-transcriptional gene silencing by regulatory ncRNA	0.509979	1
GO:1900424	regulation of defense response to bacterium	0.509979	1
GO:1902236	negative regulation of endoplasmic reticulum stress-induced intrinsic apoptotic signaling pathway	0.509979	1
GO:1903427	negative regulation of reactive oxygen species biosynthetic process	0.509979	1
GO:0060041	retina development in camera-type eye	0.514359	4
GO:0006893	Golgi to plasma membrane transport	0.514428	2
GO:0051926	negative regulation of calcium ion transport	0.514428	2
GO:0060042	retina morphogenesis in camera-type eye	0.514428	2
GO:0061951	establishment of protein localization to plasma membrane	0.514428	2
GO:0022898	regulation of transmembrane transporter activity	0.517868	4
GO:0050905	neuromuscular process	0.517868	4
GO:0043269	regulation of monoatomic ion transport	0.517868	9
GO:0030595	leukocyte chemotaxis	0.517868	5
GO:0006275	regulation of DNA replication	0.517868	3
GO:0006661	phosphatidylinositol biosynthetic process	0.517868	3
GO:0034341	response to type II interferon	0.517868	3
GO:0001912	positive regulation of leukocyte mediated cytotoxicity	0.517868	2
GO:0009994	oocyte differentiation	0.517868	2
GO:0045668	negative regulation of osteoblast differentiation	0.517868	2

GO:0051057	positive regulation of small GTPase mediated signal transduction	0.517868	2
GO:0060675	ureteric bud morphogenesis	0.517868	2
GO:0030216	keratinocyte differentiation	0.517868	4
GO:0050806	positive regulation of synaptic transmission	0.517868	4
GO:0002888	positive regulation of myeloid leukocyte mediated immunity	0.517868	1
GO:0006517	protein deglycosylation	0.517868	1
GO:0007398	ectoderm development	0.517868	1
GO:0007413	axonal fasciculation	0.517868	1
GO:0010224	response to UV-B	0.517868	1
GO:0010829	negative regulation of D-glucose transmembrane transport	0.517868	1
GO:0010985	negative regulation of lipoprotein particle clearance	0.517868	1
GO:0030502	negative regulation of bone mineralization	0.517868	1
GO:0032261	purine nucleotide salvage	0.517868	1
GO:0032495	response to muramyl dipeptide	0.517868	1
GO:0032616	interleukin-13 production	0.517868	1
GO:0032656	regulation of interleukin-13 production	0.517868	1
GO:0038166	angiotensin-activated signaling pathway	0.517868	1
GO:0043586	tongue development	0.517868	1
GO:0048535	lymph node development	0.517868	1
GO:0048846	axon extension involved in axon guidance	0.517868	1
GO:0050965	detection of temperature stimulus involved in sensory perception of pain	0.517868	1
GO:0051788	response to misfolded protein	0.517868	1
GO:0061050	regulation of cell growth involved in cardiac muscle cell development	0.517868	1
GO:0061577	calcium ion transmembrane transport via high voltage-gated calcium channel	0.517868	1
GO:0070242	thymocyte apoptotic process	0.517868	1
GO:0071514	genomic imprinting	0.517868	1
GO:0090200	positive regulation of release of cytochrome c from mitochondria	0.517868	1

GO:0106030	neuron projection fasciculation	0.517868	1
GO:1900102	negative regulation of endoplasmic reticulum unfolded protein response	0.517868	1
GO:1902284	neuron projection extension involved in neuron projection guidance	0.517868	1
GO:2000010	positive regulation of protein localization to cell surface	0.517868	1
GO:2000269	regulation of fibroblast apoptotic process	0.517868	1
GO:2001044	regulation of integrin-mediated signaling pathway	0.517868	1
GO:0019080	viral gene expression	0.519319	3
GO:0051261	protein depolymerization	0.519319	3
GO:0002697	regulation of immune effector process	0.520288	8
GO:1902115	regulation of organelle assembly	0.520476	5
GO:0010632	regulation of epithelial cell migration	0.520476	2
GO:0019229	regulation of vasoconstriction	0.520476	2
GO:0030199	collagen fibril organization	0.520476	2
GO:0045576	mast cell activation	0.520476	2
GO:0046847	filopodium assembly	0.520476	2
GO:0061180	mammary gland epithelium development	0.520476	2
GO:0072171	mesonephric tubule morphogenesis	0.520476	2
GO:0019058	viral life cycle	0.520553	6
GO:0001936	regulation of endothelial cell proliferation	0.525328	4
GO:0010803	regulation of tumor necrosis factor-mediated signaling pathway	0.525328	2
GO:0060113	inner ear receptor cell differentiation	0.525328	2
GO:0061371	determination of heart left/right asymmetry	0.525328	2
GO:2000401	regulation of lymphocyte migration	0.525328	2
GO:0006690	icosanoid metabolic process	0.525328	3
GO:0019079	viral genome replication	0.525328	3
GO:0071887	leukocyte apoptotic process	0.525328	3
GO:0072329	monocarboxylic acid catabolic process	0.525328	3
GO:0001780	neutrophil homeostasis	0.525328	1
GO:0002093	auditory receptor cell morphogenesis	0.525328	1
GO:0006907	pinocytosis	0.525328	1

GO:0010560	positive regulation of glycoprotein biosynthetic process	0.525328	1
GO:0010737	protein kinase A signaling	0.525328	1
GO:0010988	regulation of low-density lipoprotein particle clearance	0.525328	1
GO:0015669	gas transport	0.525328	1
GO:0019885	antigen processing and presentation of endogenous peptide antigen via MHC class I	0.525328	1
GO:0021554	optic nerve development	0.525328	1
GO:0021756	striatum development	0.525328	1
GO:0030204	chondroitin sulfate metabolic process	0.525328	1
GO:0032816	positive regulation of natural killer cell activation	0.525328	1
GO:0035739	CD4-positive, alpha-beta T cell proliferation	0.525328	1
GO:0043249	erythrocyte maturation	0.525328	1
GO:0045056	transcytosis	0.525328	1
GO:0048485	sympathetic nervous system development	0.525328	1
GO:0060004	reflex	0.525328	1
GO:0060193	positive regulation of lipase activity	0.525328	1
GO:0060445	branching involved in salivary gland morphogenesis	0.525328	1
GO:0060546	negative regulation of necroptotic process	0.525328	1
GO:0071731	response to nitric oxide	0.525328	1
GO:0097709	connective tissue replacement	0.525328	1
GO:1903054	negative regulation of extracellular matrix organization	0.525328	1
GO:1905038	regulation of membrane lipid metabolic process	0.525328	1
GO:1905146	lysosomal protein catabolic process	0.525328	1
GO:2000561	regulation of CD4-positive, alpha-beta T cell proliferation	0.525328	1
GO:0030178	negative regulation of Wnt signaling pathway	0.525328	4
GO:0003143	embryonic heart tube morphogenesis	0.529502	2
GO:0030225	macrophage differentiation	0.529502	2
GO:0031343	positive regulation of cell killing	0.529502	2
GO:0046513	ceramide biosynthetic process	0.529502	2

GO:0048002	antigen processing and presentation of peptide antigen	0.529502	2
GO:0016032	viral process	0.531589	8
GO:0034612	response to tumor necrosis factor	0.531589	5
GO:0002718	regulation of cytokine production involved in immune response	0.531589	3
GO:0010811	positive regulation of cell-substrate adhesion	0.531589	3
GO:0002262	myeloid cell homeostasis	0.531589	4
GO:0006839	mitochondrial transport	0.531589	4
GO:0006879	intracellular iron ion homeostasis	0.531589	2
GO:0043124	negative regulation of canonical NF-kappaB signal transduction	0.531589	2
GO:0046626	regulation of insulin receptor signaling pathway	0.531589	2
GO:0051101	regulation of DNA binding	0.531589	2
GO:0140895	cell surface toll-like receptor signaling pathway	0.531589	2
GO:1990778	protein localization to cell periphery	0.531589	7
GO:0002367	cytokine production involved in immune response	0.531589	3
GO:0002688	regulation of leukocyte chemotaxis	0.531589	3
GO:0022612	gland morphogenesis	0.531589	3
GO:0001832	blastocyst growth	0.531589	1
GO:0002475	antigen processing and presentation via MHC class Ib	0.531589	1
GO:0006740	NADPH regeneration	0.531589	1
GO:0009713	catechol-containing compound biosynthetic process	0.531589	1
GO:0010881	regulation of cardiac muscle contraction by regulation of the release of sequestered calcium ion	0.531589	1
GO:0010955	negative regulation of protein processing	0.531589	1
GO:0032253	dense core granule localization	0.531589	1
GO:0034105	positive regulation of tissue remodeling	0.531589	1
GO:0035493	SNARE complex assembly	0.531589	1
GO:0035855	megakaryocyte development	0.531589	1

GO:0036092	phosphatidylinositol-3-phosphate biosynthetic process	0.531589	1
GO:0036158	outer dynein arm assembly	0.531589	1
GO:0038128	ERBB2 signaling pathway	0.531589	1
GO:0042423	catecholamine biosynthetic process	0.531589	1
GO:0042745	circadian sleep/wake cycle	0.531589	1
GO:0046479	glycosphingolipid catabolic process	0.531589	1
GO:0051151	negative regulation of smooth muscle cell differentiation	0.531589	1
GO:0051988	regulation of attachment of spindle microtubules to kinetochore	0.531589	1
GO:0060352	cell adhesion molecule production	0.531589	1
GO:0061760	antifungal innate immune response	0.531589	1
GO:0070166	enamel mineralization	0.531589	1
GO:0071305	cellular response to vitamin D	0.531589	1
GO:0090026	positive regulation of monocyte chemotaxis	0.531589	1
GO:0097062	dendritic spine maintenance	0.531589	1
GO:1900273	positive regulation of long-term synaptic potentiation	0.531589	1
GO:2000047	regulation of cell-cell adhesion mediated by cadherin	0.531589	1
GO:2000114	regulation of establishment of cell polarity	0.531589	1
GO:2000272	negative regulation of signaling receptor activity	0.531589	1
GO:0032722	positive regulation of chemokine production	0.536366	2
GO:0045665	negative regulation of neuron differentiation	0.536366	2
GO:0045670	regulation of osteoclast differentiation	0.536366	2
GO:0050854	regulation of antigen receptor-mediated signaling pathway	0.536366	2
GO:0070830	bicellular tight junction assembly	0.536366	2
GO:0044782	cilium organization	0.536366	8
GO:0006906	vesicle fusion	0.536366	3
GO:0051495	positive regulation of cytoskeleton organization	0.536988	4
GO:0055088	lipid homeostasis	0.536988	4
GO:0006821	chloride transport	0.536988	3
GO:0032479	regulation of type I interferon production	0.536988	3

GO:0032606	type I interferon production	0.536988	3
GO:0007405	neuroblast proliferation	0.536988	2
GO:0030514	negative regulation of BMP signaling pathway	0.536988	2
GO:0051865	protein autoubiquitination	0.536988	2
GO:1903053	regulation of extracellular matrix organization	0.536988	2
GO:0007219	Notch signaling pathway	0.536988	4
GO:0006874	intracellular calcium ion homeostasis	0.536988	6
GO:0002483	antigen processing and presentation of endogenous peptide antigen	0.536988	1
GO:0003177	pulmonary valve development	0.536988	1
GO:0007042	lysosomal lumen acidification	0.536988	1
GO:0010839	negative regulation of keratinocyte proliferation	0.536988	1
GO:0021871	forebrain regionalization	0.536988	1
GO:0030318	melanocyte differentiation	0.536988	1
GO:0030431	sleep	0.536988	1
GO:0030539	male genitalia development	0.536988	1
GO:0032098	regulation of appetite	0.536988	1
GO:0032770	positive regulation of monooxygenase activity	0.536988	1
GO:0034114	regulation of heterotypic cell-cell adhesion	0.536988	1
GO:0035162	embryonic hemopoiesis	0.536988	1
GO:0042531	positive regulation of tyrosine phosphorylation of STAT protein	0.536988	1
GO:0043153	entrainment of circadian clock by photoperiod	0.536988	1
GO:0045061	thymic T cell selection	0.536988	1
GO:0045063	T-helper 1 cell differentiation	0.536988	1
GO:0045947	negative regulation of translational initiation	0.536988	1
GO:0046641	positive regulation of alpha-beta T cell proliferation	0.536988	1
GO:0046835	carbohydrate phosphorylation	0.536988	1
GO:0048015	phosphatidylinositol-mediated signaling	0.536988	1
GO:0050650	chondroitin sulfate proteoglycan biosynthetic process	0.536988	1
GO:0060339	negative regulation of type I interferon-mediated signaling pathway	0.536988	1
GO:0060571	morphogenesis of an epithelial fold	0.536988	1

GO:0061318	renal filtration cell differentiation	0.536988	1
GO:0062099	negative regulation of programmed necrotic cell death	0.536988	1
GO:0071294	cellular response to zinc ion	0.536988	1
GO:0072112	podocyte differentiation	0.536988	1
GO:0086064	cell communication by electrical coupling involved in cardiac conduction	0.536988	1
GO:0098856	intestinal lipid absorption	0.536988	1
GO:0141156	cAMP/PKA signal transduction	0.536988	1
GO:1902018	negative regulation of cilium assembly	0.536988	1
GO:1903861	positive regulation of dendrite extension	0.536988	1
GO:0002761	regulation of myeloid leukocyte differentiation	0.53783	3
GO:0021987	cerebral cortex development	0.53783	3
GO:0043271	negative regulation of monoatomic ion transport	0.53783	3
GO:0055007	cardiac muscle cell differentiation	0.53783	3
GO:0090174	organelle membrane fusion	0.53783	3
GO:0042490	mechanoreceptor differentiation	0.540691	2
GO:1901880	negative regulation of protein depolymerization	0.540691	2
GO:1904888	cranial skeletal system development	0.540691	2
GO:0007259	cell surface receptor signaling pathway via JAK-STAT	0.540691	3
GO:0010977	negative regulation of neuron projection development	0.540691	3
GO:0032612	interleukin-1 production	0.540691	3
GO:0032652	regulation of interleukin-1 production	0.540691	3
GO:0045471	response to ethanol	0.540691	3
GO:1905952	regulation of lipid localization	0.540691	4
GO:0043254	regulation of protein-containing complex assembly	0.540691	8
GO:0030100	regulation of endocytosis	0.540691	6
GO:0030098	lymphocyte differentiation	0.540691	8
GO:0000380	alternative mRNA splicing, via spliceosome	0.540691	2
GO:0032008	positive regulation of TOR signaling	0.540691	2
GO:0045661	regulation of myoblast differentiation	0.540691	2
GO:0048525	negative regulation of viral process	0.540691	2

GO:0061077	chaperone-mediated protein folding	0.540691	2
GO:0072678	T cell migration	0.540691	2
GO:0001704	formation of primary germ layer	0.540691	3
GO:0098661	inorganic anion transmembrane transport	0.540691	3
GO:0003091	renal water homeostasis	0.540691	1
GO:0006706	steroid catabolic process	0.540691	1
GO:0006817	phosphate ion transport	0.540691	1
GO:0010561	negative regulation of glycoprotein biosynthetic process	0.540691	1
GO:0010623	programmed cell death involved in cell development	0.540691	1
GO:0010842	retina layer formation	0.540691	1
GO:0016082	synaptic vesicle priming	0.540691	1
GO:0030813	positive regulation of nucleotide catabolic process	0.540691	1
GO:0030859	polarized epithelial cell differentiation	0.540691	1
GO:0033123	positive regulation of purine nucleotide catabolic process	0.540691	1
GO:0035336	long-chain fatty-acyl-CoA metabolic process	0.540691	1
GO:0042537	benzene-containing compound metabolic process	0.540691	1
GO:0043101	purine-containing compound salvage	0.540691	1
GO:0045821	positive regulation of glycolytic process	0.540691	1
GO:0050855	regulation of B cell receptor signaling pathway	0.540691	1
GO:0050961	detection of temperature stimulus involved in sensory perception	0.540691	1
GO:0060155	platelet dense granule organization	0.540691	1
GO:0070233	negative regulation of T cell apoptotic process	0.540691	1
GO:0070861	regulation of protein exit from endoplasmic reticulum	0.540691	1
GO:0071280	cellular response to copper ion	0.540691	1
GO:0072311	glomerular epithelial cell differentiation	0.540691	1
GO:0090280	positive regulation of calcium ion import	0.540691	1
GO:0097150	neuronal stem cell population maintenance	0.540691	1
GO:0150105	protein localization to cell-cell junction	0.540691	1

GO:1901020	negative regulation of calcium ion transmembrane transporter activity	0.540691	1
GO:1903020	positive regulation of glycoprotein metabolic process	0.540691	1
GO:1903318	negative regulation of protein maturation	0.540691	1
GO:1903902	positive regulation of viral life cycle	0.540691	1
GO:1905563	negative regulation of vascular endothelial cell proliferation	0.540691	1
GO:0007159	leukocyte cell-cell adhesion	0.540691	8
GO:0032640	tumor necrosis factor production	0.542778	4
GO:0032680	regulation of tumor necrosis factor production	0.542778	4
GO:0097553	calcium ion transmembrane import into cytosol	0.542778	4
GO:0006801	superoxide metabolic process	0.546019	2
GO:0015844	monoamine transport	0.546019	2
GO:0045669	positive regulation of osteoblast differentiation	0.546019	2
GO:1902017	regulation of cilium assembly	0.546019	2
GO:1905818	regulation of chromosome separation	0.546019	2
GO:0030512	negative regulation of transforming growth factor beta receptor signaling pathway	0.546747	3
GO:0006006	glucose metabolic process	0.546747	4
GO:0002637	regulation of immunoglobulin production	0.546747	2
GO:0071277	cellular response to calcium ion	0.546747	2
GO:0072078	nephron tubule morphogenesis	0.546747	2
GO:0002026	regulation of the force of heart contraction	0.546747	1
GO:0007039	protein catabolic process in the vacuole	0.546747	1
GO:0009435	NAD biosynthetic process	0.546747	1
GO:0014829	vascular associated smooth muscle contraction	0.546747	1
GO:0017121	plasma membrane phospholipid scrambling	0.546747	1
GO:0018345	protein palmitoylation	0.546747	1
GO:0019377	glycolipid catabolic process	0.546747	1
GO:0019755	one-carbon compound transport	0.546747	1
GO:0021542	dentate gyrus development	0.546747	1
GO:0022038	corpus callosum development	0.546747	1
GO:0032252	secretory granule localization	0.546747	1

GO:0032878	regulation of establishment or maintenance of cell polarity	0.546747	1
GO:0033622	integrin activation	0.546747	1
GO:0034138	toll-like receptor 3 signaling pathway	0.546747	1
GO:0035988	chondrocyte proliferation	0.546747	1
GO:0043501	skeletal muscle adaptation	0.546747	1
GO:0046339	diacylglycerol metabolic process	0.546747	1
GO:0048596	embryonic camera-type eye morphogenesis	0.546747	1
GO:0050927	positive regulation of positive chemotaxis	0.546747	1
GO:0051446	positive regulation of meiotic cell cycle	0.546747	1
GO:0060561	apoptotic process involved in morphogenesis	0.546747	1
GO:0090103	cochlea morphogenesis	0.546747	1
GO:0098581	detection of external biotic stimulus	0.546747	1
GO:1900227	positive regulation of NLRP3 inflammasome complex assembly	0.546747	1
GO:1901021	positive regulation of calcium ion transmembrane transporter activity	0.546747	1
GO:1901385	regulation of voltage-gated calcium channel activity	0.546747	1
GO:1903588	negative regulation of blood vessel endothelial cell proliferation involved in sprouting angiogenesis	0.546747	1
GO:1904996	positive regulation of leukocyte adhesion to vascular endothelial cell	0.546747	1
GO:2000773	negative regulation of cellular senescence	0.546747	1
GO:2000810	regulation of bicellular tight junction assembly	0.546747	1
GO:0002244	hematopoietic progenitor cell differentiation	0.547744	3
GO:0021782	glial cell development	0.547744	3
GO:0008217	regulation of blood pressure	0.548371	4
GO:0030258	lipid modification	0.548371	4
GO:0045664	regulation of neuron differentiation	0.551879	4
GO:0006469	negative regulation of protein kinase activity	0.551879	3
GO:0031397	negative regulation of protein ubiquitination	0.551879	2
GO:0120192	tight junction assembly	0.551879	2

GO:0071706	tumor necrosis factor superfamily cytokine production	0.551879	4
GO:1903555	regulation of tumor necrosis factor superfamily cytokine production	0.551879	4
GO:0006639	acylglycerol metabolic process	0.551879	3
GO:0030879	mammary gland development	0.551879	3
GO:0001783	B cell apoptotic process	0.551879	1
GO:0006760	folic acid-containing compound metabolic process	0.551879	1
GO:0006972	hyperosmotic response	0.551879	1
GO:0009648	photoperiodism	0.551879	1
GO:0010460	positive regulation of heart rate	0.551879	1
GO:0016188	synaptic vesicle maturation	0.551879	1
GO:0016540	protein autoprocessing	0.551879	1
GO:0021846	cell proliferation in forebrain	0.551879	1
GO:0022010	central nervous system myelination	0.551879	1
GO:0031639	plasminogen activation	0.551879	1
GO:0032291	axon ensheathment in central nervous system	0.551879	1
GO:0032469	endoplasmic reticulum calcium ion homeostasis	0.551879	1
GO:0032925	regulation of activin receptor signaling pathway	0.551879	1
GO:0035235	ionotropic glutamate receptor signaling pathway	0.551879	1
GO:0035902	response to immobilization stress	0.551879	1
GO:0036010	protein localization to endosome	0.551879	1
GO:0036296	response to increased oxygen levels	0.551879	1
GO:0042104	positive regulation of activated T cell proliferation	0.551879	1
GO:0042983	amyloid precursor protein biosynthetic process	0.551879	1
GO:0042984	regulation of amyloid precursor protein biosynthetic process	0.551879	1
GO:0044346	fibroblast apoptotic process	0.551879	1
GO:0044827	modulation by host of viral genome replication	0.551879	1
GO:0045662	negative regulation of myoblast differentiation	0.551879	1
GO:0046037	GMP metabolic process	0.551879	1
GO:0060333	type II interferon-mediated signaling pathway	0.551879	1
GO:0060343	trabecula formation	0.551879	1

GO:0060396	growth hormone receptor signaling pathway	0.551879	1
GO:0071108	protein K48-linked deubiquitination	0.551879	1
GO:0098719	sodium ion import across plasma membrane	0.551879	1
GO:0141087	positive regulation of inflammasome-mediated signaling pathway	0.551879	1
GO:1901679	nucleotide transmembrane transport	0.551879	1
GO:2000737	negative regulation of stem cell differentiation	0.551879	1
GO:0006360	transcription by RNA polymerase I	0.551879	2
GO:0035914	skeletal muscle cell differentiation	0.551879	2
GO:0045069	regulation of viral genome replication	0.551879	2
GO:0072088	nephron epithelium morphogenesis	0.551879	2
GO:0006638	neutral lipid metabolic process	0.554835	3
GO:0030218	erythrocyte differentiation	0.554835	3
GO:0045739	positive regulation of DNA repair	0.554835	3
GO:0046632	alpha-beta T cell differentiation	0.554835	3
GO:0051053	negative regulation of DNA metabolic process	0.554835	3
GO:0006164	purine nucleotide biosynthetic process	0.555526	4
GO:0002752	cell surface pattern recognition receptor signaling pathway	0.555526	2
GO:0014032	neural crest cell development	0.555526	2
GO:0021536	diencephalon development	0.555526	2
GO:0043299	leukocyte degranulation	0.555526	2
GO:0090049	regulation of cell migration involved in sprouting angiogenesis	0.555526	2
GO:0006195	purine nucleotide catabolic process	0.555526	3
GO:0035270	endocrine system development	0.555526	3
GO:0046890	regulation of lipid biosynthetic process	0.555526	4
GO:0002703	regulation of leukocyte mediated immunity	0.555526	5
GO:0003341	cilium movement	0.555526	5
GO:0001964	startle response	0.555526	1
GO:0003209	cardiac atrium morphogenesis	0.555526	1
GO:0003272	endocardial cushion formation	0.555526	1
GO:0003416	endochondral bone growth	0.555526	1
GO:0007097	nuclear migration	0.555526	1
GO:0007289	spermatid nucleus differentiation	0.555526	1

GO:0010818	T cell chemotaxis	0.555526	1
GO:0010971	positive regulation of G2/M transition of mitotic cell cycle	0.555526	1
GO:0015740	C4-dicarboxylate transport	0.555526	1
GO:0016048	detection of temperature stimulus	0.555526	1
GO:0019359	nicotinamide nucleotide biosynthetic process	0.555526	1
GO:0019363	pyridine nucleotide biosynthetic process	0.555526	1
GO:0021544	subpallium development	0.555526	1
GO:0030325	adrenal gland development	0.555526	1
GO:0035025	positive regulation of Rho protein signal transduction	0.555526	1
GO:0035116	embryonic hindlimb morphogenesis	0.555526	1
GO:0042059	negative regulation of epidermal growth factor receptor signaling pathway	0.555526	1
GO:0043173	nucleotide salvage	0.555526	1
GO:0043304	regulation of mast cell degranulation	0.555526	1
GO:0046949	fatty-acyl-CoA biosynthetic process	0.555526	1
GO:0050857	positive regulation of antigen receptor-mediated signaling pathway	0.555526	1
GO:0060117	auditory receptor cell development	0.555526	1
GO:0070536	protein K63-linked deubiquitination	0.555526	1
GO:0071378	cellular response to growth hormone stimulus	0.555526	1
GO:0072010	glomerular epithelium development	0.555526	1
GO:0097186	amelogenesis	0.555526	1
GO:1902221	erythrose 4-phosphate/phosphoenolpyruvate family amino acid metabolic process	0.555526	1
GO:1903019	negative regulation of glycoprotein metabolic process	0.555526	1
GO:1905523	positive regulation of macrophage migration	0.555526	1
GO:0034504	protein localization to nucleus	0.555526	6
GO:0002286	T cell activation involved in immune response	0.555526	3
GO:0007498	mesoderm development	0.555526	3
GO:0030168	platelet activation	0.555526	3
GO:0043433	negative regulation of DNA-binding transcription factor activity	0.555526	3

GO:0090288	negative regulation of cellular response to growth factor stimulus	0.555526	3
GO:0042246	tissue regeneration	0.555526	2
GO:0043297	apical junction assembly	0.555526	2
GO:0072028	nephron morphogenesis	0.555526	2
GO:1904427	positive regulation of calcium ion transmembrane transport	0.555526	2
GO:0032984	protein-containing complex disassembly	0.55623	5
GO:0044248	cellular catabolic process	0.557366	6
GO:0006163	purine nucleotide metabolic process	0.557441	8
GO:0071824	protein-DNA complex organization	0.560048	5
GO:0000302	response to reactive oxygen species	0.560048	4
GO:0031396	regulation of protein ubiquitination	0.560048	4
GO:0016482	cytosolic transport	0.560048	3
GO:0061053	somite development	0.561515	2
GO:0070098	chemokine-mediated signaling pathway	0.561515	2
GO:0001906	cell killing	0.561515	5
GO:0002520	immune system development	0.561515	4
GO:0007369	gastrulation	0.561515	4
GO:0032409	regulation of transporter activity	0.561515	4
GO:0010565	regulation of ketone metabolic process	0.561515	3
GO:0002052	positive regulation of neuroblast proliferation	0.561515	1
GO:0003401	axis elongation	0.561515	1
GO:0006833	water transport	0.561515	1
GO:0009649	entrainment of circadian clock	0.561515	1
GO:0021854	hypothalamus development	0.561515	1
GO:0031952	regulation of protein autophosphorylation	0.561515	1
GO:0042509	regulation of tyrosine phosphorylation of STAT protein	0.561515	1
GO:0045606	positive regulation of epidermal cell differentiation	0.561515	1
GO:0045686	negative regulation of glial cell differentiation	0.561515	1
GO:0045954	positive regulation of natural killer cell mediated cytotoxicity	0.561515	1
GO:0051156	glucose 6-phosphate metabolic process	0.561515	1

GO:0061744	motor behavior	0.561515	1
GO:0070841	inclusion body assembly	0.561515	1
GO:1901201	regulation of extracellular matrix assembly	0.561515	1
GO:1902430	negative regulation of amyloid-beta formation	0.561515	1
GO:2001032	regulation of double-strand break repair via nonhomologous end joining	0.561515	1
GO:0009101	glycoprotein biosynthetic process	0.562492	6
GO:0072522	purine-containing compound biosynthetic process	0.562492	5
GO:0001818	negative regulation of cytokine production	0.562919	7
GO:0001935	endothelial cell proliferation	0.562919	4
GO:0046474	glycerophospholipid biosynthetic process	0.562919	4
GO:0001570	vasculogenesis	0.562919	2
GO:0030593	neutrophil chemotaxis	0.562919	2
GO:0051304	chromosome separation	0.562919	2
GO:0097009	energy homeostasis	0.562919	2
GO:0034763	negative regulation of transmembrane transport	0.564622	3
GO:0090276	regulation of peptide hormone secretion	0.567314	4
GO:0033673	negative regulation of kinase activity	0.569395	3
GO:0043242	negative regulation of protein-containing complex disassembly	0.569395	2
GO:0060337	type I interferon-mediated signaling pathway	0.569395	2
GO:0070227	lymphocyte apoptotic process	0.569395	2
GO:0086003	cardiac muscle cell contraction	0.569395	2
GO:1901657	glycosyl compound metabolic process	0.569395	2
GO:0000002	mitochondrial genome maintenance	0.570213	1
GO:0002507	tolerance induction	0.570213	1
GO:0014047	glutamate secretion	0.570213	1
GO:0032801	receptor catabolic process	0.570213	1
GO:0060402	calcium ion transport into cytosol	0.570213	1
GO:0060441	epithelial tube branching involved in lung morphogenesis	0.570213	1
GO:0062149	detection of stimulus involved in sensory perception of pain	0.570213	1
GO:0071549	cellular response to dexamethasone stimulus	0.570213	1

GO:0090330	regulation of platelet aggregation	0.570213	1
GO:0042593	glucose homeostasis	0.572942	5
GO:0001843	neural tube closure	0.572942	2
GO:0008625	extrinsic apoptotic signaling pathway via death domain receptors	0.572942	2
GO:0042093	T-helper cell differentiation	0.572942	2
GO:0045913	positive regulation of carbohydrate metabolic process	0.572942	2
GO:0071357	cellular response to type I interferon	0.572942	2
GO:0072332	intrinsic apoptotic signaling pathway by p53 class mediator	0.572942	2
GO:0097061	dendritic spine organization	0.572942	2
GO:0002791	regulation of peptide secretion	0.572942	4
GO:0001503	ossification	0.572942	8
GO:0033500	carbohydrate homeostasis	0.572942	5
GO:0046887	positive regulation of hormone secretion	0.572942	3
GO:0046486	glycerolipid metabolic process	0.572942	7
GO:1903037	regulation of leukocyte cell-cell adhesion	0.572942	7
GO:0001892	embryonic placenta development	0.572942	2
GO:0007492	endoderm development	0.572942	2
GO:0048864	stem cell development	0.572942	2
GO:0060606	tube closure	0.572942	2
GO:0120193	tight junction organization	0.572942	2
GO:0009749	response to glucose	0.572942	4
GO:0001963	synaptic transmission, dopaminergic	0.572942	1
GO:0003298	physiological muscle hypertrophy	0.572942	1
GO:0003301	physiological cardiac muscle hypertrophy	0.572942	1
GO:0007026	negative regulation of microtubule depolymerization	0.572942	1
GO:0007274	neuromuscular synaptic transmission	0.572942	1
GO:0019433	triglyceride catabolic process	0.572942	1
GO:0019883	antigen processing and presentation of endogenous antigen	0.572942	1
GO:0021602	cranial nerve morphogenesis	0.572942	1
GO:0030262	apoptotic nuclear changes	0.572942	1

GO:0033688	regulation of osteoblast proliferation	0.572942	1
GO:0045070	positive regulation of viral genome replication	0.572942	1
GO:0050482	arachidonate secretion	0.572942	1
GO:0050654	chondroitin sulfate proteoglycan metabolic process	0.572942	1
GO:0050732	negative regulation of peptidyl-tyrosine phosphorylation	0.572942	1
GO:0050820	positive regulation of coagulation	0.572942	1
GO:0050951	sensory perception of temperature stimulus	0.572942	1
GO:0060964	regulation of miRNA-mediated gene silencing	0.572942	1
GO:0061049	cell growth involved in cardiac muscle cell development	0.572942	1
GO:0071295	cellular response to vitamin	0.572942	1
GO:0072525	pyridine-containing compound biosynthetic process	0.572942	1
GO:0098868	bone growth	0.572942	1
GO:0099505	regulation of presynaptic membrane potential	0.572942	1
GO:0150117	positive regulation of cell-substrate junction organization	0.572942	1
GO:0150146	cell junction disassembly	0.572942	1
GO:1902751	positive regulation of cell cycle G2/M phase transition	0.572942	1
GO:1902992	negative regulation of amyloid precursor protein catabolic process	0.572942	1
GO:1903077	negative regulation of protein localization to plasma membrane	0.572942	1
GO:1903963	arachidonate transport	0.572942	1
GO:0090087	regulation of peptide transport	0.577446	4
GO:0002294	CD4-positive, alpha-beta T cell differentiation involved in immune response	0.578268	2
GO:0007422	peripheral nervous system development	0.578268	2
GO:0071347	cellular response to interleukin-1	0.578268	2
GO:0007269	neurotransmitter secretion	0.578268	3
GO:0071675	regulation of mononuclear cell migration	0.578268	3
GO:0099643	signal release from synapse	0.578268	3

GO:0051260	protein homooligomerization	0.579015	4
GO:0071216	cellular response to biotic stimulus	0.579015	5
GO:0034101	erythrocyte homeostasis	0.579015	3
GO:0034614	cellular response to reactive oxygen species	0.579015	3
GO:0001516	prostaglandin biosynthetic process	0.579015	1
GO:0002313	mature B cell differentiation involved in immune response	0.579015	1
GO:0007271	synaptic transmission, cholinergic	0.579015	1
GO:0009069	serine family amino acid metabolic process	0.579015	1
GO:0010644	cell communication by electrical coupling	0.579015	1
GO:0019082	viral protein processing	0.579015	1
GO:0034311	diol metabolic process	0.579015	1
GO:0035590	purinergic nucleotide receptor signaling pathway	0.579015	1
GO:0035751	regulation of lysosomal lumen pH	0.579015	1
GO:0046457	prostanoid biosynthetic process	0.579015	1
GO:0060603	mammary gland duct morphogenesis	0.579015	1
GO:0090025	regulation of monocyte chemotaxis	0.579015	1
GO:0099022	vesicle tethering	0.579015	1
GO:1900368	regulation of post-transcriptional gene silencing by regulatory ncRNA	0.579015	1
GO:1902042	negative regulation of extrinsic apoptotic signaling pathway via death domain receptors	0.579015	1
GO:2000649	regulation of sodium ion transmembrane transporter activity	0.579015	1
GO:0044703	multi-organism reproductive process	0.579015	4
GO:0048469	cell maturation	0.579015	4
GO:0071383	cellular response to steroid hormone stimulus	0.579015	4
GO:0002287	alpha-beta T cell activation involved in immune response	0.579015	2
GO:0002293	alpha-beta T cell differentiation involved in immune response	0.579015	2
GO:0009791	post-embryonic development	0.579015	2
GO:0033077	T cell differentiation in thymus	0.579015	2
GO:0033627	cell adhesion mediated by integrin	0.579015	2

GO:1902117	positive regulation of organelle assembly	0.579015	2
GO:0051251	positive regulation of lymphocyte activation	0.580254	6
GO:0071333	cellular response to glucose stimulus	0.582794	3
GO:0051453	regulation of intracellular pH	0.586273	2
GO:0006605	protein targeting	0.586273	6
GO:0009746	response to hexose	0.586273	4
GO:0072594	establishment of protein localization to organelle	0.586273	8
GO:0051259	protein complex oligomerization	0.586273	5
GO:0002063	chondrocyte development	0.586273	1
GO:0002230	positive regulation of defense response to virus by host	0.586273	1
GO:0006921	cellular component disassembly involved in execution phase of apoptosis	0.586273	1
GO:0007288	sperm axoneme assembly	0.586273	1
GO:0010458	exit from mitosis	0.586273	1
GO:0010880	regulation of release of sequestered calcium ion into cytosol by sarcoplasmic reticulum	0.586273	1
GO:0010996	response to auditory stimulus	0.586273	1
GO:0015012	heparan sulfate proteoglycan biosynthetic process	0.586273	1
GO:0042558	pteridine-containing compound metabolic process	0.586273	1
GO:0045648	positive regulation of erythrocyte differentiation	0.586273	1
GO:0051497	negative regulation of stress fiber assembly	0.586273	1
GO:0060147	regulation of post-transcriptional gene silencing	0.586273	1
GO:0060351	cartilage development involved in endochondral bone morphogenesis	0.586273	1
GO:0060795	cell fate commitment involved in formation of primary germ layer	0.586273	1
GO:1901185	negative regulation of ERBB signaling pathway	0.586273	1
GO:1903955	positive regulation of protein targeting to mitochondrion	0.586273	1
GO:1904376	negative regulation of protein localization to cell periphery	0.586273	1

GO:1902105	regulation of leukocyte differentiation	0.586273	6
GO:0008306	associative learning	0.587913	2
GO:0045995	regulation of embryonic development	0.587913	2
GO:0048708	astrocyte differentiation	0.587913	2
GO:0030177	positive regulation of Wnt signaling pathway	0.587913	3
GO:0033157	regulation of intracellular protein transport	0.587913	3
GO:0001909	leukocyte mediated cytotoxicity	0.589922	3
GO:0071331	cellular response to hexose stimulus	0.589922	3
GO:0098656	monoatomic anion transmembrane transport	0.589922	3
GO:0002088	lens development in camera-type eye	0.589922	2
GO:0014020	primary neural tube formation	0.589922	2
GO:0034340	response to type I interferon	0.589922	2
GO:1903321	negative regulation of protein modification by small protein conjugation or removal	0.589922	2
GO:1904063	negative regulation of cation transmembrane transport	0.589922	2
GO:1990868	response to chemokine	0.589922	2
GO:1990869	cellular response to chemokine	0.589922	2
GO:2000177	regulation of neural precursor cell proliferation	0.589922	2
GO:2000628	regulation of miRNA metabolic process	0.589922	2
GO:0019216	regulation of lipid metabolic process	0.589922	6
GO:0000096	sulfur amino acid metabolic process	0.589922	1
GO:0007260	tyrosine phosphorylation of STAT protein	0.589922	1
GO:0007435	salivary gland morphogenesis	0.589922	1
GO:0009584	detection of visible light	0.589922	1
GO:0010804	negative regulation of tumor necrosis factor-mediated signaling pathway	0.589922	1
GO:0017145	stem cell division	0.589922	1
GO:0018198	peptidyl-cysteine modification	0.589922	1
GO:0034383	low-density lipoprotein particle clearance	0.589922	1
GO:0045684	positive regulation of epidermis development	0.589922	1
GO:0045879	negative regulation of smoothened signaling pathway	0.589922	1
GO:0050869	negative regulation of B cell activation	0.589922	1

GO:0050891	multicellular organismal-level water homeostasis	0.589922	1
GO:0050901	leukocyte tethering or rolling	0.589922	1
GO:0051647	nucleus localization	0.589922	1
GO:0055075	potassium ion homeostasis	0.589922	1
GO:0060292	long-term synaptic depression	0.589922	1
GO:0060966	regulation of gene silencing by regulatory ncRNA	0.589922	1
GO:0090151	establishment of protein localization to mitochondrial membrane	0.589922	1
GO:1901889	negative regulation of cell junction assembly	0.589922	1
GO:1902235	regulation of endoplasmic reticulum stress-induced intrinsic apoptotic signaling pathway	0.589922	1
GO:2000677	regulation of transcription regulatory region DNA binding	0.589922	1
GO:0046488	phosphatidylinositol metabolic process	0.591141	3
GO:0030071	regulation of mitotic metaphase/anaphase transition	0.593582	2
GO:0097194	execution phase of apoptosis	0.593582	2
GO:1901879	regulation of protein depolymerization	0.593582	2
GO:0071326	cellular response to monosaccharide stimulus	0.594724	3
GO:1904375	regulation of protein localization to cell periphery	0.594724	3
GO:0014033	neural crest cell differentiation	0.594724	2
GO:0021766	hippocampus development	0.594724	2
GO:0030641	regulation of cellular pH	0.594724	2
GO:0042475	odontogenesis of dentin-containing tooth	0.594724	2
GO:0050886	endocrine process	0.594724	2
GO:0055006	cardiac cell development	0.594724	2
GO:0140115	export across plasma membrane	0.594724	2
GO:1900182	positive regulation of protein localization to nucleus	0.594724	2
GO:0009566	fertilization	0.594724	4
GO:0009952	anterior/posterior pattern specification	0.594724	4
GO:0044706	multi-multicellular organism process	0.594724	4

GO:0002495	antigen processing and presentation of peptide antigen via MHC class II	0.594724	1
GO:0002717	positive regulation of natural killer cell mediated immunity	0.594724	1
GO:0006654	phosphatidic acid biosynthetic process	0.594724	1
GO:0007212	G protein-coupled dopamine receptor signaling pathway	0.594724	1
GO:0034063	stress granule assembly	0.594724	1
GO:0034249	negative regulation of amide metabolic process	0.594724	1
GO:0034505	tooth mineralization	0.594724	1
GO:0043045	epigenetic programming of gene expression	0.594724	1
GO:0046320	regulation of fatty acid oxidation	0.594724	1
GO:0048048	embryonic eye morphogenesis	0.594724	1
GO:0050931	pigment cell differentiation	0.594724	1
GO:0060544	regulation of necroptotic process	0.594724	1
GO:0098810	neurotransmitter reuptake	0.594724	1
GO:0110110	positive regulation of animal organ morphogenesis	0.594724	1
GO:1902176	negative regulation of oxidative stress-induced intrinsic apoptotic signaling pathway	0.594724	1
GO:2000463	positive regulation of excitatory postsynaptic potential	0.594724	1
GO:0072523	purine-containing compound catabolic process	0.594724	3
GO:0062012	regulation of small molecule metabolic process	0.595396	6
GO:0034284	response to monosaccharide	0.598573	4
GO:0044403	biological process involved in symbiotic interaction	0.598573	4
GO:0045637	regulation of myeloid cell differentiation	0.598573	4
GO:0009152	purine ribonucleotide biosynthetic process	0.598901	3
GO:0021915	neural tube development	0.598901	3
GO:0006470	protein dephosphorylation	0.598901	2
GO:0010507	negative regulation of autophagy	0.598901	2
GO:0097006	regulation of plasma lipoprotein particle levels	0.598901	2
GO:2000106	regulation of leukocyte apoptotic process	0.598901	2
GO:0002064	epithelial cell development	0.598938	4

GO:1903039	positive regulation of leukocyte cell-cell adhesion	0.598938	5
GO:0009150	purine ribonucleotide metabolic process	0.598938	6
GO:0030111	regulation of Wnt signaling pathway	0.598938	6
GO:0001569	branching involved in blood vessel morphogenesis	0.598938	1
GO:0002021	response to dietary excess	0.598938	1
GO:0002474	antigen processing and presentation of peptide antigen via MHC class I	0.598938	1
GO:0007035	vacuolar acidification	0.598938	1
GO:0010984	regulation of lipoprotein particle clearance	0.598938	1
GO:0021884	forebrain neuron development	0.598938	1
GO:0032232	negative regulation of actin filament bundle assembly	0.598938	1
GO:0032373	positive regulation of sterol transport	0.598938	1
GO:0032376	positive regulation of cholesterol transport	0.598938	1
GO:0032743	positive regulation of interleukin-2 production	0.598938	1
GO:0033687	osteoblast proliferation	0.598938	1
GO:0034143	regulation of toll-like receptor 4 signaling pathway	0.598938	1
GO:0035137	hindlimb morphogenesis	0.598938	1
GO:0035459	vesicle cargo loading	0.598938	1
GO:0045577	regulation of B cell differentiation	0.598938	1
GO:0048665	neuron fate specification	0.598938	1
GO:0090630	activation of GTPase activity	0.598938	1
GO:1901381	positive regulation of potassium ion transmembrane transport	0.598938	1
GO:1905332	positive regulation of morphogenesis of an epithelium	0.598938	1
GO:2000352	negative regulation of endothelial cell apoptotic process	0.598938	1
GO:2000765	regulation of cytoplasmic translation	0.598938	1
GO:0016239	positive regulation of macroautophagy	0.599254	2
GO:0045666	positive regulation of neuron differentiation	0.599254	2

GO:1901874	negative regulation of post-translational protein modification	0.599254	2
GO:1902099	regulation of metaphase/anaphase transition of cell cycle	0.599254	2
GO:0045216	cell-cell junction organization	0.599398	4
GO:0097696	cell surface receptor signaling pathway via STAT	0.601901	3
GO:0015980	energy derivation by oxidation of organic compounds	0.602619	6
GO:0021700	developmental maturation	0.602619	6
GO:0045088	regulation of innate immune response	0.602619	8
GO:0001942	hair follicle development	0.602619	2
GO:0002042	cell migration involved in sprouting angiogenesis	0.602619	2
GO:0002292	T cell differentiation involved in immune response	0.602619	2
GO:0007091	metaphase/anaphase transition of mitotic cell cycle	0.602619	2
GO:0051100	negative regulation of binding	0.602619	2
GO:0061337	cardiac conduction	0.602619	2
GO:2001257	regulation of cation channel activity	0.602619	2
GO:0002221	pattern recognition receptor signaling pathway	0.602619	5
GO:0061351	neural precursor cell proliferation	0.602619	3
GO:0002443	leukocyte mediated immunity	0.602619	8
GO:0002504	antigen processing and presentation of peptide or polysaccharide antigen via MHC class II	0.602619	1
GO:0003161	cardiac conduction system development	0.602619	1
GO:0003230	cardiac atrium development	0.602619	1
GO:0031076	embryonic camera-type eye development	0.602619	1
GO:0031114	regulation of microtubule depolymerization	0.602619	1
GO:0032781	positive regulation of ATP-dependent activity	0.602619	1
GO:0034110	regulation of homotypic cell-cell adhesion	0.602619	1
GO:0034694	response to prostaglandin	0.602619	1
GO:0045880	positive regulation of smoothened signaling pathway	0.602619	1
GO:0046688	response to copper ion	0.602619	1

GO:0048265	response to pain	0.602619	1
GO:0060218	hematopoietic stem cell differentiation	0.602619	1
GO:0071353	cellular response to interleukin-4	0.602619	1
GO:1903580	positive regulation of ATP metabolic process	0.602619	1
GO:1903725	regulation of phospholipid metabolic process	0.602619	1
GO:1903749	positive regulation of establishment of protein localization to mitochondrion	0.602619	1
GO:1904385	cellular response to angiotensin	0.602619	1
GO:0030902	hindbrain development	0.605113	3
GO:0000281	mitotic cytokinesis	0.605113	2
GO:1990542	mitochondrial transmembrane transport	0.605113	2
GO:2001251	negative regulation of chromosome organization	0.605113	2
GO:0009166	nucleotide catabolic process	0.609141	3
GO:0006493	protein O-linked glycosylation	0.609141	2
GO:0009798	axis specification	0.609141	2
GO:0034766	negative regulation of monoatomic ion transmembrane transport	0.609141	2
GO:0051781	positive regulation of cell division	0.609141	2
GO:0051656	establishment of organelle localization	0.609141	8
GO:0001916	positive regulation of T cell mediated cytotoxicity	0.609141	1
GO:0001990	regulation of systemic arterial blood pressure by hormone	0.609141	1
GO:0002335	mature B cell differentiation	0.609141	1
GO:0006901	vesicle coating	0.609141	1
GO:0007431	salivary gland development	0.609141	1
GO:0007616	long-term memory	0.609141	1
GO:0014808	release of sequestered calcium ion into cytosol by sarcoplasmic reticulum	0.609141	1
GO:0021696	cerebellar cortex morphogenesis	0.609141	1
GO:0045589	regulation of regulatory T cell differentiation	0.609141	1
GO:0050858	negative regulation of antigen receptor-mediated signaling pathway	0.609141	1
GO:0070306	lens fiber cell differentiation	0.609141	1
GO:0071398	cellular response to fatty acid	0.609141	1

GO:0071404	cellular response to low-density lipoprotein particle stimulus	0.609141	1
GO:0006282	regulation of DNA repair	0.609351	4
GO:0016485	protein processing	0.609351	4
GO:0001841	neural tube formation	0.611999	2
GO:0001910	regulation of leukocyte mediated cytotoxicity	0.611999	2
GO:0002220	innate immune response activating cell surface receptor signaling pathway	0.611999	2
GO:0006576	biogenic amine metabolic process	0.611999	2
GO:0021510	spinal cord development	0.611999	2
GO:0022404	molting cycle process	0.611999	2
GO:0022405	hair cycle process	0.611999	2
GO:0044784	metaphase/anaphase transition of cell cycle	0.611999	2
GO:0045638	negative regulation of myeloid cell differentiation	0.611999	2
GO:0015850	organic hydroxy compound transport	0.611999	5
GO:0010506	regulation of autophagy	0.61344	6
GO:0006516	glycoprotein catabolic process	0.61344	1
GO:0009595	detection of biotic stimulus	0.61344	1
GO:0017158	regulation of calcium ion-dependent exocytosis	0.61344	1
GO:0032438	melanosome organization	0.61344	1
GO:0033280	response to vitamin D	0.61344	1
GO:0035633	maintenance of blood-brain barrier	0.61344	1
GO:0042401	biogenic amine biosynthetic process	0.61344	1
GO:0042755	eating behavior	0.61344	1
GO:0045823	positive regulation of heart contraction	0.61344	1
GO:0046471	phosphatidylglycerol metabolic process	0.61344	1
GO:0046473	phosphatidic acid metabolic process	0.61344	1
GO:0048741	skeletal muscle fiber development	0.61344	1
GO:0055094	response to lipoprotein particle	0.61344	1
GO:0060122	inner ear receptor cell stereocilium organization	0.61344	1
GO:0060323	head morphogenesis	0.61344	1
GO:0070229	negative regulation of lymphocyte apoptotic process	0.61344	1
GO:0140448	signaling receptor ligand precursor processing	0.61344	1

GO:2000780	negative regulation of double-strand break repair	0.61344	1
GO:0007029	endoplasmic reticulum organization	0.613542	2
GO:0019364	pyridine nucleotide catabolic process	0.613542	2
GO:0034446	substrate adhesion-dependent cell spreading	0.613542	2
GO:0071322	cellular response to carbohydrate stimulus	0.613542	3
GO:0072331	signal transduction by p53 class mediator	0.613542	3
GO:0071674	mononuclear cell migration	0.614694	4
GO:0140694	membraneless organelle assembly	0.616979	7
GO:0002285	lymphocyte activation involved in immune response	0.617227	4
GO:0032755	positive regulation of interleukin-6 production	0.617227	2
GO:0035282	segmentation	0.617227	2
GO:0050773	regulation of dendrite development	0.617227	2
GO:1903532	positive regulation of secretion by cell	0.617227	5
GO:0001941	postsynaptic membrane organization	0.617227	1
GO:0001953	negative regulation of cell-matrix adhesion	0.617227	1
GO:0006688	glycosphingolipid biosynthetic process	0.617227	1
GO:0006734	NADH metabolic process	0.617227	1
GO:0006958	complement activation, classical pathway	0.617227	1
GO:0007340	acrosome reaction	0.617227	1
GO:0009167	purine ribonucleoside monophosphate metabolic process	0.617227	1
GO:0009309	amine biosynthetic process	0.617227	1
GO:0030488	tRNA methylation	0.617227	1
GO:0032435	negative regulation of proteasomal ubiquitin-dependent protein catabolic process	0.617227	1
GO:0048536	spleen development	0.617227	1
GO:0048753	pigment granule organization	0.617227	1
GO:0051954	positive regulation of amine transport	0.617227	1
GO:0060074	synapse maturation	0.617227	1
GO:0060416	response to growth hormone	0.617227	1
GO:0070670	response to interleukin-4	0.617227	1
GO:1903514	release of sequestered calcium ion into cytosol by endoplasmic reticulum	0.617227	1

GO:1903524	positive regulation of blood circulation	0.617227	1
GO:1905476	negative regulation of protein localization to membrane	0.617227	1
GO:1905898	positive regulation of response to endoplasmic reticulum stress	0.617227	1
GO:0071222	cellular response to lipopolysaccharide	0.61726	4
GO:0051348	negative regulation of transferase activity	0.617294	3
GO:0010906	regulation of glucose metabolic process	0.619194	2
GO:0051310	metaphase chromosome alignment	0.619194	2
GO:0051588	regulation of neurotransmitter transport	0.619194	2
GO:0009165	nucleotide biosynthetic process	0.621705	4
GO:0001960	negative regulation of cytokine-mediated signaling pathway	0.621705	2
GO:0072526	pyridine-containing compound catabolic process	0.621705	2
GO:0044272	sulfur compound biosynthetic process	0.621705	3
GO:0003203	endocardial cushion morphogenesis	0.621705	1
GO:0009072	aromatic amino acid metabolic process	0.621705	1
GO:0010762	regulation of fibroblast migration	0.621705	1
GO:0030513	positive regulation of BMP signaling pathway	0.621705	1
GO:0032728	positive regulation of interferon-beta production	0.621705	1
GO:0032814	regulation of natural killer cell activation	0.621705	1
GO:0032965	regulation of collagen biosynthetic process	0.621705	1
GO:0033260	nuclear DNA replication	0.621705	1
GO:0035337	fatty-acyl-CoA metabolic process	0.621705	1
GO:0042307	positive regulation of protein import into nucleus	0.621705	1
GO:0045738	negative regulation of DNA repair	0.621705	1
GO:0045981	positive regulation of nucleotide metabolic process	0.621705	1
GO:0050919	negative chemotaxis	0.621705	1
GO:0062098	regulation of programmed necrotic cell death	0.621705	1
GO:0071459	protein localization to chromosome, centromeric region	0.621705	1
GO:0071542	dopaminergic neuron differentiation	0.621705	1
GO:0072210	metanephric nephron development	0.621705	1

GO:1900544	positive regulation of purine nucleotide metabolic process	0.621705	1
GO:1990806	ligand-gated ion channel signaling pathway	0.621705	1
GO:1903829	positive regulation of protein localization	0.622877	8
GO:0007204	positive regulation of cytosolic calcium ion concentration	0.62569	3
GO:0008593	regulation of Notch signaling pathway	0.62569	2
GO:0032602	chemokine production	0.62569	2
GO:0032642	regulation of chemokine production	0.62569	2
GO:0042102	positive regulation of T cell proliferation	0.62569	2
GO:0009895	negative regulation of catabolic process	0.62569	6
GO:0006650	glycerophospholipid metabolic process	0.62569	5
GO:0000578	embryonic axis specification	0.62569	1
GO:0002691	regulation of cellular extravasation	0.62569	1
GO:0006479	protein methylation	0.62569	1
GO:0008213	protein alkylation	0.62569	1
GO:0009126	purine nucleoside monophosphate metabolic process	0.62569	1
GO:0030851	granulocyte differentiation	0.62569	1
GO:0031146	SCF-dependent proteasomal ubiquitin-dependent protein catabolic process	0.62569	1
GO:0032733	positive regulation of interleukin-10 production	0.62569	1
GO:0043903	regulation of biological process involved in symbiotic interaction	0.62569	1
GO:0045066	regulatory T cell differentiation	0.62569	1
GO:0045454	cell redox homeostasis	0.62569	1
GO:0046461	neutral lipid catabolic process	0.62569	1
GO:0046464	acylglycerol catabolic process	0.62569	1
GO:0046676	negative regulation of insulin secretion	0.62569	1
GO:0051955	regulation of amino acid transport	0.62569	1
GO:0060251	regulation of glial cell proliferation	0.62569	1
GO:0070232	regulation of T cell apoptotic process	0.62569	1
GO:0071354	cellular response to interleukin-6	0.62569	1
GO:1901532	regulation of hematopoietic progenitor cell differentiation	0.62569	1

GO:2000008	regulation of protein localization to cell surface	0.62569	1
GO:2001259	positive regulation of cation channel activity	0.62569	1
GO:0019318	hexose metabolic process	0.625779	4
GO:1903706	regulation of hemopoiesis	0.62613	7
GO:1901796	regulation of signal transduction by p53 class mediator	0.62613	2
GO:0044282	small molecule catabolic process	0.627284	6
GO:0006909	phagocytosis	0.629166	4
GO:0009259	ribonucleotide metabolic process	0.630276	6
GO:0002758	innate immune response-activating signaling pathway	0.630994	5
GO:0008637	apoptotic mitochondrial changes	0.630994	2
GO:0045639	positive regulation of myeloid cell differentiation	0.630994	2
GO:1990266	neutrophil migration	0.630994	2
GO:0002696	positive regulation of leukocyte activation	0.631321	6
GO:0009260	ribonucleotide biosynthetic process	0.631321	3
GO:0014904	myotube cell development	0.631321	1
GO:0043001	Golgi to plasma membrane protein transport	0.631321	1
GO:0043094	metabolic compound salvage	0.631321	1
GO:0043268	positive regulation of potassium ion transport	0.631321	1
GO:0043277	apoptotic cell clearance	0.631321	1
GO:0046640	regulation of alpha-beta T cell proliferation	0.631321	1
GO:0120033	negative regulation of plasma membrane bounded cell projection assembly	0.631321	1
GO:1900225	regulation of NLRP3 inflammasome complex assembly	0.631321	1
GO:2001258	negative regulation of cation channel activity	0.631321	1
GO:0002377	immunoglobulin production	0.633441	2
GO:0006626	protein targeting to mitochondrion	0.633441	2
GO:0021549	cerebellum development	0.633441	2
GO:0033045	regulation of sister chromatid segregation	0.633441	2
GO:0030308	negative regulation of cell growth	0.633441	3
GO:0030856	regulation of epithelial cell differentiation	0.633441	3
GO:0048284	organelle fusion	0.633441	3
GO:0050796	regulation of insulin secretion	0.633441	3

GO:0006820	monoatomic anion transport	0.636541	3
GO:0009266	response to temperature stimulus	0.636541	3
GO:0010389	regulation of G2/M transition of mitotic cell cycle	0.636541	2
GO:0009409	response to cold	0.636541	1
GO:0030201	heparan sulfate proteoglycan metabolic process	0.636541	1
GO:0030835	negative regulation of actin filament depolymerization	0.636541	1
GO:0031111	negative regulation of microtubule polymerization or depolymerization	0.636541	1
GO:0035794	positive regulation of mitochondrial membrane permeability	0.636541	1
GO:0045740	positive regulation of DNA replication	0.636541	1
GO:0046006	regulation of activated T cell proliferation	0.636541	1
GO:0046627	negative regulation of insulin receptor signaling pathway	0.636541	1
GO:0048821	erythrocyte development	0.636541	1
GO:0097178	ruffle assembly	0.636541	1
GO:0016054	organic acid catabolic process	0.636541	4
GO:0046395	carboxylic acid catabolic process	0.636541	4
GO:0046434	organophosphate catabolic process	0.636541	4
GO:0065004	protein-DNA complex assembly	0.636541	4
GO:0051402	neuron apoptotic process	0.637254	5
GO:0043604	amide biosynthetic process	0.638674	3
GO:0051701	biological process involved in interaction with host	0.638674	3
GO:1903320	regulation of protein modification by small protein conjugation or removal	0.639477	4
GO:0006641	triglyceride metabolic process	0.639477	2
GO:0010586	miRNA metabolic process	0.639477	2
GO:0031341	regulation of cell killing	0.639477	2
GO:0048144	fibroblast proliferation	0.639477	2
GO:0072676	lymphocyte migration	0.639477	2
GO:0007005	mitochondrion organization	0.639499	7

GO:0003044	regulation of systemic arterial blood pressure mediated by a chemical signal	0.639499	1
GO:0007007	inner mitochondrial membrane organization	0.639499	1
GO:0021517	ventral spinal cord development	0.639499	1
GO:0030574	collagen catabolic process	0.639499	1
GO:0032092	positive regulation of protein binding	0.639499	1
GO:0035196	miRNA processing	0.639499	1
GO:0044786	cell cycle DNA replication	0.639499	1
GO:0048701	embryonic cranial skeleton morphogenesis	0.639499	1
GO:0050691	regulation of defense response to virus by host	0.639499	1
GO:0061001	regulation of dendritic spine morphogenesis	0.639499	1
GO:0070296	sarcoplasmic reticulum calcium ion transport	0.639499	1
GO:0071402	cellular response to lipoprotein particle stimulus	0.639499	1
GO:0090148	membrane fission	0.639499	1
GO:1900077	negative regulation of cellular response to insulin stimulus	0.639499	1
GO:1901016	regulation of potassium ion transmembrane transporter activity	0.639499	1
GO:1902692	regulation of neuroblast proliferation	0.639499	1
GO:1990776	response to angiotensin	0.639499	1
GO:0006892	post-Golgi vesicle-mediated transport	0.640184	2
GO:0009308	amine metabolic process	0.640184	2
GO:0035249	synaptic transmission, glutamatergic	0.640184	2
GO:0048024	regulation of mRNA splicing, via spliceosome	0.640184	2
GO:1901655	cellular response to ketone	0.640184	2
GO:0019693	ribose phosphate metabolic process	0.640184	6
GO:0009743	response to carbohydrate	0.640454	4
GO:0072593	reactive oxygen species metabolic process	0.640454	4
GO:0007338	single fertilization	0.640454	3
GO:0007519	skeletal muscle tissue development	0.640454	3
GO:0045017	glycerolipid biosynthetic process	0.641377	4
GO:0071219	cellular response to molecule of bacterial origin	0.641377	4
GO:0140888	interferon-mediated signaling pathway	0.641377	2
GO:1901890	positive regulation of cell junction assembly	0.641377	2
GO:0006911	phagocytosis, engulfment	0.641377	1

GO:0010712	regulation of collagen metabolic process	0.641377	1
GO:0014014	negative regulation of gliogenesis	0.641377	1
GO:0021587	cerebellum morphogenesis	0.641377	1
GO:0021762	substantia nigra development	0.641377	1
GO:0030149	sphingolipid catabolic process	0.641377	1
GO:0031638	zymogen activation	0.641377	1
GO:0042398	modified amino acid biosynthetic process	0.641377	1
GO:0043300	regulation of leukocyte degranulation	0.641377	1
GO:0045646	regulation of erythrocyte differentiation	0.641377	1
GO:0045663	positive regulation of myoblast differentiation	0.641377	1
GO:0046633	alpha-beta T cell proliferation	0.641377	1
GO:0048483	autonomic nervous system development	0.641377	1
GO:0060443	mammary gland morphogenesis	0.641377	1
GO:0060563	neuroepithelial cell differentiation	0.641377	1
GO:0070741	response to interleukin-6	0.641377	1
GO:0095500	acetylcholine receptor signaling pathway	0.641377	1
GO:1903214	regulation of protein targeting to mitochondrion	0.641377	1
GO:1990000	amyloid fibril formation	0.641377	1
GO:1990573	potassium ion import across plasma membrane	0.641377	1
GO:0019362	pyridine nucleotide metabolic process	0.643756	3
GO:0046390	ribose phosphate biosynthetic process	0.643756	3
GO:0046496	nicotinamide nucleotide metabolic process	0.643756	3
GO:0042116	macrophage activation	0.644308	2
GO:0042752	regulation of circadian rhythm	0.644308	2
GO:0090277	positive regulation of peptide hormone secretion	0.644308	2
GO:0006644	phospholipid metabolic process	0.644897	6
GO:0071692	protein localization to extracellular region	0.644897	6
GO:1901873	regulation of post-translational protein modification	0.644897	4
GO:0031929	TOR signaling	0.645619	3
GO:0007157	heterophilic cell-cell adhesion via plasma membrane cell adhesion molecules	0.645619	1
GO:0007520	myoblast fusion	0.645619	1
GO:0010824	regulation of centrosome duplication	0.645619	1
GO:0031641	regulation of myelination	0.645619	1

GO:0042168	heme metabolic process	0.645619	1
GO:0042789	mRNA transcription by RNA polymerase II	0.645619	1
GO:0044273	sulfur compound catabolic process	0.645619	1
GO:0048247	lymphocyte chemotaxis	0.645619	1
GO:0050798	activated T cell proliferation	0.645619	1
GO:0060071	Wnt signaling pathway, planar cell polarity pathway	0.645619	1
GO:0090278	negative regulation of peptide hormone secretion	0.645619	1
GO:1903426	regulation of reactive oxygen species biosynthetic process	0.645619	1
GO:1905521	regulation of macrophage migration	0.645619	1
GO:0001776	leukocyte homeostasis	0.645879	2
GO:0038202	TORC1 signaling	0.645879	2
GO:0002793	positive regulation of peptide secretion	0.648955	2
GO:0036503	ERAD pathway	0.648955	2
GO:0048477	oogenesis	0.648955	2
GO:0051303	establishment of chromosome localization	0.648955	2
GO:0008654	phospholipid biosynthetic process	0.648955	4
GO:0001914	regulation of T cell mediated cytotoxicity	0.648955	1
GO:0002792	negative regulation of peptide secretion	0.648955	1
GO:0006862	nucleotide transport	0.648955	1
GO:0007602	phototransduction	0.648955	1
GO:0010634	positive regulation of epithelial cell migration	0.648955	1
GO:0010837	regulation of keratinocyte proliferation	0.648955	1
GO:0019674	NAD metabolic process	0.648955	1
GO:0032527	protein exit from endoplasmic reticulum	0.648955	1
GO:0033120	positive regulation of RNA splicing	0.648955	1
GO:0048066	developmental pigmentation	0.648955	1
GO:0070266	necroptotic process	0.648955	1
GO:0071526	semaphorin-plexin signaling pathway	0.648955	1
GO:0120163	negative regulation of cold-induced thermogenesis	0.648955	1
GO:0141085	regulation of inflammasome-mediated signaling pathway	0.648955	1

GO:1900271	regulation of long-term synaptic potentiation	0.648955	1
GO:1902175	regulation of oxidative stress-induced intrinsic apoptotic signaling pathway	0.648955	1
GO:1903587	regulation of blood vessel endothelial cell proliferation involved in sprouting angiogenesis	0.648955	1
GO:2000300	regulation of synaptic vesicle exocytosis	0.648955	1
GO:2000516	positive regulation of CD4-positive, alpha-beta T cell activation	0.648955	1
GO:0030336	negative regulation of cell migration	0.650007	6
GO:0045444	fat cell differentiation	0.650007	4
GO:0006109	regulation of carbohydrate metabolic process	0.650007	3
GO:1901292	nucleoside phosphate catabolic process	0.650007	3
GO:0002065	columnar/cuboidal epithelial cell differentiation	0.650007	2
GO:0018958	phenol-containing compound metabolic process	0.650007	2
GO:0070252	actin-mediated cell contraction	0.650007	2
GO:0070555	response to interleukin-1	0.650007	2
GO:0090263	positive regulation of canonical Wnt signaling pathway	0.650007	2
GO:0031400	negative regulation of protein modification process	0.652092	5
GO:0009135	purine nucleoside diphosphate metabolic process	0.652092	2
GO:0009179	purine ribonucleoside diphosphate metabolic process	0.652092	2
GO:0010821	regulation of mitochondrion organization	0.652092	2
GO:0021575	hindbrain morphogenesis	0.652092	1
GO:0033628	regulation of cell adhesion mediated by integrin	0.652092	1
GO:0042088	T-helper 1 type immune response	0.652092	1
GO:0043330	response to exogenous dsRNA	0.652092	1
GO:0046189	phenol-containing compound biosynthetic process	0.652092	1
GO:0046578	regulation of Ras protein signal transduction	0.652092	1
GO:0048489	synaptic vesicle transport	0.652092	1
GO:0072583	clathrin-dependent endocytosis	0.652092	1
GO:0090279	regulation of calcium ion import	0.652092	1

GO:1902041	regulation of extrinsic apoptotic signaling pathway via death domain receptors	0.652092	1
GO:1905710	positive regulation of membrane permeability	0.652092	1
GO:2000378	negative regulation of reactive oxygen species metabolic process	0.652092	1
GO:2000785	regulation of autophagosome assembly	0.652092	1
GO:1901293	nucleoside phosphate biosynthetic process	0.65219	5
GO:0034599	cellular response to oxidative stress	0.652917	4
GO:0002753	cytoplasmic pattern recognition receptor signaling pathway	0.654248	3
GO:0043523	regulation of neuron apoptotic process	0.655269	4
GO:0022037	metencephalon development	0.655269	2
GO:0033209	tumor necrosis factor-mediated signaling pathway	0.655269	2
GO:0042177	negative regulation of protein catabolic process	0.655269	2
GO:0050867	positive regulation of cell activation	0.655269	6
GO:0043484	regulation of RNA splicing	0.655269	3
GO:0072524	pyridine-containing compound metabolic process	0.655269	3
GO:0001774	microglial cell activation	0.655269	1
GO:0002347	response to tumor cell	0.655269	1
GO:0006024	glycosaminoglycan biosynthetic process	0.655269	1
GO:0006693	prostaglandin metabolic process	0.655269	1
GO:0021515	cell differentiation in spinal cord	0.655269	1
GO:0030521	androgen receptor signaling pathway	0.655269	1
GO:0031670	cellular response to nutrient	0.655269	1
GO:0042551	neuron maturation	0.655269	1
GO:0043114	regulation of vascular permeability	0.655269	1
GO:0045071	negative regulation of viral genome replication	0.655269	1
GO:0046686	response to cadmium ion	0.655269	1
GO:0047496	vesicle transport along microtubule	0.655269	1
GO:0048538	thymus development	0.655269	1
GO:0098815	modulation of excitatory postsynaptic potential	0.655269	1
GO:0140894	endolysosomal toll-like receptor signaling pathway	0.655269	1

GO:0002218	activation of innate immune response	0.656326	5
GO:0000045	autophagosome assembly	0.656326	2
GO:0031640	killing of cells of another organism	0.656326	2
GO:0099072	regulation of postsynaptic membrane neurotransmitter receptor levels	0.656326	2
GO:0141061	disruption of cell in another organism	0.656326	2
GO:0031345	negative regulation of cell projection organization	0.656788	3
GO:1901136	carbohydrate derivative catabolic process	0.659637	4
GO:0007006	mitochondrial membrane organization	0.659637	2
GO:1902106	negative regulation of leukocyte differentiation	0.659637	2
GO:0006120	mitochondrial electron transport, NADH to ubiquinone	0.659637	1
GO:0006692	prostanoid metabolic process	0.659637	1
GO:0007019	microtubule depolymerization	0.659637	1
GO:0008089	anterograde axonal transport	0.659637	1
GO:0008206	bile acid metabolic process	0.659637	1
GO:0008542	visual learning	0.659637	1
GO:0035384	thioester biosynthetic process	0.659637	1
GO:0045214	sarcomere organization	0.659637	1
GO:0060119	inner ear receptor cell development	0.659637	1
GO:0071616	acyl-CoA biosynthetic process	0.659637	1
GO:0035725	sodium ion transmembrane transport	0.662421	3
GO:0001838	embryonic epithelial tube formation	0.66308	2
GO:0042303	molting cycle	0.66308	2
GO:0042633	hair cycle	0.66308	2
GO:0043414	macromolecule methylation	0.66308	2
GO:0006790	sulfur compound metabolic process	0.66382	5
GO:0001764	neuron migration	0.66382	3
GO:0016241	regulation of macroautophagy	0.66382	3
GO:0001974	blood vessel remodeling	0.66382	1
GO:0003197	endocardial cushion development	0.66382	1
GO:0009299	mRNA transcription	0.66382	1
GO:0010171	body morphogenesis	0.66382	1

GO:0030838	positive regulation of actin filament polymerization	0.66382	1
GO:0035987	endodermal cell differentiation	0.66382	1
GO:0043457	regulation of cellular respiration	0.66382	1
GO:0046466	membrane lipid catabolic process	0.66382	1
GO:0050856	regulation of T cell receptor signaling pathway	0.66382	1
GO:1903747	regulation of establishment of protein localization to mitochondrion	0.66382	1
GO:1905562	regulation of vascular endothelial cell proliferation	0.66382	1
GO:0006720	isoprenoid metabolic process	0.664559	2
GO:0071621	granulocyte chemotaxis	0.664559	2
GO:1902749	regulation of cell cycle G2/M phase transition	0.664559	2
GO:0050870	positive regulation of T cell activation	0.664559	4
GO:0045580	regulation of T cell differentiation	0.665156	3
GO:0048771	tissue remodeling	0.665156	3
GO:0060538	skeletal muscle organ development	0.665156	3
GO:1901991	negative regulation of mitotic cell cycle phase transition	0.665156	3
GO:0030301	cholesterol transport	0.666676	2
GO:0050000	chromosome localization	0.666676	2
GO:0141060	disruption of anatomical structure in another organism	0.666676	2
GO:0061025	membrane fusion	0.666676	3
GO:0002269	leukocyte activation involved in inflammatory response	0.666676	1
GO:0006497	protein lipidation	0.666676	1
GO:0007129	homologous chromosome pairing at meiosis	0.666676	1
GO:0021695	cerebellar cortex development	0.666676	1
GO:0022602	ovulation cycle process	0.666676	1
GO:0030834	regulation of actin filament depolymerization	0.666676	1
GO:0031648	protein destabilization	0.666676	1
GO:0042269	regulation of natural killer cell mediated cytotoxicity	0.666676	1

GO:0060688	regulation of morphogenesis of a branching structure	0.666676	1
GO:0098926	postsynaptic signal transduction	0.666676	1
GO:0101023	vascular endothelial cell proliferation	0.666676	1
GO:1901570	fatty acid derivative biosynthetic process	0.666676	1
GO:0030510	regulation of BMP signaling pathway	0.67035	2
GO:0000209	protein polyubiquitination	0.670555	4
GO:2000146	negative regulation of cell motility	0.670807	6
GO:0016072	rRNA metabolic process	0.671882	4
GO:0002455	humoral immune response mediated by circulating immunoglobulin	0.671882	1
GO:0006778	porphyrin-containing compound metabolic process	0.671882	1
GO:0008608	attachment of spindle microtubules to kinetochore	0.671882	1
GO:0009395	phospholipid catabolic process	0.671882	1
GO:0010543	regulation of platelet activation	0.671882	1
GO:0048146	positive regulation of fibroblast proliferation	0.671882	1
GO:0048286	lung alveolus development	0.671882	1
GO:0071320	cellular response to cAMP	0.671882	1
GO:2000772	regulation of cellular senescence	0.671882	1
GO:0008202	steroid metabolic process	0.672607	5
GO:0061640	cytoskeleton-dependent cytokinesis	0.67646	2
GO:1905037	autophagosome organization	0.67646	2
GO:0022409	positive regulation of cell-cell adhesion	0.67646	5
GO:0002040	sprouting angiogenesis	0.67646	3
GO:0002043	blood vessel endothelial cell proliferation involved in sprouting angiogenesis	0.67646	1
GO:0006110	regulation of glycolytic process	0.67646	1
GO:0030811	regulation of nucleotide catabolic process	0.67646	1
GO:0032924	activin receptor signaling pathway	0.67646	1
GO:0033121	regulation of purine nucleotide catabolic process	0.67646	1
GO:0042743	hydrogen peroxide metabolic process	0.67646	1
GO:0046365	monosaccharide catabolic process	0.67646	1

GO:0072538	T-helper 17 type immune response	0.67646	1
GO:0099024	plasma membrane invagination	0.67646	1
GO:1903351	cellular response to dopamine	0.67646	1
GO:0030833	regulation of actin filament polymerization	0.679178	2
GO:0072655	establishment of protein localization to mitochondrion	0.679178	2
GO:1903707	negative regulation of hemopoiesis	0.679178	2
GO:0001738	morphogenesis of a polarized epithelium	0.681408	1
GO:0007187	G protein-coupled receptor signaling pathway, coupled to cyclic nucleotide second messenger	0.681408	1
GO:0007528	neuromuscular junction development	0.681408	1
GO:0010719	negative regulation of epithelial to mesenchymal transition	0.681408	1
GO:0030857	negative regulation of epithelial cell differentiation	0.681408	1
GO:0046605	regulation of centrosome cycle	0.681408	1
GO:0085029	extracellular matrix assembly	0.681408	1
GO:0086065	cell communication involved in cardiac conduction	0.681408	1
GO:1902895	positive regulation of miRNA transcription	0.681408	1
GO:0009185	ribonucleoside diphosphate metabolic process	0.681408	2
GO:0030278	regulation of ossification	0.681408	2
GO:0030282	bone mineralization	0.681408	2
GO:0006913	nucleocytoplasmic transport	0.681408	5
GO:0051169	nuclear transport	0.681408	5
GO:0002686	negative regulation of leukocyte migration	0.686285	1
GO:0014888	striated muscle adaptation	0.686285	1
GO:0031529	ruffle organization	0.686285	1
GO:0043331	response to dsRNA	0.686285	1
GO:0050805	negative regulation of synaptic transmission	0.686285	1
GO:0051452	intracellular pH reduction	0.686285	1
GO:1901799	negative regulation of proteasomal protein catabolic process	0.686285	1
GO:1903350	response to dopamine	0.686285	1
GO:0009154	purine ribonucleotide catabolic process	0.68913	2

GO:0055001	muscle cell development	0.689711	3
GO:0006091	generation of precursor metabolites and energy	0.689711	7
GO:0045787	positive regulation of cell cycle	0.689711	5
GO:0000288	nuclear-transcribed mRNA catabolic process, deadenylation-dependent decay	0.689711	1
GO:0001755	neural crest cell migration	0.689711	1
GO:0006023	aminoglycan biosynthetic process	0.689711	1
GO:0006826	iron ion transport	0.689711	1
GO:0015804	neutral amino acid transport	0.689711	1
GO:0030837	negative regulation of actin filament polymerization	0.689711	1
GO:0043462	regulation of ATP-dependent activity	0.689711	1
GO:0061178	regulation of insulin secretion involved in cellular response to glucose stimulus	0.689711	1
GO:0071868	cellular response to monoamine stimulus	0.689711	1
GO:0071870	cellular response to catecholamine stimulus	0.689711	1
GO:0072132	mesenchyme morphogenesis	0.689711	1
GO:0006401	RNA catabolic process	0.689711	6
GO:0090068	positive regulation of cell cycle process	0.689711	4
GO:0043200	response to amino acid	0.689711	2
GO:0050830	defense response to Gram-positive bacterium	0.689711	2
GO:0051209	release of sequestered calcium ion into cytosol	0.689711	2
GO:0006399	tRNA metabolic process	0.690069	3
GO:0051650	establishment of vesicle localization	0.692021	3
GO:0015918	sterol transport	0.692021	2
GO:0051283	negative regulation of sequestering of calcium ion	0.692021	2
GO:0001913	T cell mediated cytotoxicity	0.692021	1
GO:0002715	regulation of natural killer cell mediated immunity	0.692021	1
GO:0007632	visual behavior	0.692021	1
GO:0009161	ribonucleoside monophosphate metabolic process	0.692021	1
GO:0009948	anterior/posterior axis specification	0.692021	1
GO:0016073	snRNA metabolic process	0.692021	1

GO:0019748	secondary metabolic process	0.692021	1
GO:0032413	negative regulation of ion transmembrane transporter activity	0.692021	1
GO:0034332	adherens junction organization	0.692021	1
GO:0042158	lipoprotein biosynthetic process	0.692021	1
GO:0045005	DNA-templated DNA replication maintenance of fidelity	0.692021	1
GO:0045912	negative regulation of carbohydrate metabolic process	0.692021	1
GO:0046902	regulation of mitochondrial membrane permeability	0.692021	1
GO:0060986	endocrine hormone secretion	0.692021	1
GO:0061900	glial cell activation	0.692021	1
GO:0086009	membrane repolarization	0.692021	1
GO:0002708	positive regulation of lymphocyte mediated immunity	0.694058	2
GO:0050684	regulation of mRNA processing	0.694058	2
GO:0098773	skin epidermis development	0.694058	2
GO:0060828	regulation of canonical Wnt signaling pathway	0.694058	4
GO:0071695	anatomical structure maturation	0.694058	4
GO:0001541	ovarian follicle development	0.696009	1
GO:0009116	nucleoside metabolic process	0.696009	1
GO:0042733	embryonic digit morphogenesis	0.696009	1
GO:0043616	keratinocyte proliferation	0.696009	1
GO:0051480	regulation of cytosolic calcium ion concentration	0.696009	1
GO:0060350	endochondral bone morphogenesis	0.696009	1
GO:0060997	dendritic spine morphogenesis	0.696009	1
GO:0070613	regulation of protein processing	0.696009	1
GO:0090497	mesenchymal cell migration	0.696009	1
GO:0009132	nucleoside diphosphate metabolic process	0.696009	2
GO:0051282	regulation of sequestering of calcium ion	0.696009	2
GO:0040013	negative regulation of locomotion	0.697191	6
GO:0030073	insulin secretion	0.697191	3
GO:0090150	establishment of protein localization to membrane	0.697523	4

GO:0007041	lysosomal transport	0.699242	2
GO:0008033	tRNA processing	0.699242	2
GO:0070585	protein localization to mitochondrion	0.699242	2
GO:0001706	endoderm formation	0.699242	1
GO:0038093	Fc receptor signaling pathway	0.699242	1
GO:0042306	regulation of protein import into nucleus	0.699242	1
GO:0043113	receptor clustering	0.699242	1
GO:0044088	regulation of vacuole organization	0.699242	1
GO:0070231	T cell apoptotic process	0.699242	1
GO:0071867	response to monoamine	0.699242	1
GO:0071869	response to catecholamine	0.699242	1
GO:0097300	programmed necrotic cell death	0.699242	1
GO:0051098	regulation of binding	0.70133	3
GO:0002821	positive regulation of adaptive immune response	0.70133	2
GO:0009261	ribonucleotide catabolic process	0.70133	2
GO:0140014	mitotic nuclear division	0.702526	4
GO:0001933	negative regulation of protein phosphorylation	0.702526	3
GO:0000768	syncytium formation by plasma membrane fusion	0.702526	1
GO:0007004	telomere maintenance via telomerase	0.702526	1
GO:0010324	membrane invagination	0.702526	1
GO:0022029	telencephalon cell migration	0.702526	1
GO:0031663	lipopolysaccharide-mediated signaling pathway	0.702526	1
GO:0060261	positive regulation of transcription initiation by RNA polymerase II	0.702526	1
GO:0070228	regulation of lymphocyte apoptotic process	0.702526	1
GO:0071709	membrane assembly	0.702526	1
GO:0099518	vesicle cytoskeletal trafficking	0.702526	1
GO:0140253	cell-cell fusion	0.702526	1
GO:1903409	reactive oxygen species biosynthetic process	0.702526	1
GO:0007093	mitotic cell cycle checkpoint signaling	0.702532	2
GO:0051983	regulation of chromosome segregation	0.702532	2
GO:0051208	sequestering of calcium ion	0.706912	2
GO:0010257	NADH dehydrogenase complex assembly	0.707519	1

GO:0032981	mitochondrial respiratory chain complex I assembly	0.707519	1
GO:0033059	cellular pigmentation	0.707519	1
GO:0034113	heterotypic cell-cell adhesion	0.707519	1
GO:1905517	macrophage migration	0.707519	1
GO:0007266	Rho protein signal transduction	0.709629	2
GO:0030048	actin filament-based movement	0.709629	2
GO:0051592	response to calcium ion	0.709629	2
GO:0002763	positive regulation of myeloid leukocyte differentiation	0.71192	1
GO:0033619	membrane protein proteolysis	0.71192	1
GO:0045058	T cell selection	0.71192	1
GO:0046824	positive regulation of nucleocytoplasmic transport	0.71192	1
GO:0097352	autophagosome maturation	0.71192	1
GO:0097479	synaptic vesicle localization	0.71192	1
GO:1901607	alpha-amino acid biosynthetic process	0.71192	1
GO:0034728	nucleosome organization	0.712121	2
GO:0050777	negative regulation of immune response	0.712121	3
GO:0050911	detection of chemical stimulus involved in sensory perception of smell	0.715033	6
GO:0002011	morphogenesis of an epithelial sheet	0.715033	1
GO:0006518	peptide metabolic process	0.715033	1
GO:0006903	vesicle targeting	0.715033	1
GO:0006956	complement activation	0.715033	1
GO:0021885	forebrain cell migration	0.715033	1
GO:0022617	extracellular matrix disassembly	0.715033	1
GO:0032757	positive regulation of interleukin-8 production	0.715033	1
GO:0040014	regulation of multicellular organism growth	0.715033	1
GO:0048814	regulation of dendrite morphogenesis	0.715033	1
GO:0070192	chromosome organization involved in meiotic cell cycle	0.715033	1
GO:1903078	positive regulation of protein localization to plasma membrane	0.715033	1

GO:2001244	positive regulation of intrinsic apoptotic signaling pathway	0.715033	1
GO:0006949	syncytium formation	0.71918	1
GO:0008631	intrinsic apoptotic signaling pathway in response to oxidative stress	0.71918	1
GO:0043470	regulation of carbohydrate catabolic process	0.71918	1
GO:0045744	negative regulation of G protein-coupled receptor signaling pathway	0.71918	1
GO:0051289	protein homotetramerization	0.71918	1
GO:0070050	neuron cellular homeostasis	0.71918	1
GO:0090303	positive regulation of wound healing	0.71918	1
GO:1903317	regulation of protein maturation	0.71918	1
GO:1905330	regulation of morphogenesis of an epithelium	0.71918	1
GO:2000351	regulation of endothelial cell apoptotic process	0.71918	1
GO:0000086	G2/M transition of mitotic cell cycle	0.719777	2
GO:0002819	regulation of adaptive immune response	0.721709	3
GO:0006695	cholesterol biosynthetic process	0.721709	1
GO:0030219	megakaryocyte differentiation	0.721709	1
GO:0032623	interleukin-2 production	0.721709	1
GO:0032663	regulation of interleukin-2 production	0.721709	1
GO:0034394	protein localization to cell surface	0.721709	1
GO:0034605	cellular response to heat	0.721709	1
GO:0042058	regulation of epidermal growth factor receptor signaling pathway	0.721709	1
GO:0045143	homologous chromosome segregation	0.721709	1
GO:0045185	maintenance of protein location	0.721709	1
GO:0045453	bone resorption	0.721709	1
GO:0050832	defense response to fungus	0.721709	1
GO:0051123	RNA polymerase II preinitiation complex assembly	0.721709	1
GO:1902653	secondary alcohol biosynthetic process	0.721709	1
GO:2000630	positive regulation of miRNA metabolic process	0.721709	1
GO:0051054	positive regulation of DNA metabolic process	0.721709	4
GO:0031333	negative regulation of protein-containing complex assembly	0.724217	2

GO:2000779	regulation of double-strand break repair	0.724217	2
GO:0009583	detection of light stimulus	0.725944	1
GO:0016925	protein sumoylation	0.725944	1
GO:0035773	insulin secretion involved in cellular response to glucose stimulus	0.725944	1
GO:0042776	proton motive force-driven mitochondrial ATP synthesis	0.725944	1
GO:0051445	regulation of meiotic cell cycle	0.725944	1
GO:0070918	regulatory ncRNA processing	0.725944	1
GO:0001101	response to acid chemical	0.726366	2
GO:0001889	liver development	0.726366	2
GO:1900180	regulation of protein localization to nucleus	0.726366	2
GO:0050727	regulation of inflammatory response	0.728131	6
GO:0045598	regulation of fat cell differentiation	0.729264	2
GO:0006278	RNA-templated DNA biosynthetic process	0.729264	1
GO:0035567	non-canonical Wnt signaling pathway	0.729264	1
GO:0044091	membrane biogenesis	0.729264	1
GO:0046503	glycerolipid catabolic process	0.729264	1
GO:0048857	neural nucleus development	0.729264	1
GO:0098930	axonal transport	0.729264	1
GO:2000144	positive regulation of DNA-templated transcription initiation	0.729264	1
GO:0141188	nucleic acid catabolic process	0.730931	6
GO:0051235	maintenance of location	0.732444	3
GO:0051648	vesicle localization	0.732444	3
GO:0009306	protein secretion	0.732444	5
GO:0043627	response to estrogen	0.73387	1
GO:0048662	negative regulation of smooth muscle cell proliferation	0.73387	1
GO:0045619	regulation of lymphocyte differentiation	0.734405	3
GO:0061008	hepaticobiliary system development	0.734405	2
GO:0090090	negative regulation of canonical Wnt signaling pathway	0.734405	2
GO:0097530	granulocyte migration	0.734405	2
GO:0042326	negative regulation of phosphorylation	0.736965	3

GO:0099111	microtubule-based transport	0.736965	3
GO:1903828	negative regulation of protein localization	0.736965	3
GO:0008203	cholesterol metabolic process	0.737506	2
GO:0018107	peptidyl-threonine phosphorylation	0.737506	1
GO:0035592	establishment of protein localization to extracellular region	0.737506	5
GO:0051223	regulation of protein transport	0.739108	6
GO:0042180	ketone metabolic process	0.739108	3
GO:0051056	regulation of small GTPase mediated signal transduction	0.741588	4
GO:0002711	positive regulation of T cell mediated immunity	0.741588	1
GO:0006303	double-strand break repair via nonhomologous end joining	0.741588	1
GO:0072577	endothelial cell apoptotic process	0.741588	1
GO:2000134	negative regulation of G1/S transition of mitotic cell cycle	0.741588	1
GO:0030534	adult behavior	0.743224	2
GO:0008652	amino acid biosynthetic process	0.745641	1
GO:0032272	negative regulation of protein polymerization	0.745641	1
GO:0050766	positive regulation of phagocytosis	0.745641	1
GO:0051898	negative regulation of phosphatidylinositol 3-kinase/protein kinase B signal transduction	0.745641	1
GO:0060260	regulation of transcription initiation by RNA polymerase II	0.745641	1
GO:0071901	negative regulation of protein serine/threonine kinase activity	0.745641	1
GO:0006997	nucleus organization	0.749448	2
GO:0016126	sterol biosynthetic process	0.750065	1
GO:0030858	positive regulation of epithelial cell differentiation	0.750065	1
GO:1901184	regulation of ERBB signaling pathway	0.750065	1
GO:2000036	regulation of stem cell population maintenance	0.750065	1
GO:0032006	regulation of TOR signaling	0.751718	2
GO:1903038	negative regulation of leukocyte cell-cell adhesion	0.751718	2

GO:0045926	negative regulation of growth	0.751718	3
GO:0006959	humoral immune response	0.751718	4
GO:0006406	mRNA export from nucleus	0.751718	1
GO:0010833	telomere maintenance via telomere lengthening	0.751718	1
GO:0021879	forebrain neuron differentiation	0.751718	1
GO:0030239	myofibril assembly	0.751718	1
GO:0042130	negative regulation of T cell proliferation	0.751718	1
GO:0042698	ovulation cycle	0.751718	1
GO:0043112	receptor metabolic process	0.751718	1
GO:0046635	positive regulation of alpha-beta T cell activation	0.751718	1
GO:0051881	regulation of mitochondrial membrane potential	0.751718	1
GO:0090559	regulation of membrane permeability	0.751718	1
GO:0002449	lymphocyte mediated immunity	0.751718	5
GO:0007030	Golgi organization	0.751718	2
GO:0030041	actin filament polymerization	0.751718	2
GO:0031023	microtubule organizing center organization	0.751718	2
GO:2000377	regulation of reactive oxygen species metabolic process	0.751718	2
GO:0032481	positive regulation of type I interferon production	0.753878	1
GO:0045123	cellular extravasation	0.753878	1
GO:0045911	positive regulation of DNA recombination	0.753878	1
GO:0050709	negative regulation of protein secretion	0.753878	1
GO:0050795	regulation of behavior	0.753878	1
GO:0051298	centrosome duplication	0.753878	1
GO:0062208	positive regulation of pattern recognition receptor signaling pathway	0.753878	1
GO:0070527	platelet aggregation	0.753878	1
GO:1902893	regulation of miRNA transcription	0.753878	1
GO:1903578	regulation of ATP metabolic process	0.753878	1
GO:2000242	negative regulation of reproductive process	0.753878	1
GO:0009123	nucleoside monophosphate metabolic process	0.757053	1
GO:0015986	proton motive force-driven ATP synthesis	0.757053	1
GO:0032371	regulation of sterol transport	0.757053	1

GO:0032374	regulation of cholesterol transport	0.757053	1
GO:0046470	phosphatidylcholine metabolic process	0.757053	1
GO:0051966	regulation of synaptic transmission, glutamatergic	0.757053	1
GO:0061614	miRNA transcription	0.757053	1
GO:1900076	regulation of cellular response to insulin stimulus	0.757053	1
GO:1904377	positive regulation of protein localization to cell periphery	0.757053	1
GO:0007033	vacuole organization	0.759758	3
GO:0035264	multicellular organism growth	0.759758	2
GO:0044839	cell cycle G2/M phase transition	0.759758	2
GO:0050807	regulation of synapse organization	0.759758	4
GO:0007585	respiratory gaseous exchange by respiratory system	0.759758	1
GO:0009620	response to fungus	0.759758	1
GO:0032729	positive regulation of type II interferon production	0.759758	1
GO:0050853	B cell receptor signaling pathway	0.759758	1
GO:0070373	negative regulation of ERK1 and ERK2 cascade	0.759758	1
GO:1903533	regulation of protein targeting	0.759758	1
GO:0007608	sensory perception of smell	0.759758	6
GO:0016125	sterol metabolic process	0.762461	2
GO:1902652	secondary alcohol metabolic process	0.762461	2
GO:0015931	nucleobase-containing compound transport	0.763387	3
GO:0009247	glycolipid biosynthetic process	0.76359	1
GO:1901568	fatty acid derivative metabolic process	0.76359	1
GO:0006261	DNA-templated DNA replication	0.765106	2
GO:0002437	inflammatory response to antigenic stimulus	0.767672	1
GO:0031016	pancreas development	0.767672	1
GO:0140962	multicellular organismal-level chemical homeostasis	0.767672	1
GO:0002460	adaptive immune response based on somatic recombination of immune receptors built from immunoglobulin superfamily domains	0.770239	5

GO:0099173	postsynapse organization	0.770239	3
GO:0001510	RNA methylation	0.770239	1
GO:0018210	peptidyl-threonine modification	0.770239	1
GO:0032410	negative regulation of transporter activity	0.770239	1
GO:0042440	pigment metabolic process	0.770239	1
GO:0043954	cellular component maintenance	0.770239	1
GO:0070897	transcription preinitiation complex assembly	0.770239	1
GO:1902807	negative regulation of cell cycle G1/S phase transition	0.770239	1
GO:1903036	positive regulation of response to wounding	0.770239	1
GO:0050803	regulation of synapse structure or activity	0.770266	4
GO:0010976	positive regulation of neuron projection development	0.771621	2
GO:0050657	nucleic acid transport	0.771621	2
GO:0050658	RNA transport	0.771621	2
GO:0002224	toll-like receptor signaling pathway	0.773431	1
GO:0030500	regulation of bone mineralization	0.773431	1
GO:2000142	regulation of DNA-templated transcription initiation	0.773431	1
GO:0045930	negative regulation of mitotic cell cycle	0.775118	3
GO:0016311	dephosphorylation	0.776538	2
GO:0006446	regulation of translational initiation	0.776538	1
GO:0008088	axo-dendritic transport	0.776538	1
GO:0030203	glycosaminoglycan metabolic process	0.776538	1
GO:0031424	keratinization	0.776538	1
GO:2000027	regulation of animal organ morphogenesis	0.776538	1
GO:0051236	establishment of RNA localization	0.779231	2
GO:0001701	in utero embryonic development	0.779562	5
GO:0001523	retinoid metabolic process	0.779562	1
GO:0008344	adult locomotory behavior	0.779562	1
GO:0021872	forebrain generation of neurons	0.779562	1
GO:0051279	regulation of release of sequestered calcium ion into cytosol	0.779562	1
GO:2000736	regulation of stem cell differentiation	0.779562	1
GO:0051963	regulation of synapse assembly	0.780896	2

GO:0050907	detection of chemical stimulus involved in sensory perception	0.781354	6
GO:0007018	microtubule-based movement	0.782983	6
GO:0046928	regulation of neurotransmitter secretion	0.782983	1
GO:2000514	regulation of CD4-positive, alpha-beta T cell activation	0.782983	1
GO:0043409	negative regulation of MAPK cascade	0.786278	2
GO:0035023	regulation of Rho protein signal transduction	0.787048	1
GO:0006979	response to oxidative stress	0.787987	5
GO:0007034	vacuolar transport	0.788315	2
GO:0007292	female gamete generation	0.788315	2
GO:0043087	regulation of GTPase activity	0.788315	2
GO:0048678	response to axon injury	0.789349	1
GO:0055013	cardiac muscle cell development	0.789349	1
GO:0150076	neuroinflammatory response	0.789349	1
GO:1900542	regulation of purine nucleotide metabolic process	0.789349	1
GO:1901379	regulation of potassium ion transmembrane transport	0.789349	1
GO:0007264	small GTPase-mediated signal transduction	0.791661	6
GO:0055002	striated muscle cell development	0.792296	2
GO:0006140	regulation of nucleotide metabolic process	0.792296	1
GO:0006900	vesicle budding from membrane	0.792296	1
GO:0032024	positive regulation of insulin secretion	0.792296	1
GO:0034103	regulation of tissue remodeling	0.792296	1
GO:0071897	DNA biosynthetic process	0.794941	2
GO:0006094	gluconeogenesis	0.795167	1
GO:0016101	diterpenoid metabolic process	0.795167	1
GO:0043543	protein acylation	0.795167	1
GO:0098586	cellular response to virus	0.795167	1
GO:1901264	carbohydrate derivative transport	0.795167	1
GO:0030509	BMP signaling pathway	0.796596	2
GO:0048145	regulation of fibroblast proliferation	0.798692	1
GO:2000243	positive regulation of reproductive process	0.798692	1
GO:0032635	interleukin-6 production	0.798702	2

GO:0032675	regulation of interleukin-6 production	0.798702	2
GO:0046849	bone remodeling	0.80166	1
GO:0050672	negative regulation of lymphocyte proliferation	0.80166	1
GO:0051591	response to cAMP	0.80166	1
GO:0016052	carbohydrate catabolic process	0.803789	2
GO:0019319	hexose biosynthetic process	0.804558	1
GO:0031123	RNA 3'-end processing	0.804558	1
GO:0032273	positive regulation of protein polymerization	0.804558	1
GO:0060021	roof of mouth development	0.804558	1
GO:0048285	organelle fission	0.806429	6
GO:0006400	tRNA modification	0.807386	1
GO:0019646	aerobic electron transport chain	0.807386	1
GO:0032945	negative regulation of mononuclear cell proliferation	0.807386	1
GO:0072384	organelle transport along microtubule	0.807386	1
GO:0021954	central nervous system neuron development	0.810391	1
GO:0033273	response to vitamin	0.810391	1
GO:0046364	monosaccharide biosynthetic process	0.810391	1
GO:2000781	positive regulation of double-strand break repair	0.810391	1
GO:0090316	positive regulation of intracellular protein transport	0.814067	1
GO:0006575	modified amino acid metabolic process	0.814734	2
GO:0006405	RNA export from nucleus	0.817189	1
GO:0045132	meiotic chromosome segregation	0.817189	1
GO:0032886	regulation of microtubule-based process	0.818263	3
GO:0000075	cell cycle checkpoint signaling	0.819388	2
GO:0006096	glycolytic process	0.820001	1
GO:0033555	multicellular organismal response to stress	0.820001	1
GO:0010563	negative regulation of phosphorus metabolic process	0.821458	3
GO:0044843	cell cycle G1/S phase transition	0.821458	3
GO:0045936	negative regulation of phosphate metabolic process	0.821458	3
GO:0006022	aminoglycan metabolic process	0.822502	1
GO:0042157	lipoprotein metabolic process	0.822502	1

GO:0071772	response to BMP	0.822685	2
GO:0071773	cellular response to BMP stimulus	0.822685	2
GO:0007416	synapse assembly	0.824077	3
GO:0032386	regulation of intracellular transport	0.824077	3
GO:0050708	regulation of protein secretion	0.824077	3
GO:1901988	negative regulation of cell cycle phase transition	0.824077	3
GO:0007032	endosome organization	0.824196	1
GO:0046032	ADP catabolic process	0.824196	1
GO:0043488	regulation of mRNA stability	0.824345	3
GO:0007565	female pregnancy	0.824345	2
GO:0051302	regulation of cell division	0.824345	2
GO:0006637	acyl-CoA metabolic process	0.824345	1
GO:0006721	terpenoid metabolic process	0.824345	1
GO:0006754	ATP biosynthetic process	0.824345	1
GO:0031110	regulation of microtubule polymerization or depolymerization	0.824345	1
GO:0035383	thioester metabolic process	0.824345	1
GO:0043648	dicarboxylic acid metabolic process	0.824345	1
GO:0045824	negative regulation of innate immune response	0.824345	1
GO:0051262	protein tetramerization	0.824345	1
GO:0070664	negative regulation of leukocyte proliferation	0.824345	1
GO:0070936	protein K48-linked ubiquitination	0.824345	1
GO:1903432	regulation of TORC1 signaling	0.824345	1
GO:0006402	mRNA catabolic process	0.825297	4
GO:0062207	regulation of pattern recognition receptor signaling pathway	0.825297	2
GO:0097305	response to alcohol	0.825917	3
GO:1901990	regulation of mitotic cell cycle phase transition	0.825917	4
GO:0034109	homotypic cell-cell adhesion	0.825917	1
GO:0042773	ATP synthesis coupled electron transport	0.825917	1
GO:0042775	mitochondrial ATP synthesis coupled electron transport	0.825917	1
GO:0071229	cellular response to acid chemical	0.825917	1
GO:0000070	mitotic sister chromatid segregation	0.827675	2
GO:0003073	regulation of systemic arterial blood pressure	0.827675	1

GO:0009137	purine nucleoside diphosphate catabolic process	0.827675	1
GO:0009181	purine ribonucleoside diphosphate catabolic process	0.827675	1
GO:0048640	negative regulation of developmental growth	0.827675	1
GO:0050764	regulation of phagocytosis	0.827675	1
GO:0051099	positive regulation of binding	0.827675	1
GO:0000910	cytokinesis	0.829371	2
GO:0061157	mRNA destabilization	0.829371	2
GO:0006260	DNA replication	0.831118	3
GO:1902107	positive regulation of leukocyte differentiation	0.831166	2
GO:1903708	positive regulation of hemopoiesis	0.831166	2
GO:0000041	transition metal ion transport	0.832499	1
GO:0009408	response to heat	0.832499	1
GO:0043266	regulation of potassium ion transport	0.832499	1
GO:0051258	protein polymerization	0.833872	3
GO:0009191	ribonucleoside diphosphate catabolic process	0.834084	1
GO:0032637	interleukin-8 production	0.834084	1
GO:0032677	regulation of interleukin-8 production	0.834084	1
GO:0046031	ADP metabolic process	0.834084	1
GO:1901992	positive regulation of mitotic cell cycle phase transition	0.834084	1
GO:1990830	cellular response to leukemia inhibitory factor	0.834084	1
GO:0050779	RNA destabilization	0.835639	2
GO:0071466	cellular response to xenobiotic stimulus	0.835639	2
GO:0002027	regulation of heart rate	0.836109	1
GO:0002312	B cell activation involved in immune response	0.836109	1
GO:0002709	regulation of T cell mediated immunity	0.836109	1
GO:0000280	nuclear division	0.836375	5
GO:0032271	regulation of protein polymerization	0.836375	2
GO:0061014	positive regulation of mRNA catabolic process	0.836375	2
GO:0010721	negative regulation of cell development	0.836375	3
GO:1990823	response to leukemia inhibitory factor	0.838079	1
GO:0002706	regulation of lymphocyte mediated immunity	0.838235	2
GO:0043487	regulation of RNA stability	0.839253	3

GO:0001938	positive regulation of endothelial cell proliferation	0.839253	1
GO:0007631	feeding behavior	0.839253	1
GO:0009134	nucleoside diphosphate catabolic process	0.839253	1
GO:0019751	polyol metabolic process	0.839253	1
GO:0043393	regulation of protein binding	0.839253	1
GO:0097306	cellular response to alcohol	0.839253	1
GO:1901605	alpha-amino acid metabolic process	0.843292	2
GO:0061013	regulation of mRNA catabolic process	0.843646	3
GO:0016236	macroautophagy	0.844172	4
GO:0031398	positive regulation of protein ubiquitination	0.844172	1
GO:0090398	cellular senescence	0.844172	1
GO:0044772	mitotic cell cycle phase transition	0.844693	5
GO:0016064	immunoglobulin mediated immune response	0.844693	2
GO:1902806	regulation of cell cycle G1/S phase transition	0.844693	2
GO:2000241	regulation of reproductive process	0.844693	2
GO:0001676	long-chain fatty acid metabolic process	0.844693	1
GO:0007040	lysosome organization	0.844693	1
GO:0007229	integrin-mediated signaling pathway	0.844693	1
GO:0009206	purine ribonucleoside triphosphate biosynthetic process	0.844693	1
GO:0042632	cholesterol homeostasis	0.844693	1
GO:0080171	lytic vacuole organization	0.844693	1
GO:0051321	meiotic cell cycle	0.845878	3
GO:0006403	RNA localization	0.845878	2
GO:0007051	spindle organization	0.845878	2
GO:0009145	purine nucleoside triphosphate biosynthetic process	0.846157	1
GO:0019218	regulation of steroid metabolic process	0.846157	1
GO:0055092	sterol homeostasis	0.846157	1
GO:0019724	B cell mediated immunity	0.849297	2
GO:0051651	maintenance of location in cell	0.857603	2
GO:1901987	regulation of cell cycle phase transition	0.858546	5
GO:0009201	ribonucleoside triphosphate biosynthetic process	0.858758	1

GO:0022904	respiratory electron transport chain	0.858758	1
GO:0043547	positive regulation of GTPase activity	0.858758	1
GO:0001824	blastocyst development	0.860947	1
GO:0007265	Ras protein signal transduction	0.860947	1
GO:0030518	nuclear receptor-mediated steroid hormone signaling pathway	0.863094	1
GO:0046822	regulation of nucleocytoplasmic transport	0.863094	1
GO:0031503	protein-containing complex localization	0.863538	2
GO:0048193	Golgi vesicle transport	0.864441	3
GO:0006334	nucleosome assembly	0.864441	1
GO:0016579	protein deubiquitination	0.864441	1
GO:0032355	response to estradiol	0.864441	1
GO:0030522	intracellular receptor signaling pathway	0.866504	4
GO:1902414	protein localization to cell junction	0.866504	1
GO:0033108	mitochondrial respiratory chain complex assembly	0.868526	1
GO:0045582	positive regulation of T cell differentiation	0.868526	1
GO:1903046	meiotic cell cycle process	0.869111	2
GO:0046634	regulation of alpha-beta T cell activation	0.870006	1
GO:0051224	negative regulation of protein transport	0.870006	1
GO:0006486	protein glycosylation	0.870006	2
GO:0043413	macromolecule glycosylation	0.870006	2
GO:0006364	rRNA processing	0.870929	2
GO:0000956	nuclear-transcribed mRNA catabolic process	0.870929	1
GO:0006413	translational initiation	0.870929	1
GO:0032609	type II interferon production	0.870929	1
GO:0032649	regulation of type II interferon production	0.870929	1
GO:0032259	methylation	0.872237	2
GO:0009142	nucleoside triphosphate biosynthetic process	0.872831	1
GO:0010948	negative regulation of cell cycle process	0.874696	3
GO:1901989	positive regulation of cell cycle phase transition	0.874696	1
GO:0006090	pyruvate metabolic process	0.876268	1
GO:0006612	protein targeting to membrane	0.876268	1
GO:0022900	electron transport chain	0.876268	1
GO:1903311	regulation of mRNA metabolic process	0.877141	4

GO:0006457	protein folding	0.877207	2
GO:0050866	negative regulation of cell activation	0.877207	2
GO:0045786	negative regulation of cell cycle	0.877745	4
GO:0039531	regulation of cytoplasmic pattern recognition receptor signaling pathway	0.879557	1
GO:0002824	positive regulation of adaptive immune response based on somatic recombination of immune receptors built from immunoglobulin superfamily domains	0.881168	1
GO:0007127	meiosis I	0.881168	1
GO:0046034	ATP metabolic process	0.881168	2
GO:0098813	nuclear chromosome segregation	0.882011	3
GO:0034502	protein localization to chromosome	0.882446	1
GO:0046718	symbiont entry into host cell	0.882446	1
GO:0006302	double-strand break repair	0.882705	3
GO:1903322	positive regulation of protein modification by small protein conjugation or removal	0.886091	1
GO:0000819	sister chromatid segregation	0.886091	2
GO:1903313	positive regulation of mRNA metabolic process	0.886091	2
GO:1901875	positive regulation of post-translational protein modification	0.887544	1
GO:0002832	negative regulation of response to biotic stimulus	0.888836	1
GO:0007052	mitotic spindle organization	0.888836	1
GO:0043401	steroid hormone receptor signaling pathway	0.888836	1
GO:0044409	symbiont entry into host	0.889325	1
GO:0045931	positive regulation of mitotic cell cycle	0.889325	1
GO:0050868	negative regulation of T cell activation	0.889325	1
GO:0051028	mRNA transport	0.889325	1
GO:0051225	spindle assembly	0.889325	1
GO:0061844	antimicrobial humoral immune response mediated by antimicrobial peptide	0.889325	1
GO:1904951	positive regulation of establishment of protein localization	0.890327	3
GO:0002456	T cell mediated immunity	0.890551	1

GO:0045621	positive regulation of lymphocyte differentiation	0.890551	1
GO:0000082	G1/S transition of mitotic cell cycle	0.891745	2
GO:0042445	hormone metabolic process	0.891745	2
GO:0018205	peptidyl-lysine modification	0.891745	1
GO:0070085	glycosylation	0.892651	2
GO:0050768	negative regulation of neurogenesis	0.892651	1
GO:0070646	protein modification by small protein removal	0.892651	1
GO:1904950	negative regulation of establishment of protein localization	0.892651	1
GO:0032388	positive regulation of intracellular transport	0.893784	1
GO:0045727	positive regulation of translation	0.893784	1
GO:0061982	meiosis I cell cycle process	0.893784	1
GO:0033044	regulation of chromosome organization	0.89544	2
GO:0045333	cellular respiration	0.896215	2
GO:0000018	regulation of DNA recombination	0.896215	1
GO:0018105	peptidyl-serine phosphorylation	0.896215	1
GO:0031109	microtubule polymerization or depolymerization	0.896215	1
GO:0007098	centrosome cycle	0.897516	1
GO:0010212	response to ionizing radiation	0.897516	1
GO:0007059	chromosome segregation	0.898876	4
GO:0051961	negative regulation of nervous system development	0.903988	1
GO:0099175	regulation of postsynapse organization	0.905411	1
GO:0009205	purine ribonucleoside triphosphate metabolic process	0.906697	2
GO:0050714	positive regulation of protein secretion	0.90818	1
GO:0006119	oxidative phosphorylation	0.909527	1
GO:0051222	positive regulation of protein transport	0.909774	2
GO:0046165	alcohol biosynthetic process	0.910589	1
GO:0018209	peptidyl-serine modification	0.913421	1
GO:0009144	purine nucleoside triphosphate metabolic process	0.913673	2
GO:0009199	ribonucleoside triphosphate metabolic process	0.913673	2
GO:0031507	heterochromatin formation	0.921373	1
GO:0000723	telomere maintenance	0.923587	1

GO:0030010	establishment of cell polarity	0.923587	1
GO:0006066	alcohol metabolic process	0.923605	3
GO:0120254	olefinic compound metabolic process	0.927017	1
GO:0070507	regulation of microtubule cytoskeleton organization	0.928032	1
GO:0170033	L-amino acid metabolic process	0.928763	1
GO:0170039	proteinogenic amino acid metabolic process	0.928763	1
GO:0009141	nucleoside triphosphate metabolic process	0.928965	2
GO:0140588	chromatin looping	0.929373	1
GO:0002181	cytoplasmic translation	0.929373	1
GO:0006367	transcription initiation at RNA polymerase II promoter	0.929373	1
GO:0006694	steroid biosynthetic process	0.929373	1
GO:0007584	response to nutrient	0.929373	1
GO:1902850	microtubule cytoskeleton organization involved in mitosis	0.929373	1
GO:0009451	RNA modification	0.931494	1
GO:0006520	amino acid metabolic process	0.93267	2
GO:0051250	negative regulation of lymphocyte activation	0.933279	1
GO:0051168	nuclear export	0.934145	1
GO:2000045	regulation of G1/S transition of mitotic cell cycle	0.93609	1
GO:0008380	RNA splicing	0.936218	4
GO:0010970	transport along microtubule	0.940802	1
GO:0141193	nuclear receptor-mediated signaling pathway	0.942524	1
GO:0019730	antimicrobial humoral response	0.944187	1
GO:0032200	telomere organization	0.947608	1
GO:0031348	negative regulation of defense response	0.9503	2
GO:0040029	epigenetic regulation of gene expression	0.9503	2
GO:0140013	meiotic nuclear division	0.953544	1
GO:0042254	ribosome biogenesis	0.95572	2
GO:0009060	aerobic respiration	0.956081	1
GO:0002695	negative regulation of leukocyte activation	0.957728	1
GO:0002822	regulation of adaptive immune response based on somatic recombination of immune receptors built from immunoglobulin superfamily domains	0.957728	1

GO:0045814	negative regulation of gene expression, epigenetic	0.958862	1
GO:0030705	cytoskeleton-dependent intracellular transport	0.959006	1
GO:0050728	negative regulation of inflammatory response	0.959006	1
GO:0006352	DNA-templated transcription initiation	0.961379	1
GO:0017148	negative regulation of translation	0.962371	1
GO:0021953	central nervous system neuron differentiation	0.963325	1
GO:0000377	RNA splicing, via transesterification reactions with bulged adenosine as nucleophile	0.963383	2
GO:0000398	mRNA splicing, via spliceosome	0.963383	2
GO:0000375	RNA splicing, via transesterification reactions	0.965055	2
GO:0009755	hormone-mediated signaling pathway	0.966783	1
GO:0009410	response to xenobiotic stimulus	0.967209	3
GO:0022618	protein-RNA complex assembly	0.968812	1
GO:0071826	protein-RNA complex organization	0.972711	1
GO:0022613	ribonucleoprotein complex biogenesis	0.979469	3
GO:0031669	cellular response to nutrient levels	0.979954	1
GO:0009636	response to toxic substance	0.983659	1
GO:0006310	DNA recombination	0.995251	1
GO:0006417	regulation of translation	0.998885	1

- **Table S3.** Commonly upregulated KEGG pathways (full list).

ID	Description	p.adjust	Count
hsa05030	Cocaine addiction	0.374727	2
hsa04750	Inflammatory mediator regulation of TRP channels	0.374727	2
hsa00564	Glycerophospholipid metabolism	0.374727	2
hsa04064	NF-kappa B signaling pathway	0.374727	2
hsa00533	Glycosaminoglycan biosynthesis - keratan sulfate	0.374727	1
hsa04722	Neurotrophin signaling pathway	0.374727	2
hsa00360	Phenylalanine metabolism	0.374727	1
hsa00531	Glycosaminoglycan degradation	0.374727	1
hsa00340	Histidine metabolism	0.374727	1
hsa04390	Hippo signaling pathway	0.374727	2
hsa04514	Cell adhesion molecules	0.374727	2
hsa04392	Hippo signaling pathway - multiple species	0.41038	1

hsa04621	NOD-like receptor signaling pathway	0.424565	2
hsa00350	Tyrosine metabolism	0.433146	1
hsa00260	Glycine, serine and threonine metabolism	0.439124	1
hsa00380	Tryptophan metabolism	0.439124	1
hsa00330	Arginine and proline metabolism	0.469099	1
hsa00565	Ether lipid metabolism	0.469099	1
hsa05031	Amphetamine addiction	0.504753	1
hsa00982	Drug metabolism - cytochrome P450	0.504753	1
hsa05212	Pancreatic cancer	0.504753	1
hsa05133	Pertussis	0.504753	1
hsa04721	Synaptic vesicle cycle	0.504753	1
hsa05140	Leishmaniasis	0.504753	1
hsa04742	Taste transduction	0.504753	1
hsa04912	GnRH signaling pathway	0.504753	1
hsa04666	Fc gamma R-mediated phagocytosis	0.504753	1
hsa05231	Choline metabolism in cancer	0.504753	1
hsa05142	Chagas disease	0.504753	1
hsa04620	Toll-like receptor signaling pathway	0.504753	1
hsa04350	TGF-beta signaling pathway	0.504753	1
hsa05145	Toxoplasmosis	0.504753	1
hsa04726	Serotonergic synapse	0.504753	1
hsa04928	Parathyroid hormone synthesis, secretion and action	0.504753	1
hsa04724	Glutamatergic synapse	0.504753	1
hsa04071	Sphingolipid signaling pathway	0.504753	1
hsa04728	Dopaminergic synapse	0.504753	1
hsa04142	Lysosome	0.504753	1
hsa05135	Yersinia infection	0.504753	1
hsa05162	Measles	0.504753	1
hsa04550	Signaling pathways regulating pluripotency of stem cells	0.504753	1
hsa04936	Alcoholic liver disease	0.504753	1
hsa04072	Phospholipase D signaling pathway	0.504753	1
hsa05226	Gastric cancer	0.504753	1
hsa04148	Efferocytosis	0.512576	1

hsa05161	Hepatitis B	0.514713	1
hsa05164	Influenza A	0.514713	1
hsa05152	Tuberculosis	0.514713	1
hsa05168	Herpes simplex virus 1 infection	0.514713	1
hsa05034	Alcoholism	0.514713	1
hsa04082	Neuroactive ligand signaling	0.514713	1
hsa05202	Transcriptional misregulation in cancer	0.514713	1
hsa04510	Focal adhesion	0.514713	1
hsa05130	Pathogenic Escherichia coli infection	0.514713	1
hsa05169	Epstein-Barr virus infection	0.514713	1
hsa05170	Human immunodeficiency virus 1 infection	0.514713	1
hsa05417	Lipid and atherosclerosis	0.514713	1
hsa04382	Cornified envelope formation	0.514713	1
hsa04024	cAMP signaling pathway	0.514886	1
hsa05208	Chemical carcinogenesis - reactive oxygen species	0.514886	1
hsa04014	Ras signaling pathway	0.516268	1
hsa05171	Coronavirus disease - COVID-19	0.516268	1
hsa05132	Salmonella infection	0.521664	1
hsa04144	Endocytosis	0.521664	1
hsa05012	Parkinson disease	0.541291	1
hsa04010	MAPK signaling pathway	0.572333	1
hsa05165	Human papillomavirus infection	0.604616	1
hsa04151	PI3K-Akt signaling pathway	0.627932	1
hsa04080	Neuroactive ligand-receptor interaction	0.627932	1
hsa05010	Alzheimer disease	0.640253	1
hsa04740	Olfactory transduction	0.68813	1
hsa05022	Pathways of neurodegeneration - multiple diseases	0.70163	1

- **Table S4.** Commonly downregulated KEGG pathways (full list).

ID	Description	p.adjust	Count
hsa04015	Rap1 signaling pathway	0.000859	14
hsa04024	cAMP signaling pathway	0.000903	14
hsa04014	Ras signaling pathway	0.001088	14
hsa04810	Regulation of actin cytoskeleton	0.011854	12
hsa04072	Phospholipase D signaling pathway	0.020047	9

hsa04010	MAPK signaling pathway	0.023876	13
hsa04151	PI3K-Akt signaling pathway	0.036853	14
hsa04066	HIF-1 signaling pathway	0.036853	7
hsa05418	Fluid shear stress and atherosclerosis	0.036853	8
hsa04670	Leukocyte transendothelial migration	0.042541	7
hsa04923	Regulation of lipolysis in adipocytes	0.042541	5
hsa04976	Bile secretion	0.043496	6
hsa04960	Aldosterone-regulated sodium reabsorption	0.043496	4
hsa04662	B cell receptor signaling pathway	0.043496	6
hsa04925	Aldosterone synthesis and secretion	0.05825	6
hsa04924	Renin secretion	0.05825	5
hsa05135	Yersinia infection	0.068548	7
hsa04020	Calcium signaling pathway	0.068548	10
hsa05224	Breast cancer	0.087426	7
hsa01521	EGFR tyrosine kinase inhibitor resistance	0.087426	5
hsa05226	Gastric cancer	0.088324	7
hsa05205	Proteoglycans in cancer	0.133729	8
hsa04022	cGMP-PKG signaling pathway	0.133729	7
hsa04213	Longevity regulating pathway - multiple species	0.133729	4
hsa04713	Circadian entrainment	0.133729	5
hsa04270	Vascular smooth muscle contraction	0.133729	6
hsa04070	Phosphatidylinositol signaling system	0.133729	5
hsa05417	Lipid and atherosclerosis	0.133729	8
hsa04929	GnRH secretion	0.133729	4
hsa05231	Choline metabolism in cancer	0.133729	5
hsa04916	Melanogenesis	0.133729	5
hsa04915	Estrogen signaling pathway	0.133729	6
hsa05221	Acute myeloid leukemia	0.133729	4
hsa05142	Chagas disease	0.133729	5
hsa04664	Fc epsilon RI signaling pathway	0.133729	4
hsa04064	NF-kappa B signaling pathway	0.133729	5
hsa04625	C-type lectin receptor signaling pathway	0.133729	5
hsa05211	Renal cell carcinoma	0.133729	4
hsa04360	Axon guidance	0.133729	7
hsa05215	Prostate cancer	0.133729	5

hsa05230	Central carbon metabolism in cancer	0.133729	4
hsa05218	Melanoma	0.142939	4
hsa04971	Gastric acid secretion	0.159042	4
hsa04261	Adrenergic signaling in cardiomyocytes	0.162166	6
hsa04921	Oxytocin signaling pathway	0.162166	6
hsa02010	ABC transporters	0.162166	3
hsa04725	Cholinergic synapse	0.162166	5
hsa05160	Hepatitis C	0.163949	6
hsa04930	Type II diabetes mellitus	0.163949	3
hsa04510	Focal adhesion	0.163949	7
hsa04722	Neurotrophin signaling pathway	0.163949	5
hsa04146	Peroxisome	0.163949	4
hsa04071	Sphingolipid signaling pathway	0.163949	5
hsa04152	AMPK signaling pathway	0.163949	5
hsa04660	T cell receptor signaling pathway	0.163949	5
hsa04919	Thyroid hormone signaling pathway	0.163949	5
hsa04672	Intestinal immune network for IgA production	0.17127	3
hsa04012	ErbB signaling pathway	0.17127	4
hsa04911	Insulin secretion	0.17127	4
hsa04973	Carbohydrate digestion and absorption	0.179724	3
hsa04512	ECM-receptor interaction	0.181582	4
hsa04926	Relaxin signaling pathway	0.181582	5
hsa04964	Proximal tubule bicarbonate reclamation	0.181582	2
hsa04211	Longevity regulating pathway	0.181582	4
hsa04912	GnRH signaling pathway	0.194737	4
hsa04650	Natural killer cell mediated cytotoxicity	0.194737	5
hsa04080	Neuroactive ligand-receptor interaction	0.194778	10
hsa04657	IL-17 signaling pathway	0.200117	4
hsa05163	Human cytomegalovirus infection	0.200117	7
hsa04977	Vitamin digestion and absorption	0.203554	2
hsa04970	Salivary secretion	0.203554	4
hsa04370	VEGF signaling pathway	0.203554	3
hsa01040	Biosynthesis of unsaturated fatty acids	0.203554	2
hsa01522	Endocrine resistance	0.203554	4
hsa04666	Fc gamma R-mediated phagocytosis	0.203554	4

hsa04750	Inflammatory mediator regulation of TRP channels	0.203554	4
hsa04621	NOD-like receptor signaling pathway	0.212631	6
hsa04744	Phototransduction	0.224478	2
hsa04062	Chemokine signaling pathway	0.224478	6
hsa04927	Cortisol synthesis and secretion	0.231917	3
hsa04972	Pancreatic secretion	0.233015	4
hsa04082	Neuroactive ligand signaling	0.241911	6
hsa04060	Cytokine-cytokine receptor interaction	0.241911	8
hsa05031	Amphetamine addiction	0.249066	3
hsa05130	Pathogenic Escherichia coli infection	0.249066	6
hsa04914	Progesterone-mediated oocyte maturation	0.249066	4
hsa05131	Shigellosis	0.249066	7
hsa04130	SNARE interactions in vesicular transport	0.249066	2
hsa04150	mTOR signaling pathway	0.252089	5
hsa04917	Prolactin signaling pathway	0.253148	3
hsa04514	Cell adhesion molecules	0.256778	5
hsa04918	Thyroid hormone synthesis	0.2805	3
hsa05214	Glioma	0.281718	3
hsa04382	Cornified envelope formation	0.281718	6
hsa05207	Chemical carcinogenesis - receptor activation	0.281718	6
hsa05212	Pancreatic cancer	0.281718	3
hsa04935	Growth hormone synthesis, secretion and action	0.281718	4
hsa04081	Hormone signaling	0.281718	6
hsa05100	Bacterial invasion of epithelial cells	0.281718	3
hsa05133	Pertussis	0.281718	3
hsa04721	Synaptic vesicle cycle	0.286913	3
hsa05164	Influenza A	0.29274	5
hsa05166	Human T-cell leukemia virus 1 infection	0.29274	6
hsa04611	Platelet activation	0.29274	4
hsa05033	Nicotine addiction	0.30041	2
hsa05165	Human papillomavirus infection	0.304111	8
hsa05010	Alzheimer disease	0.314674	9
hsa04728	Dopaminergic synapse	0.318508	4
hsa00920	Sulfur metabolism	0.321867	1
hsa04260	Cardiac muscle contraction	0.321867	3

hsa05210	Colorectal cancer	0.321867	3
hsa04610	Complement and coagulation cascades	0.326834	3
hsa05235	PD-L1 expression and PD-1 checkpoint pathway in cancer	0.338146	3
hsa04910	Insulin signaling pathway	0.338146	4
hsa04540	Gap junction	0.349449	3
hsa03272	Virion - Hepatitis viruses	0.350181	2
hsa04520	Adherens junction	0.351267	3
hsa04380	Osteoclast differentiation	0.35438	4
hsa04550	Signaling pathways regulating pluripotency of stem cells	0.35438	4
hsa05017	Spinocerebellar ataxia	0.35438	4
hsa05323	Rheumatoid arthritis	0.35438	3
hsa05167	Kaposi sarcoma-associated herpesvirus infection	0.35438	5
hsa05132	Salmonella infection	0.359789	6
hsa04979	Cholesterol metabolism	0.360027	2
hsa04913	Ovarian steroidogenesis	0.367982	2
hsa04723	Retrograde endocannabinoid signaling	0.369909	4
hsa04640	Hematopoietic cell lineage	0.375142	3
hsa04933	AGE-RAGE signaling pathway in diabetic complications	0.379682	3
hsa05415	Diabetic cardiomyopathy	0.379839	5
hsa00600	Sphingolipid metabolism	0.384106	2
hsa05146	Amoebiasis	0.384106	3
hsa04934	Cushing syndrome	0.384106	4
hsa04340	Hedgehog signaling pathway	0.384106	2
hsa05134	Legionellosis	0.384106	2
hsa04218	Cellular senescence	0.388601	4
hsa01212	Fatty acid metabolism	0.388601	2
hsa04217	Necroptosis	0.396829	4
hsa05170	Human immunodeficiency virus 1 infection	0.398672	5
hsa05213	Endometrial cancer	0.400055	2
hsa04620	Toll-like receptor signaling pathway	0.400055	3
hsa04931	Insulin resistance	0.400055	3
hsa00450	Selenocompound metabolism	0.400202	1
hsa05161	Hepatitis B	0.402542	4
hsa04978	Mineral absorption	0.40556	2

hsa04980	Cobalamin transport and metabolism	0.409534	1
hsa04330	Notch signaling pathway	0.409534	2
hsa05020	Prion disease	0.409534	6
hsa04630	JAK-STAT signaling pathway	0.416387	4
hsa04530	Tight junction	0.42004	4
hsa05225	Hepatocellular carcinoma	0.42004	4
hsa04724	Glutamatergic synapse	0.42004	3
hsa00541	Biosynthesis of various nucleotide sugars	0.430471	1
hsa04668	TNF signaling pathway	0.434463	3
hsa00532	Glycosaminoglycan biosynthesis - chondroitin sulfate / dermatan sulfate	0.442947	1
hsa04614	Renin-angiotensin system	0.475187	1
hsa05223	Non-small cell lung cancer	0.481822	2
hsa00230	Purine metabolism	0.482193	3
hsa00534	Glycosaminoglycan biosynthesis - heparan sulfate / heparin	0.482995	1
hsa01524	Platinum drug resistance	0.490233	2
hsa05220	Chronic myeloid leukemia	0.499166	2
hsa04068	FoxO signaling pathway	0.499166	3
hsa04142	Lysosome	0.499166	3
hsa00562	Inositol phosphate metabolism	0.503704	2
hsa00062	Fatty acid elongation	0.52459	1
hsa00790	Folate biosynthesis	0.52459	1
hsa05206	MicroRNAs in cancer	0.526595	6
hsa04371	Apelin signaling pathway	0.526595	3
hsa04623	Cytosolic DNA-sensing pathway	0.526595	2
hsa04392	Hippo signaling pathway - multiple species	0.526595	1
hsa05202	Transcriptional misregulation in cancer	0.526595	4
hsa01523	Antifolate resistance	0.534808	1
hsa04740	Olfactory transduction	0.539338	8
hsa00030	Pentose phosphate pathway	0.539338	1
hsa04981	Folate transport and metabolism	0.539338	1
hsa05310	Asthma	0.549723	1
hsa04727	GABAergic synapse	0.55155	2
hsa05032	Morphine addiction	0.563181	2

hsa03020	RNA polymerase	0.563181	1
hsa04710	Circadian rhythm	0.563181	1
hsa04658	Th1 and Th2 cell differentiation	0.565615	2
hsa05222	Small cell lung cancer	0.565615	2
hsa00350	Tyrosine metabolism	0.573	1
hsa00512	Mucin type O-glycan biosynthesis	0.573	1
hsa04932	Non-alcoholic fatty liver disease	0.573	3
hsa00760	Nicotinate and nicotinamide metabolism	0.592262	1
hsa05410	Hypertrophic cardiomyopathy	0.592262	2
hsa04061	Viral protein interaction with cytokine and cytokine receptor	0.594352	2
hsa05330	Allograft rejection	0.594352	1
hsa00260	Glycine, serine and threonine metabolism	0.613112	1
hsa04140	Autophagy - animal	0.613112	3
hsa04974	Protein digestion and absorption	0.613112	2
hsa05414	Dilated cardiomyopathy	0.613112	2
hsa03022	Basal transcription factors	0.629517	1
hsa04310	Wnt signaling pathway	0.629517	3
hsa04350	TGF-beta signaling pathway	0.635011	2
hsa00514	Other types of O-glycan biosynthesis	0.65099	1
hsa04726	Serotonergic synapse	0.655336	2
hsa04928	Parathyroid hormone synthesis, secretion and action	0.655336	2
hsa05168	Herpes simplex virus 1 infection	0.656213	3
hsa05030	Cocaine addiction	0.656336	1
hsa00330	Arginine and proline metabolism	0.658725	1
hsa05144	Malaria	0.658725	1
hsa05110	Vibrio cholerae infection	0.664192	1
hsa00270	Cysteine and methionine metabolism	0.669474	1
hsa04961	Endocrine and other factor-regulated calcium reabsorption	0.674578	1
hsa05320	Autoimmune thyroid disease	0.679506	1
hsa04613	Neutrophil extracellular trap formation	0.687218	3
hsa00240	Pyrimidine metabolism	0.704268	1
hsa05022	Pathways of neurodegeneration - multiple diseases	0.710496	7
hsa05169	Epstein-Barr virus infection	0.710496	3

hsa05203	Viral carcinogenesis	0.710496	3
hsa04210	Apoptosis	0.716877	2
hsa03420	Nucleotide excision repair	0.716877	1
hsa05217	Basal cell carcinoma	0.716877	1
hsa00190	Oxidative phosphorylation	0.716877	2
hsa04114	Oocyte meiosis	0.716877	2
hsa05162	Measles	0.716877	2
hsa00561	Glycerolipid metabolism	0.720056	1
hsa05321	Inflammatory bowel disease	0.723202	1
hsa04720	Long-term potentiation	0.725673	1
hsa04936	Alcoholic liver disease	0.725673	2
hsa05416	Viral myocarditis	0.737877	1
hsa05120	Epithelial cell signaling in Helicobacter pylori infection	0.740426	1
hsa05208	Chemical carcinogenesis - reactive oxygen species	0.745997	3
hsa03320	PPAR signaling pathway	0.753621	1
hsa04820	Cytoskeleton in muscle cells	0.753621	3
hsa04148	Efferocytosis	0.753621	2
hsa04390	Hippo signaling pathway	0.753621	2
hsa04714	Thermogenesis	0.753621	3
hsa05016	Huntington disease	0.753621	4
hsa05171	Coronavirus disease - COVID-19	0.756967	3
hsa04612	Antigen processing and presentation	0.768312	1
hsa01232	Nucleotide metabolism	0.776621	1
hsa04742	Taste transduction	0.776621	1
hsa05412	Arrhythmogenic right ventricular cardiomyopathy	0.776621	1
hsa05152	Tuberculosis	0.808694	2
hsa05012	Parkinson disease	0.821464	3
hsa05034	Alcoholism	0.821464	2
hsa05150	Staphylococcus aureus infection	0.821464	1
hsa00564	Glycerophospholipid metabolism	0.821464	1
hsa03015	mRNA surveillance pathway	0.821464	1
hsa04922	Glucagon signaling pathway	0.830101	1
hsa03013	Nucleocytoplasmic transport	0.830101	1
hsa04659	Th17 cell differentiation	0.830101	1
hsa05014	Amyotrophic lateral sclerosis	0.831646	4

hsa04144	Endocytosis	0.911385	2
hsa04110	Cell cycle	0.919359	1
hsa04145	Phagosome	0.919359	1
hsa04141	Protein processing in endoplasmic reticulum	0.930598	1
hsa04814	Motor proteins	0.951079	1
